# Supplementary material for: Visible-light-driven reversible shuttle vicinal dihalogenation using lead halide perovskite quantum dot catalysts
Source: Nat Commun. 2023 Aug 4;14:4673. doi: 10.1038/s41467-023-40359-x (PMC10400542; doi:10.1038/s41467-023-40359-x)
Supplement: Supplementary file 1 — Supplementary Information [file 41467_2023_40359_MOESM1_ESM.pdf]

Supplementary Information

Visible-Light-Driven Reversible Shuttle Vicinal Dihalogenation Using Lead Halide Perovskite Quantum Dot Catalysts

Yonglong Li[1], Yangxuan Gao[1], Zhijie Deng[2,3], Yutao Cao[1], Teng Wang[1], Ying Wang[1], Cancan Zhang[1,4], Mingjian Yuan[1] and Wei Xie[1]*

[1]*Key Laboratory of Advanced Energy Materials Chemistry (Ministry of Education), Haihe Laboratory of Sustainable Chemical Transformations, Renewable Energy Conversion and Storage Center, College of Chemistry, Nankai University, Tianjin 300071, P. R. China.*

[2]*State Key Laboratory of Elemento-Organic Chemistry, College of Chemistry, Nankai University, Tianjin 300071, P. R. China.*

[3]*Mount Sinai Center for Therapeutics Discovery, Departments of Pharmacological Sciences, Oncological Sciences and Neuroscience, Tisch Cancer Institute, Icahn School of Medicine at Mount Sinai, New York, NY, 10029, USA.*

[4]*State Key Laboratory of Marine Resource Utilization in South China Sea, Hainan Provincial Key Laboratory of Research on Utilization of Si-Zr-Ti Resources, College of Materials Science and Engineering, Hainan University, Haikou, 570228, P. R. China.*

*wei.xie@nankai.edu.cn

Table of Content

1. General information (materials and characterization) 3

2. Light source parameters...5

3. Detection of ethylene product in dibromination reaction 6

4. Characterization of QD catalysts after reaction 8

5. Reversible vicinal dibromination experiments 9

6. Cost calculations..10

7. Survey of kinetic process for the dibromination...11

8. Ex-situ PL spectra of $CsPbBr_3$ QDs in DCM and DCM+DBE..............................12

9. Radical trapping experiments for the dibromination reaction..............................13

10. ATRA reaction mechanism...14

11. Optimized Cu loading in dichlorination 14

12. Characterization of Cu-$CsPbBr_3$ QDs 15

13. Crude ^{1}H NMR of the dichlorination reaction...16

14. Radical trapping experiment for the dichlorination reaction..............................17

15. GC graphs of hetero-dihalogenation mixture 18

16. Radical trapping experiment for the hetero-dihalogenation...............................19

17. Ex-situ PL spectra of Cu-QDs in TCE and TCE+DBE...20

18. Crude ^{1}H NMR of the hetero-dihalogenation and dibromination reaction 21

19. Determining turnover number (TON) and apparent quantum yield (AQY).....23

20. Optimization experiments 25

21. Spectral data for products...32

22. Supplementary references..63

1. General information (materials and characterization)

Commercial reagents

All reagents including the alkenes were purchased at commercial quality and directly used as received from commercial sources without further purification. The alkenes with heterocycles were synthesized using corresponding commercial carboxylic acids and phenols.

Supplementary Table 1. Reagent list.

Name	CAS	Supplier	Purity
Cesium carbonate	534-17-8	Sigma-Aldrich	99.95%
Copper bromide	7789-45-9	Heowns	99%
Lead (II) bromide	10031-22-8	Aladdin	99.999%
Oleic acid (OA)	112-80-1	Sigma-Aldrich	tech. 90%
Oleylamine (OAm)	112-90-3	Acros Organics	approximate C_{18}-content 80-90%
1-Octadecene	112-88-9	Sigma-Aldrich	tech. 90%
1,2-dibromoethane (DBE)	106-93-4	Meryer	99%
1,1,2,2-tetrachloroethane (TCE)	79-34-5	TCI	>97.0%
n-Hexane	110-54-3	Innochem	98%
Ethyl acetate	141-78-6	RHAWN	GR, 99.7%
Dichloromethane (DCM, SuperDry, with molecular sieves)	75-09-2	J&K	99.9%
Acetonitrile (MeCN, SuperDry, with molecular sieves)	75-05-8	J&K	99.9%

Characterization

High-resolution transmission electron microscopy (HR-TEM) and scanning transmission electron microscopy (STEM) in high angle angular dark field (HAADF) mode for elemental mapping images of the perovskite QDs were recorded with FEI Talos F200X G2 (AEMC) instrument with an acceleration voltage of 200 kV. Photoluminescence (PL) spectra of the QD catalysts were measured with a FS5 fluorescence spectrometer from

Edinburgh Instruments. Extinction spectra of the QDs were taken with an analytikjena UV-Vis absorption spectrometer (SPECORD 2010 plus). Pump-probe transient absorption (TA) spectra of the QD catalysts were measured by using a Helios spectrometer coupled with an ultrafast laser system (Coherent Astrella). X-ray photoelectron spectra (XPS) of the Cu doped QDs were collected on a Thermo Escalab 250Xi+ spectrometer. Inductively coupled plasma optical emission spectrometer (ICP-OES) measurement was conducted with an Agilent 7700s spectrometer. Photocatalytic transfer dihalogenations were carried out with a 120 W white LED with 10 channels (PL-SX100A, Beijing Precise Technology Co., Ltd.). NMR spectra were measured on a Bruker AVANCE AV 400 MHz spectrometer. The conversions of products were determined by using a FULI 9790 II GC and an Angilent 7890B-5977A/B or Scion 456-GC-SQ GC-MS, respectively. HRMS was conducted with a Bruker autoflex maX MALDI-TOF/TOF mass spectrometer.

2. Light source parameters

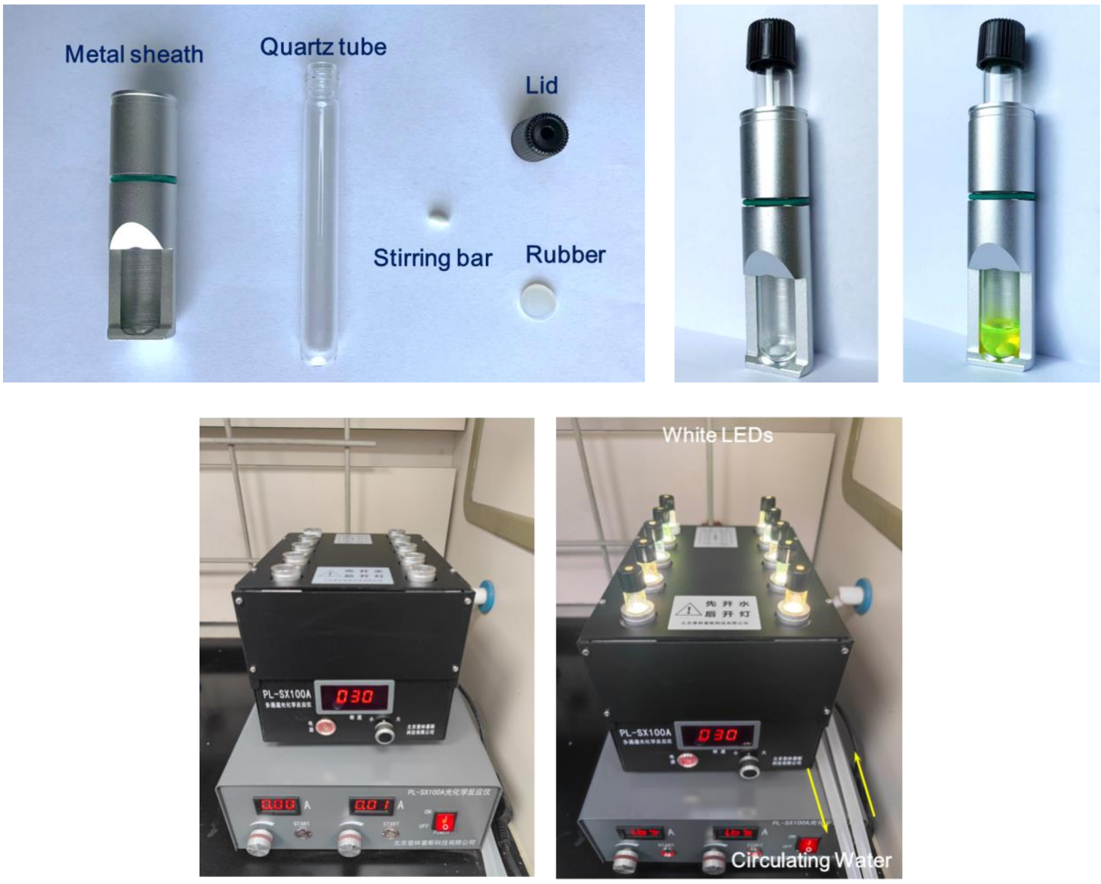

Supplementary Fig. 1. Photographs showcasing photocatalytic equipment. Photocatalysis setups designed and made by Beijing Precise Technology Co., Ltd. from China. The spectrum details of the employed light sources in our photocatalysis were provided in our reported work[1].

Comments: After charged with 1 mL of solvents, the quartz setup remains plenty of space, which is conducive to release the ethylene from the liquid phase to gas phase. Comparing with the e-shuttle[2], the p-shuttle is without electrode and N_2 gas flow during the whole photocatalysis process.

3. Detection of ethylene product in dibromination reaction

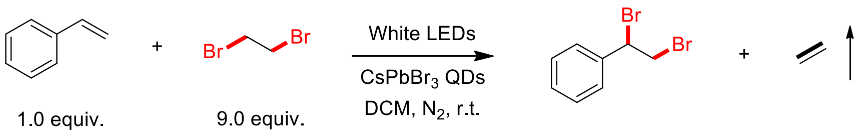

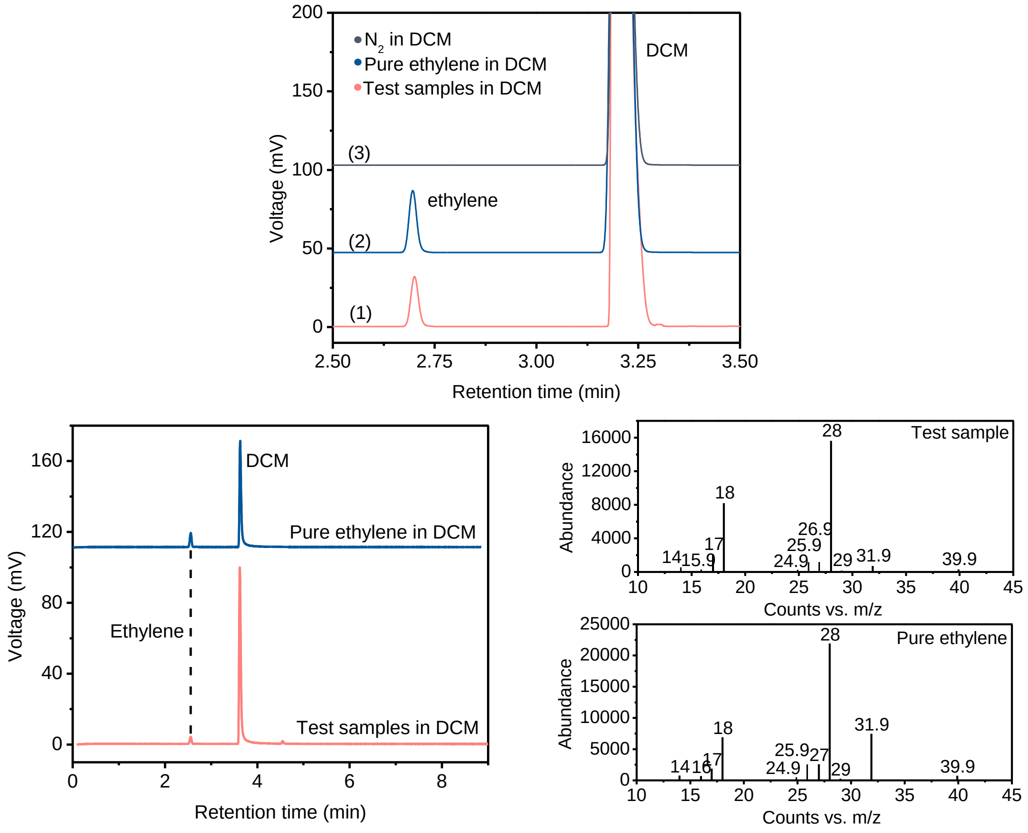

Supplementary Fig. 2. Detection of ethylene using GC and GC-MS. GC spectra of ethylene gas from vicinal dibrominations using 1,2-dibromoethane as the donor (top picture). GC spectra of the sample (1), pure ethylene gas bubbled through DCM solvent (2) and pure N_2 gas bubbled through DCM solvent (3). GC-MS spectra of pure ethylene gas (bottom pictures). Commercial ethylene gas was bubbled through the DCM solvent in a vial with syringe needle for about 1 min and then the gas in the vial was subjected to GC-MS analysis.

Reaction condition: styrene (1 mmol, 1.0 equiv.), 1,2-dibromoethane (9.0 mmol, 9.0 equiv.), $CsPbBr_3$ QDs (13 mg), and DCM (2 mL) under N_2 atmosphere using white LED illumination for 4 days. The gas components were detected by GC and GC-MS.

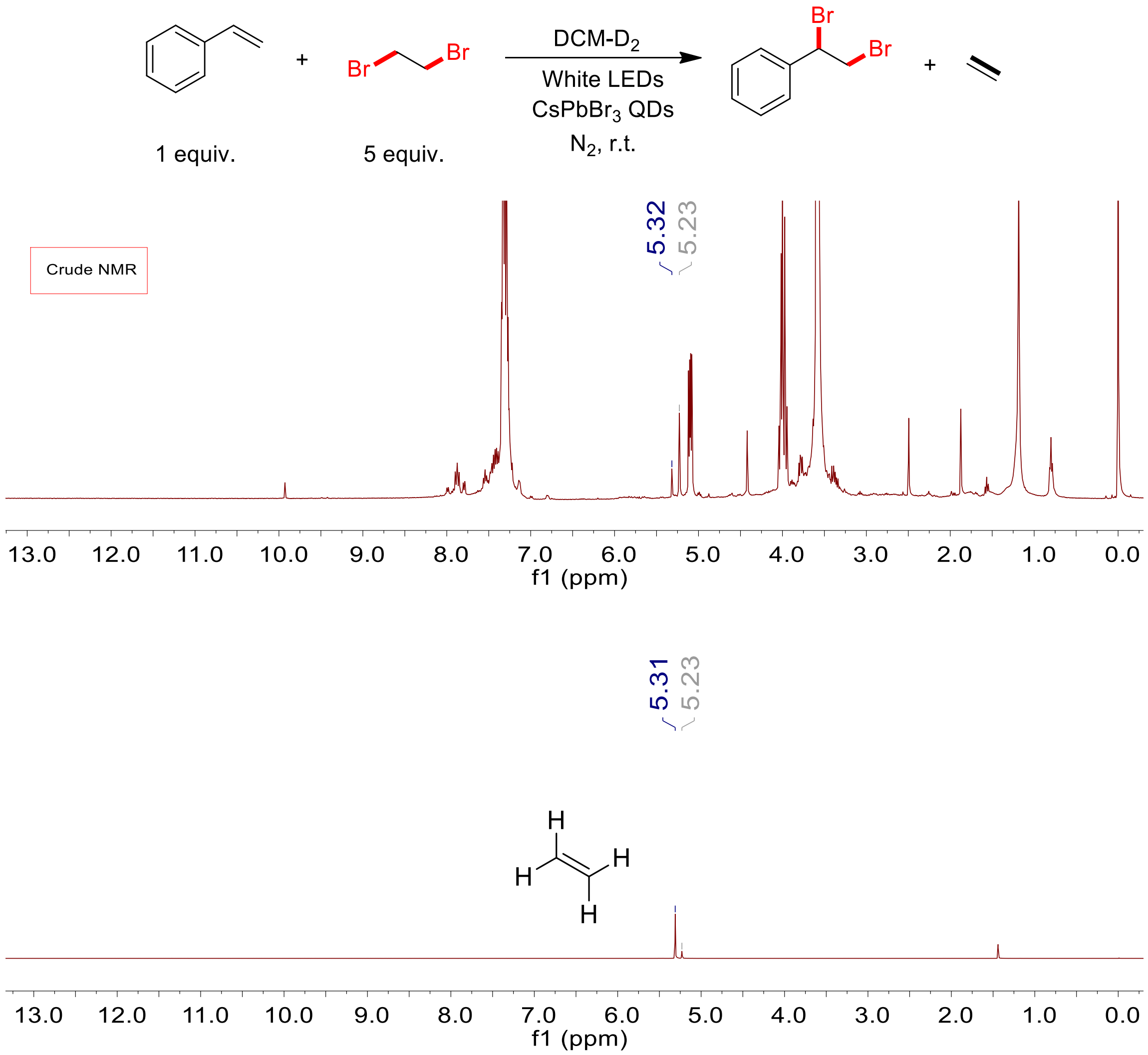

Supplementary Fig. 3. Detection of ethylene using NMR. Crude ^{1}H NMR of the dibromination reaction with 1,2-dibromoethane as the donor in DCM.

4. Characterization of QD catalysts after reaction

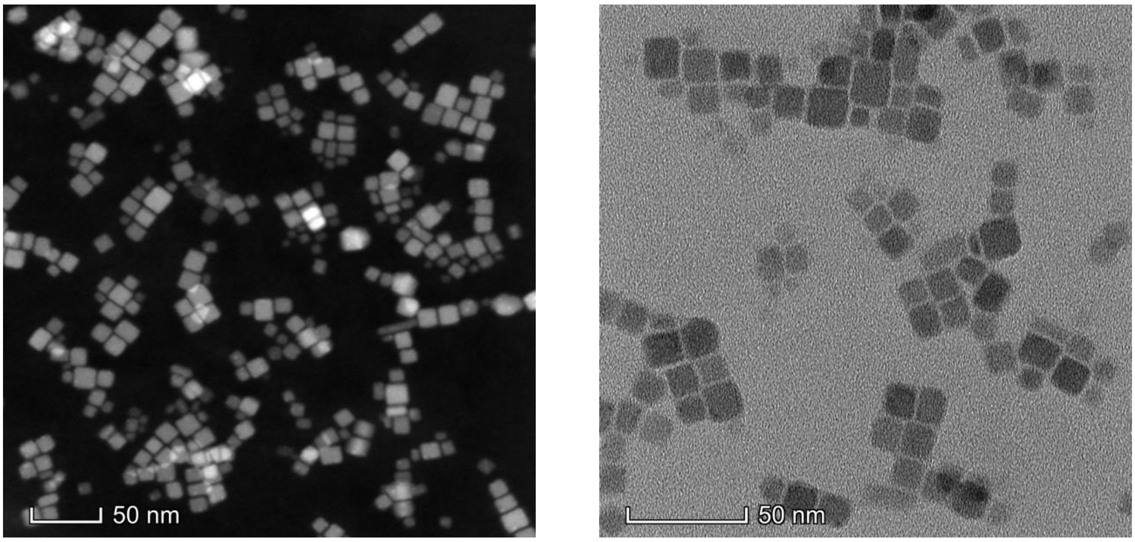

Supplementary Fig. 4. Characterization of QD catalysts after reaction. High resolution TEM images of $CsPbBr_3$ QDs after photocatalytic reaction.

5. Reversible vicinal dibromination experiments

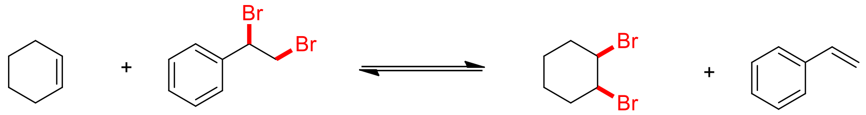

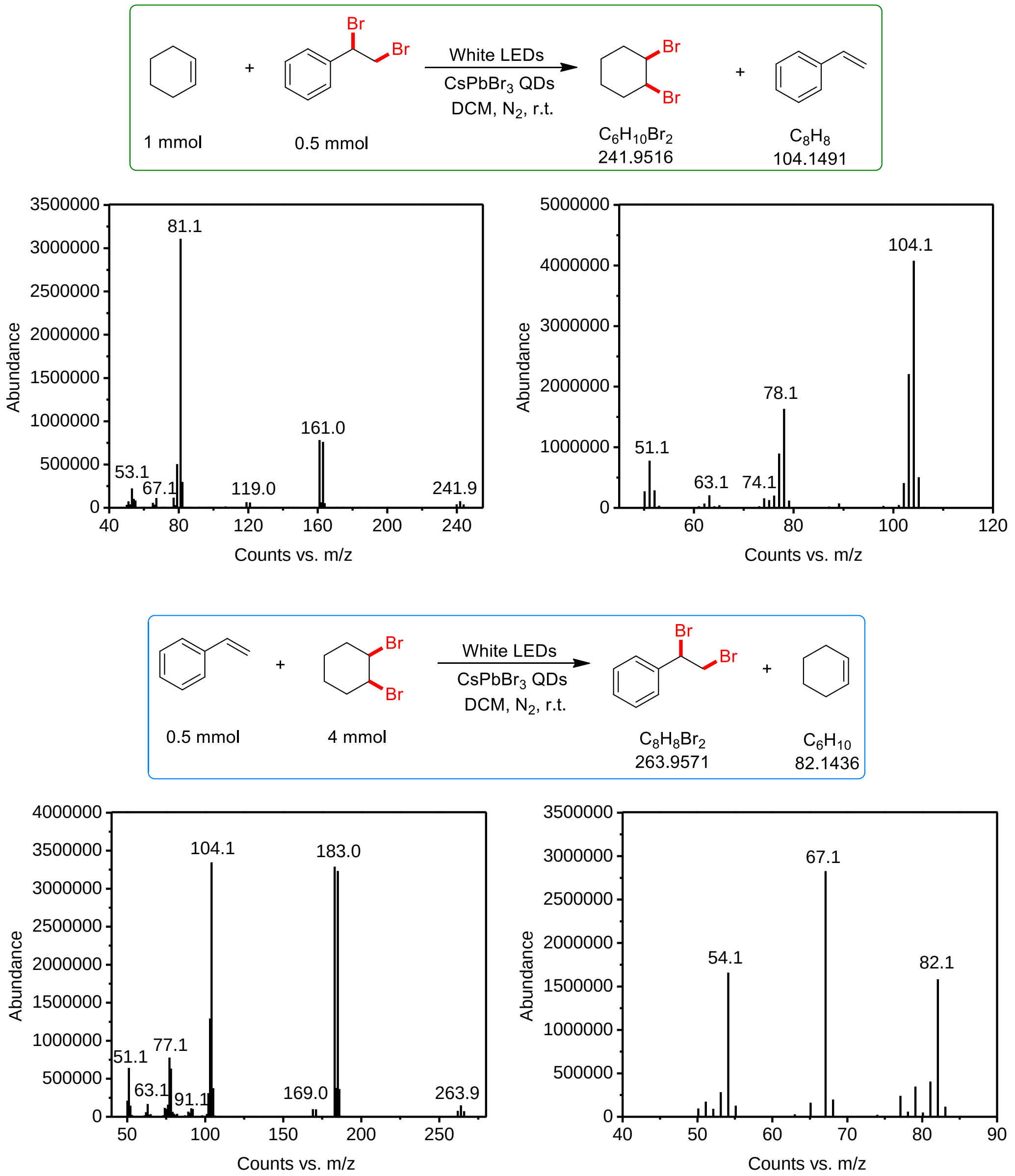

Supplementary Fig. 5. Evidence of reversibility. Reversible vicinal dibromination experiments with (1,2-dibromoethyl)benzene and 1,2-dibromocyclohexane as the donor.

6. Cost calculations

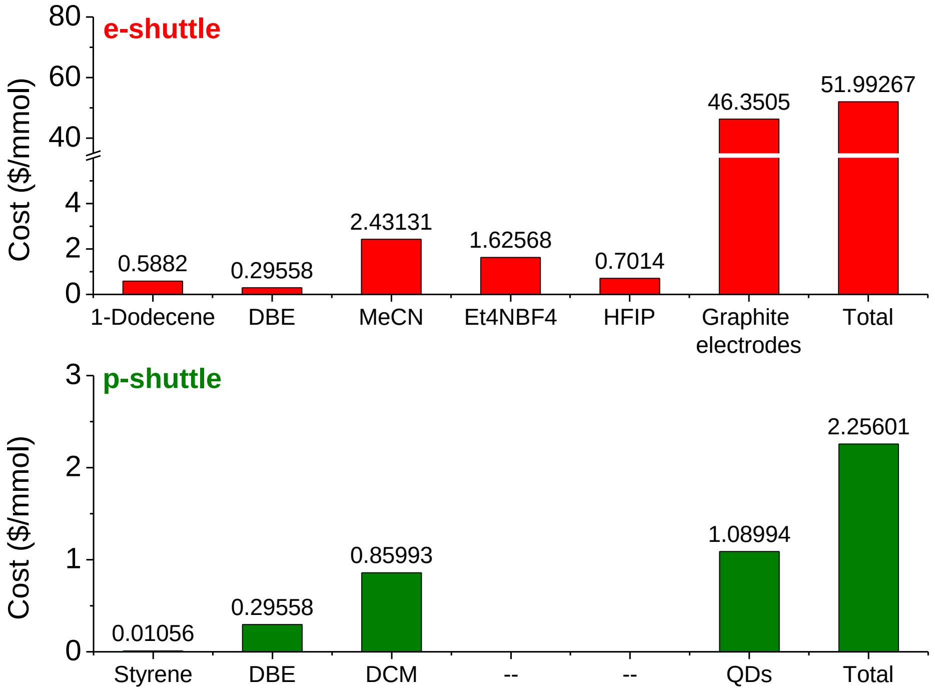

Supplementary Fig. 6. Price comparison. Cost calculations for the two dibromination methods (e-shuttle and p-shuttle).

Cost calculations for the QDs were conducted by considering commercially available precursors for the synthesis of the QDs, taking into account the isolated yield of the QDs after purification. The cost analysis included the precursors (Cs_2CO_3 and $PbBr_2$) and other necessary reagents (OA, OAm, and ODE) for QD synthesis, excluding the ethyl acetate and hexane solvents. The pricing information for all chemical reagents was sourced from Sigma-Aldrich, except for the pricing information of the graphite electrodes, which was obtained from IKA. Regarding the cost calculations for the graphite electrodes, commercially available graphite electrodes commonly used in electrochemistry for the preparation of 1 mmol target product were considered, instead of raw graphite material. This ensures a more realistic estimation of the costs associated with the use of graphite electrodes in the experimental setup.

7. Survey of kinetic process for the dibromination

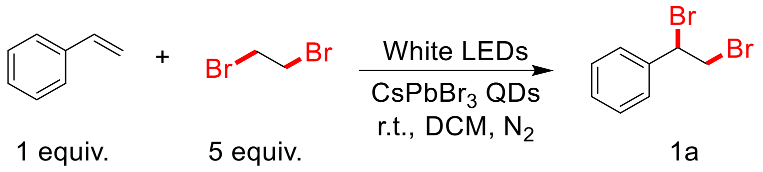

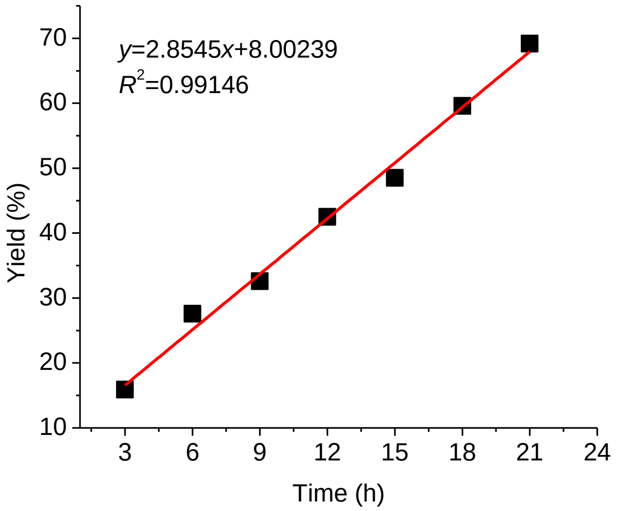

Supplementary Fig. 7. Kinetic study. Kinetic data for the dibromination reation using $CsPbBr_3$ QDs as catalysts.

8. Ex-situ PL spectra of $CsPbBr_3$ QDs in DCM and DCM+DBE

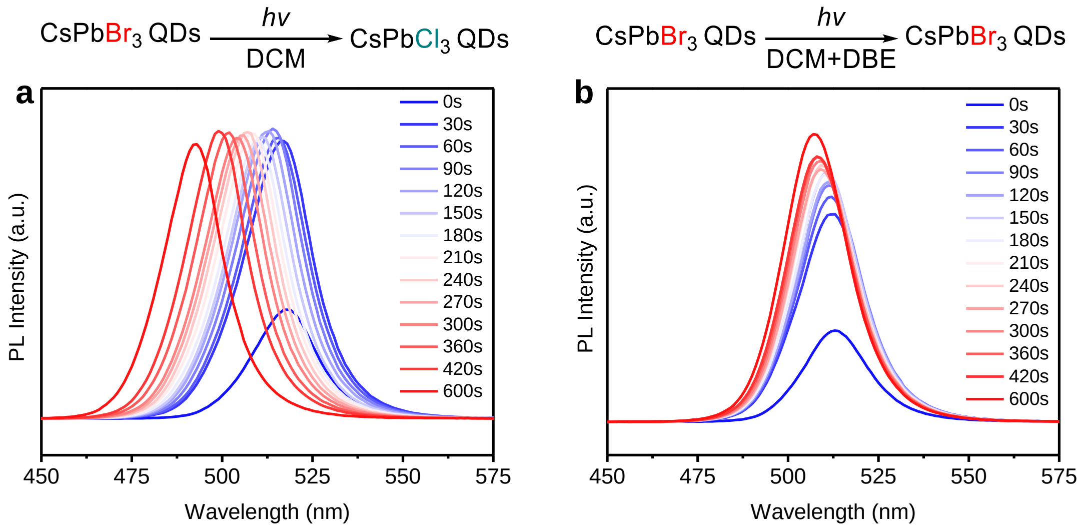

Supplementary Fig. 8. PL spectroscopic characterization. a,b, Ex-situ PL spectra of $CsPbBr_3$ QDs in DCM (**a**) and DCM+DBE (**b**).

Experimental Procedures[1,3]: A home built optical quartz setup charged with $CsPbBr_3$ QDs, 32.5 μL of OAm, 0.5 mL of hexane and 1 mL of DCM was irradiated using a xenon lamp equipped with a 420 nm cutoff filter (powder intensity ~120 mW/cm^2, PLS-SXE300/300UV from Beijing Perfectlight Technology Co., Ltd.). The illumination reactions were carried out under ambient conditions without stirring. Upon irradiation, PL spectra of $CsPbBr_3$ QDs were collected directly from the colloidal suspension at different times (see left one). Another QD sample with 0.2 mL of DBE was irradiated under the same conditions and the emission spectra were collected (right one).

Principle: The emission wavelength of excitonic PL in $CsPbX_3$ perovskite QDs is strongly influenced by their chemical composition. The $CsPbCl_3$ QDs exhibit an intrinsic exciton PL peak at approximately 410 nm, while the $CsPbBr_3$ QDs display an intrinsic exciton PL peak at around 520 nm. On the other hand, the $CsPbBr_xCl_{3-x}$ QDs exhibit intrinsic exciton PL across a wavelength range spanning from 410 to 520 nm. When halide exchange occurs on the surface of QDs, a continuous shift in the PL wavelength can be observed.

Comments: Because the photoinduced anion-exchange takes place between $CsPbBr_3$ and DCM, continuous change of emission spectra (an approximate 25 nm blue-shift) was observed when $CsPbBr_3$ QDs were dispersed in a mixture of DCM and hexane. However, the addition of DBE, under the same conditions, led to a slight blue-shift (6 nm) of the emission wavelength. The competitive anion-exchange occurred on the $CsPbBr_3$ QD surface because DCM and DBE both extracted photo-generated electron from the QDs and produced the corresponding halide anions (Cl^- and Br^-)[1] in the reaction process.

9. Radical trapping experiments for the dibromination reaction

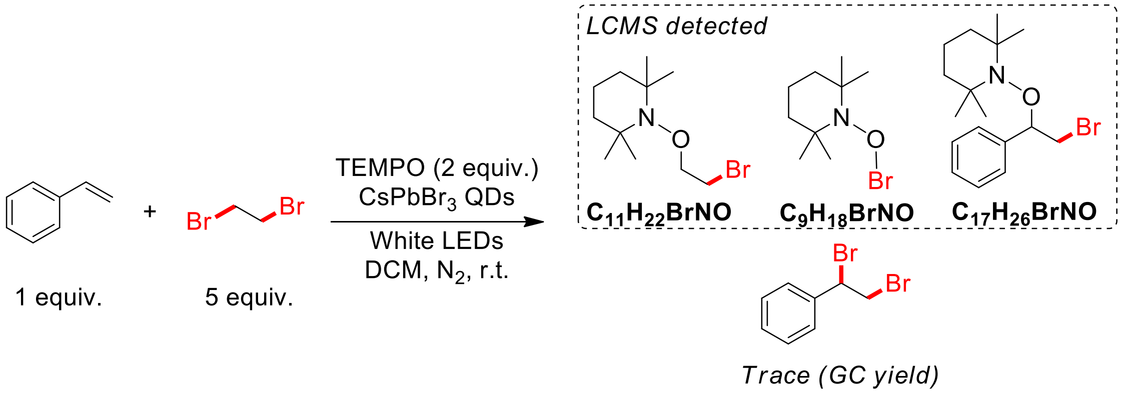

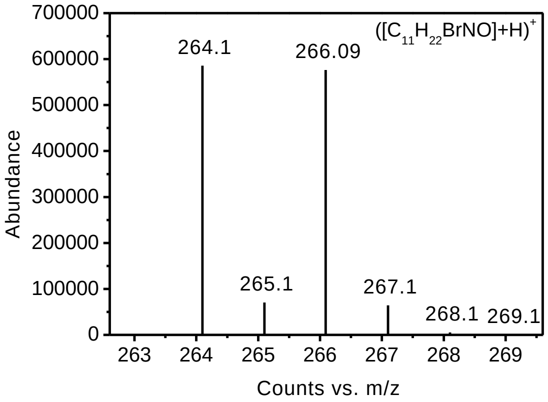

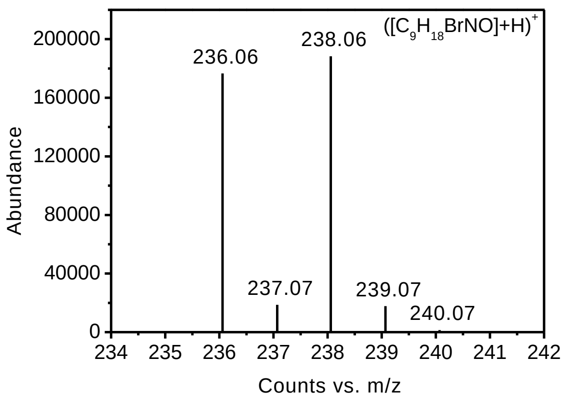

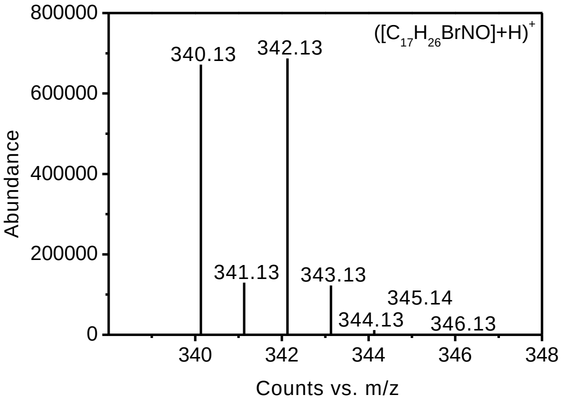

Supplementary Fig. 9. **TEMPO trapping study.** Radical trapping experiment for the dibromination reaction with 1,2-dibromoethane as the donor.

10. ATRA reaction mechanism

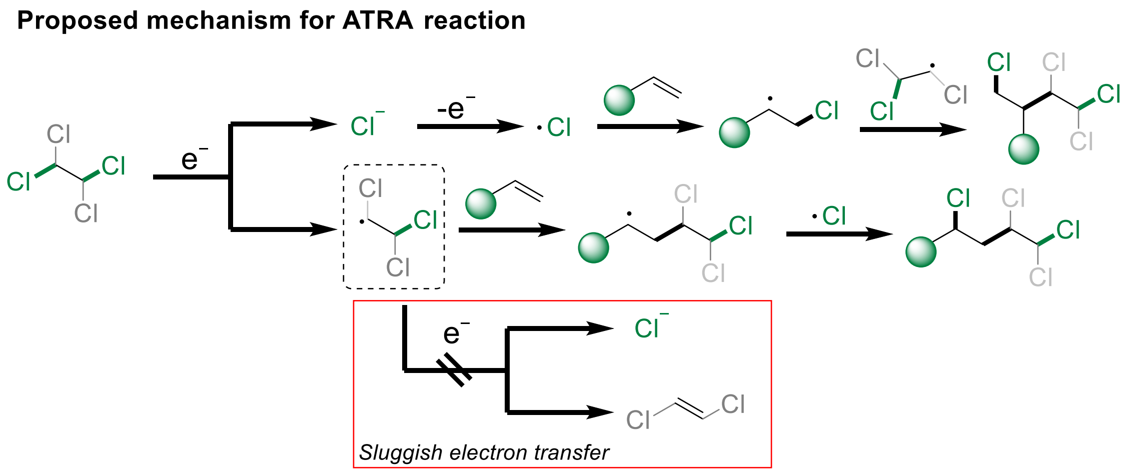

Supplementary Fig. 10. The mechanism of atom transfer radical addition (ATRA) reaction. The two-electron reduction of the TCE donor is the key route to break two C−Cl bonds, otherwise two ATRA byproducts will be obtained.

11. Optimized Cu loading in dichlorination

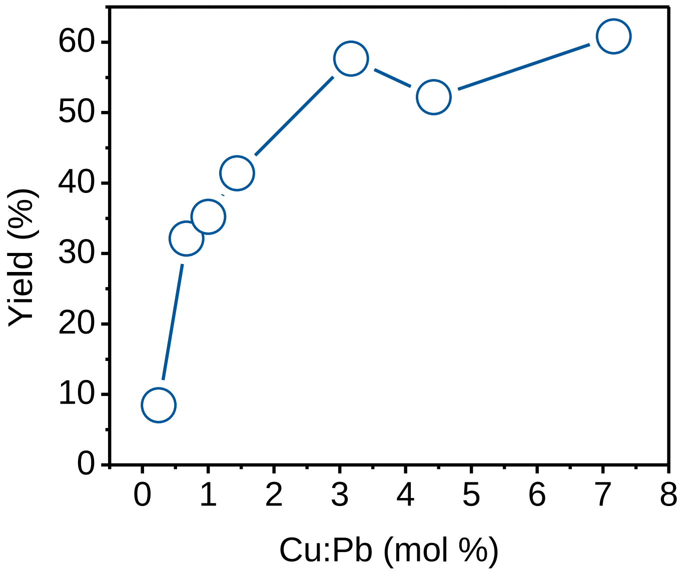

Supplementary Fig. 11. Optimized Cu loading for yield of (1,2-dichloroethyl)benzene. The QDs with different Cu loading were synthesized by ion-exchange (1.2 mg $CuBr_2$ in 20 mg QDs) under different time.

12. Characterization of Cu-$CsPbBr_3$ QDs

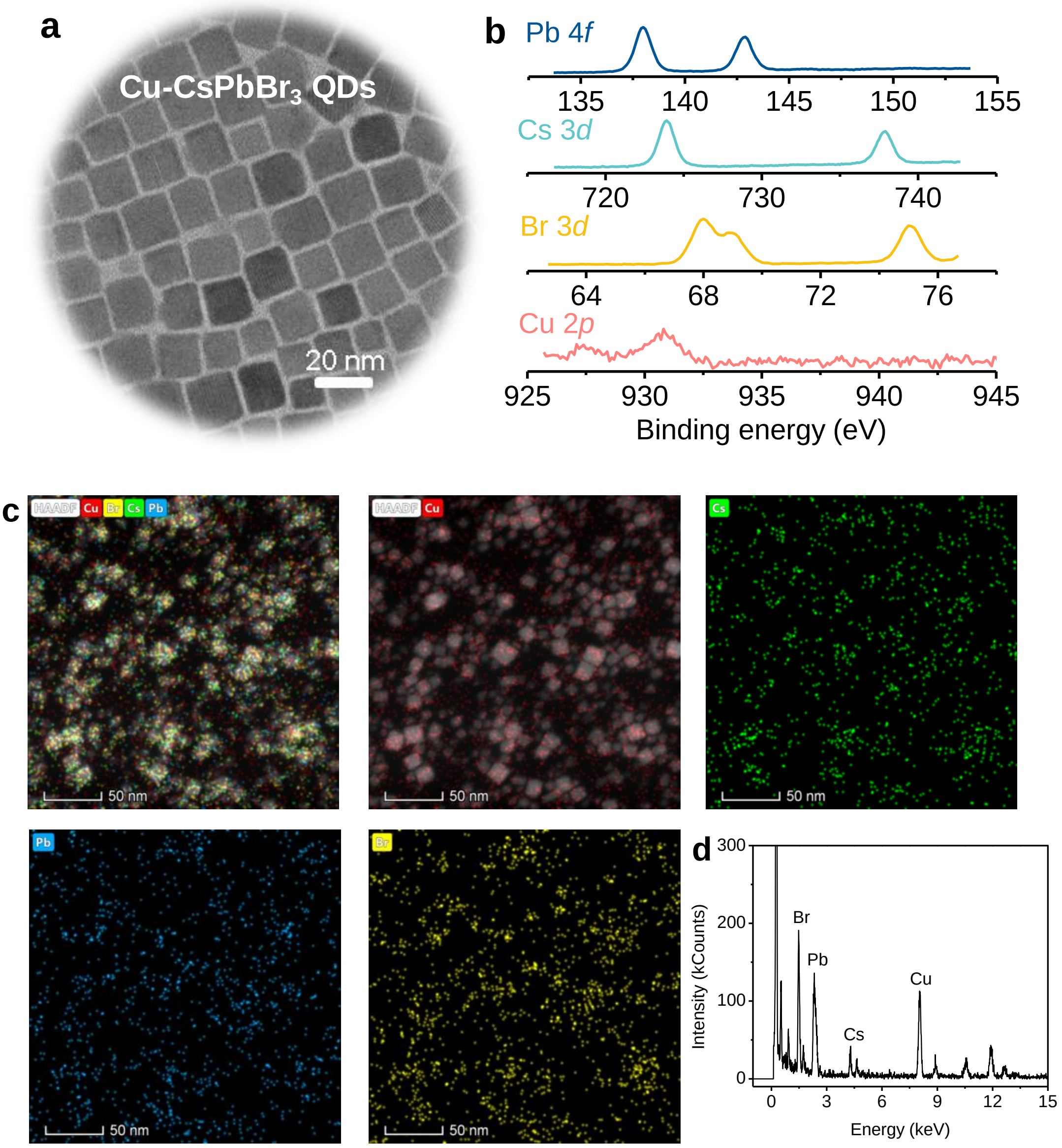

Supplementary Fig. 12. Characterization of Cu-$CsPbBr_3$ QDs. a, TEM image of Cu-$CsPbBr_3$ QDs. **b,** XPS spectra of Cu-$CsPbBr_3$ QDs. **c,** HAADF-STEM image and the corresponding EDS element mapping of Cu, Cs, Pb, and Br. **d,** the corresponding EDS energy peaks of Br, Pb, Cs, and Cu. Note that microporous molybdenum TEM grids were used as TEM substrate in the elemental analysis.

13. Crude ^{1}H NMR of the dichlorination reaction

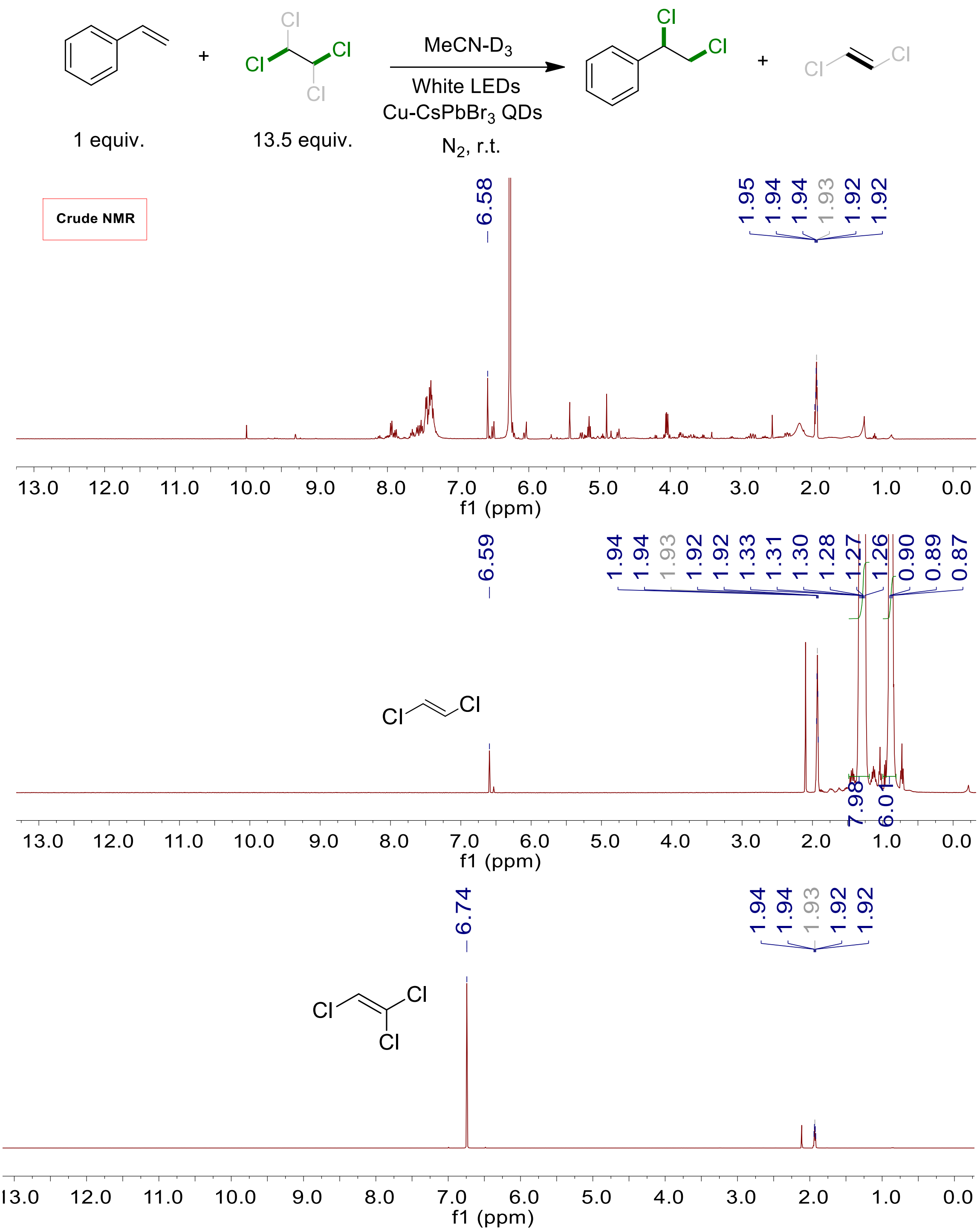

Supplementary Fig. 13. Detection of 1,2-dichloroethylene using NMR. Crude ^{1}H NMR of the dichlorination reaction with 1,1,2,2-tetrachloroethane as the donor. Note that commercial 1,2-dichloroethylene is dissolved in N-hexane.

14. Radical trapping experiment for the dichlorination reaction

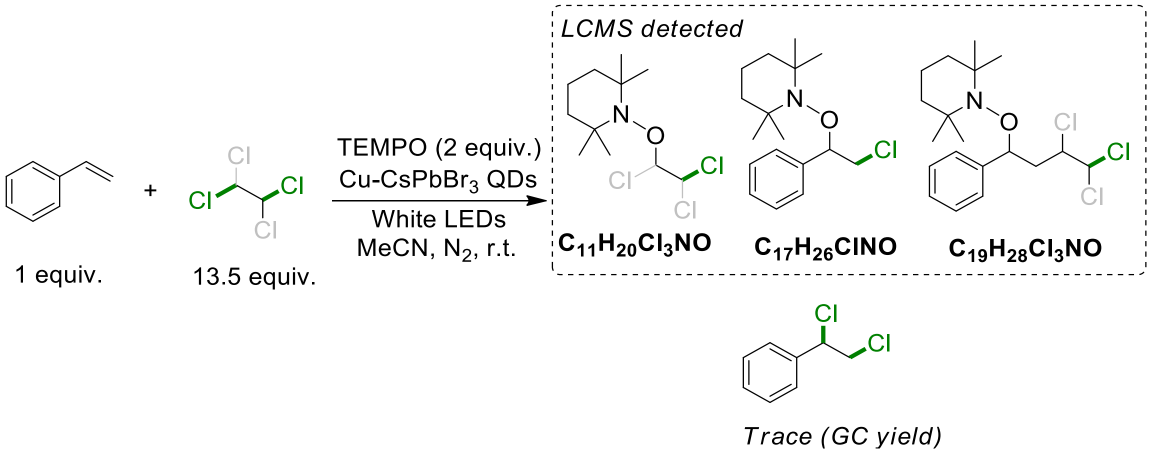

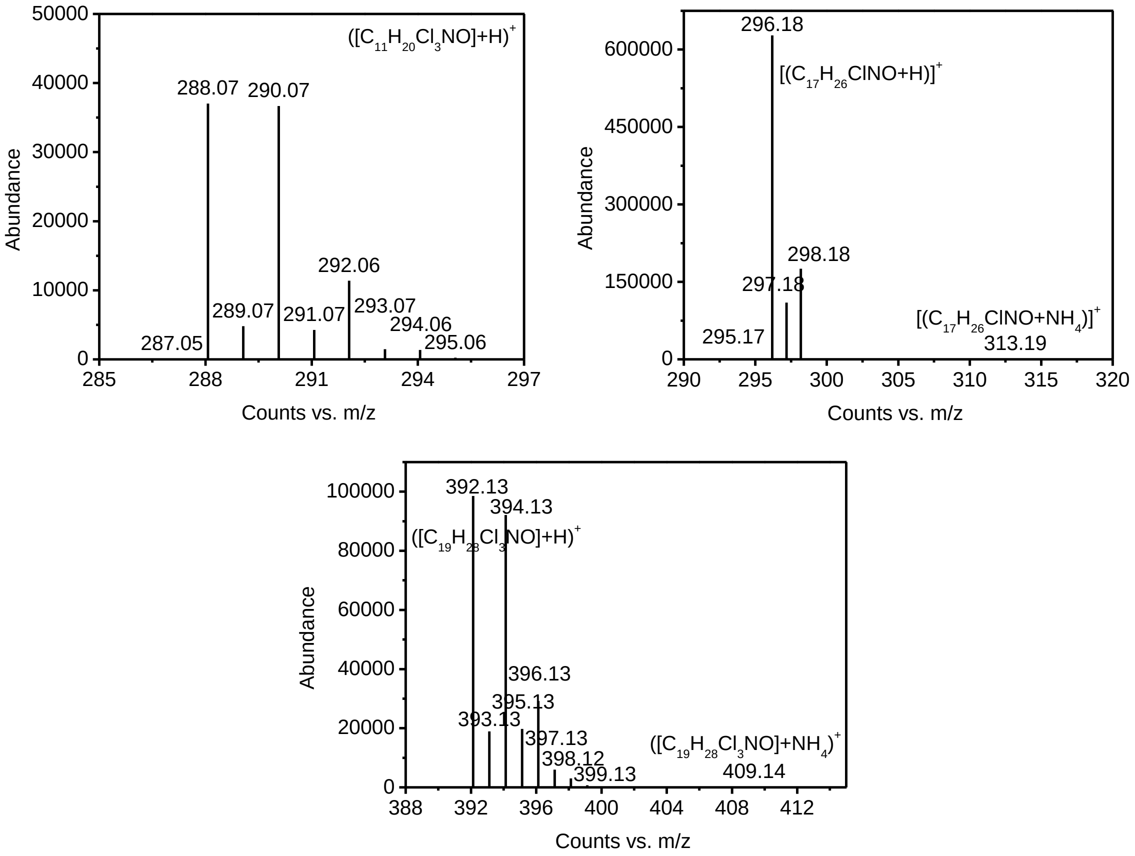

Supplementary Fig. 14. TEMPO trapping study. Radical trapping experiment for the dichlorination reaction with 1,1,2,2-tetrachloroethane as the donor.

15. GC graphs of hetero-dihalogenation mixture

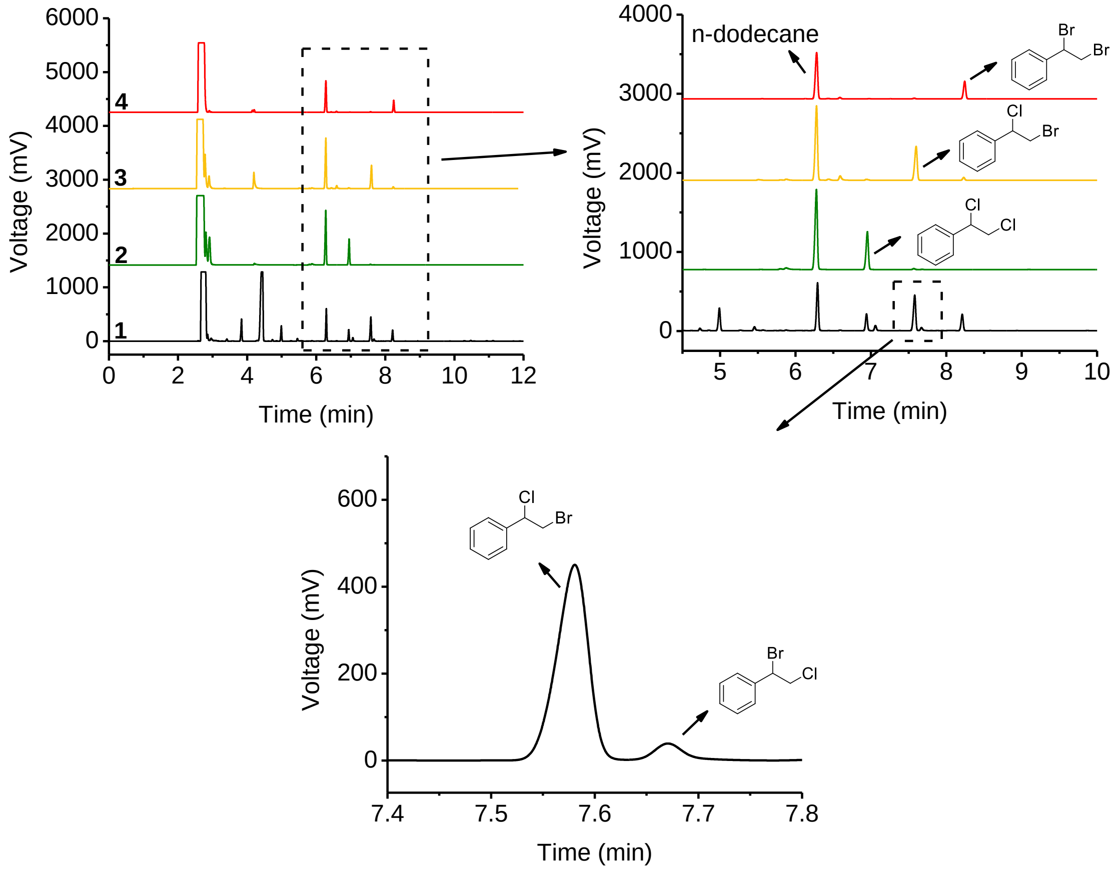

Supplementary Fig. 15. Detection of hetero-dihalogenation using GC. GC graphs of hetero-dihalogenation mixture using Cu-$CsPbBr_3$ QDs as catalysts (1), pure (1,2-dichloroethyl)benzene (2), pure (2-bromo-1-chloroethyl)benzene (3), and pure (1,2-dibromoethyl)benzene (4).

16. Radical trapping experiment for the hetero-dihalogenation

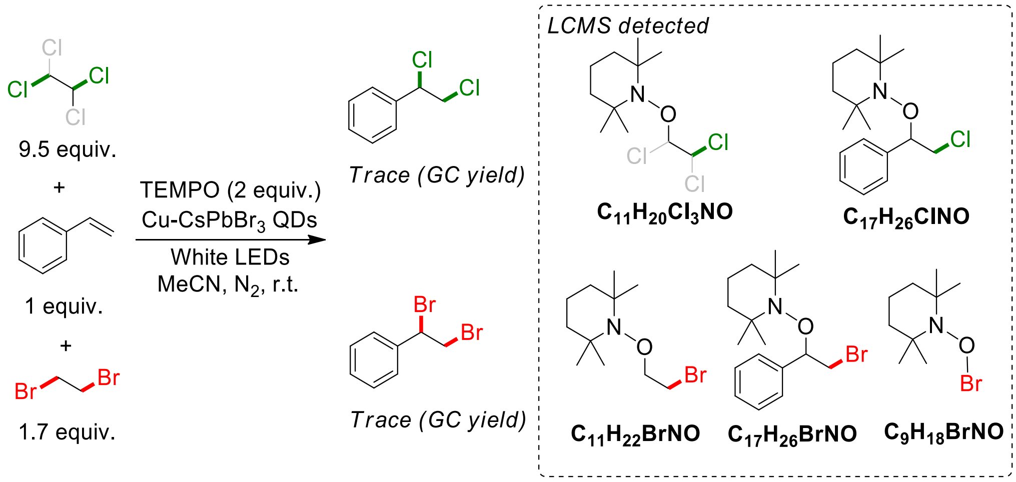

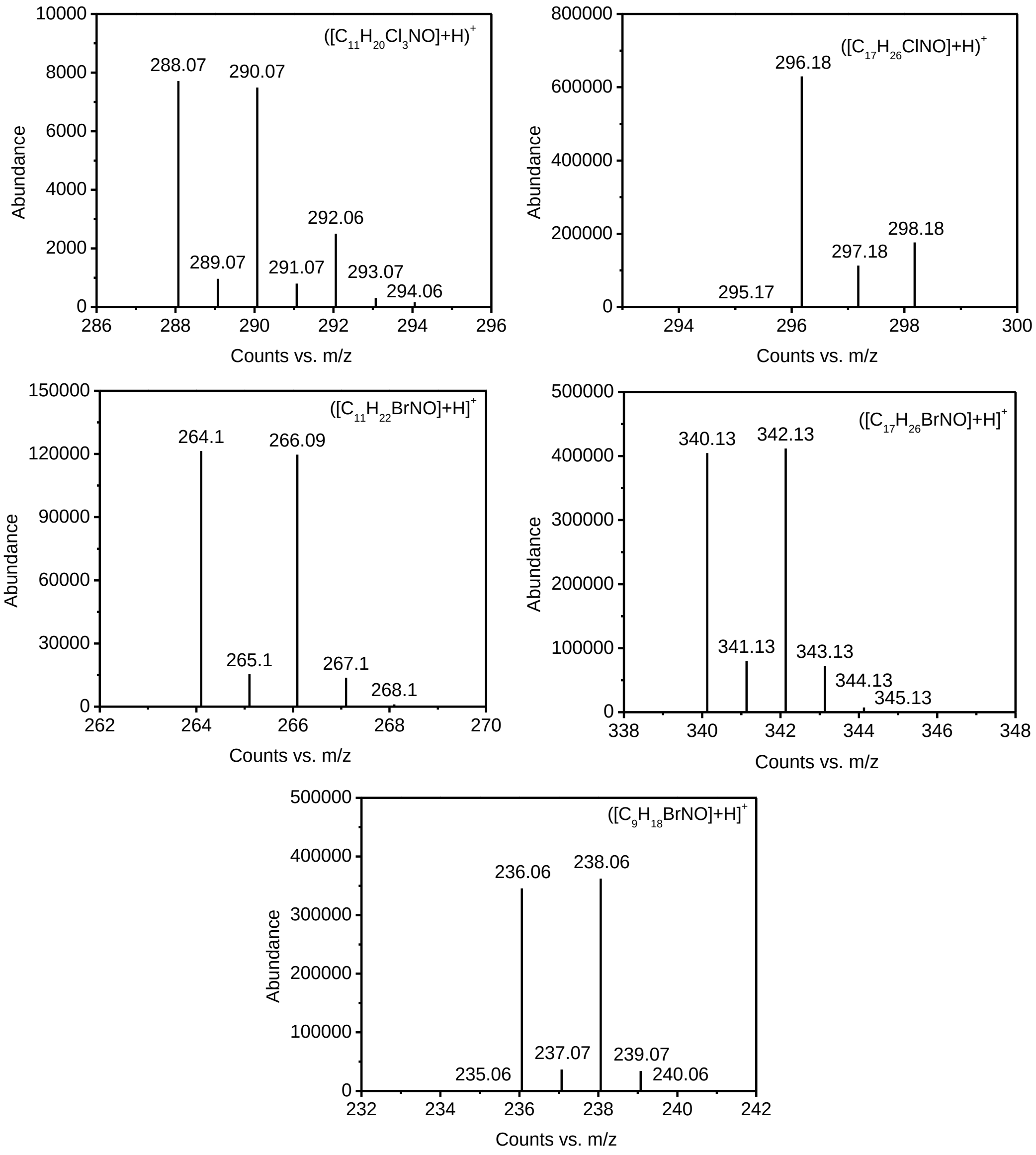

Supplementary Fig. 16. TEMPO trapping study. Radical trapping experiment for the hetero-dihalogenation reaction with 1,2-dibromoethane and 1,1,2,2-tetrachloroethane as the donor.

17. Ex-situ PL spectra of Cu-QDs in TCE and TCE+DBE.

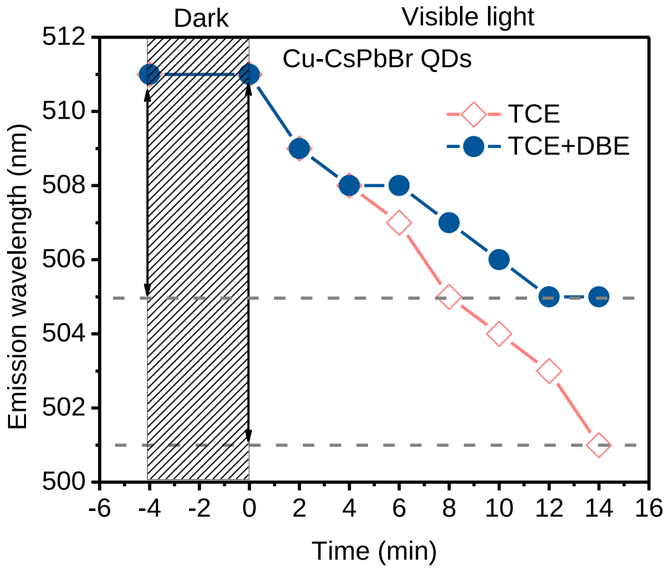

Supplementary Fig. 17. PL spectroscopic characterization. Ex-situ PL spectra of Cu-$CsPbBr_3$ QDs in TCE and TCE+DBE.

1 mg Cu-$CsPbBr_3$ QDs, 20 μL of OAm, 2 mL of hexane and 0.2 mL of TCE (**Sample A**)
1 mg Cu-$CsPbBr_3$ QDs, 20 μL of OAm, 2 mL of hexane, 0.2 mL of TCE and 0.03 mL of DBE (**Sample B**)

A home built optical quartz setup charged with **Sample A** and **B** were respectively irradiated under ambient conditions without stirring using a xenon lamp equipped with a 420 nm cutoff filter (powder intensity ~100 mW/cm^2). Photoluminescence spectra of Cu-$CsPbBr_3$ QDs were collected directly from the colloidal suspension at different illumination conditions times[1,3].

18. Crude ^{1}H NMR of the hetero-dihalogenation and dibromination reaction

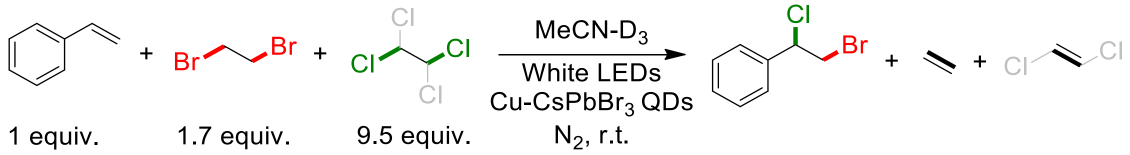

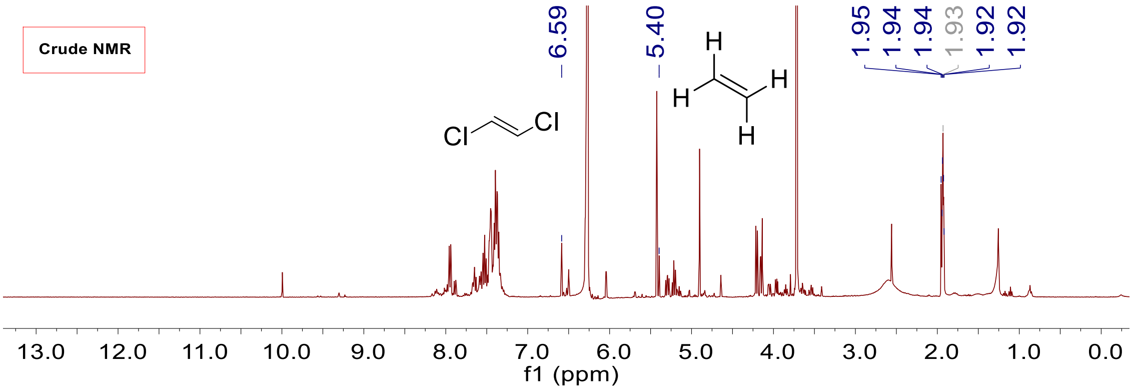

Supplementary Fig. 18. Detection of ethylene and 1,2-dichloroethylene using NMR. Crude ^{1}H NMR of the hetero-dihalogenation reaction with 1,2-dibromoethane and 1,1,2,2-tetrachloroethane as the donor.

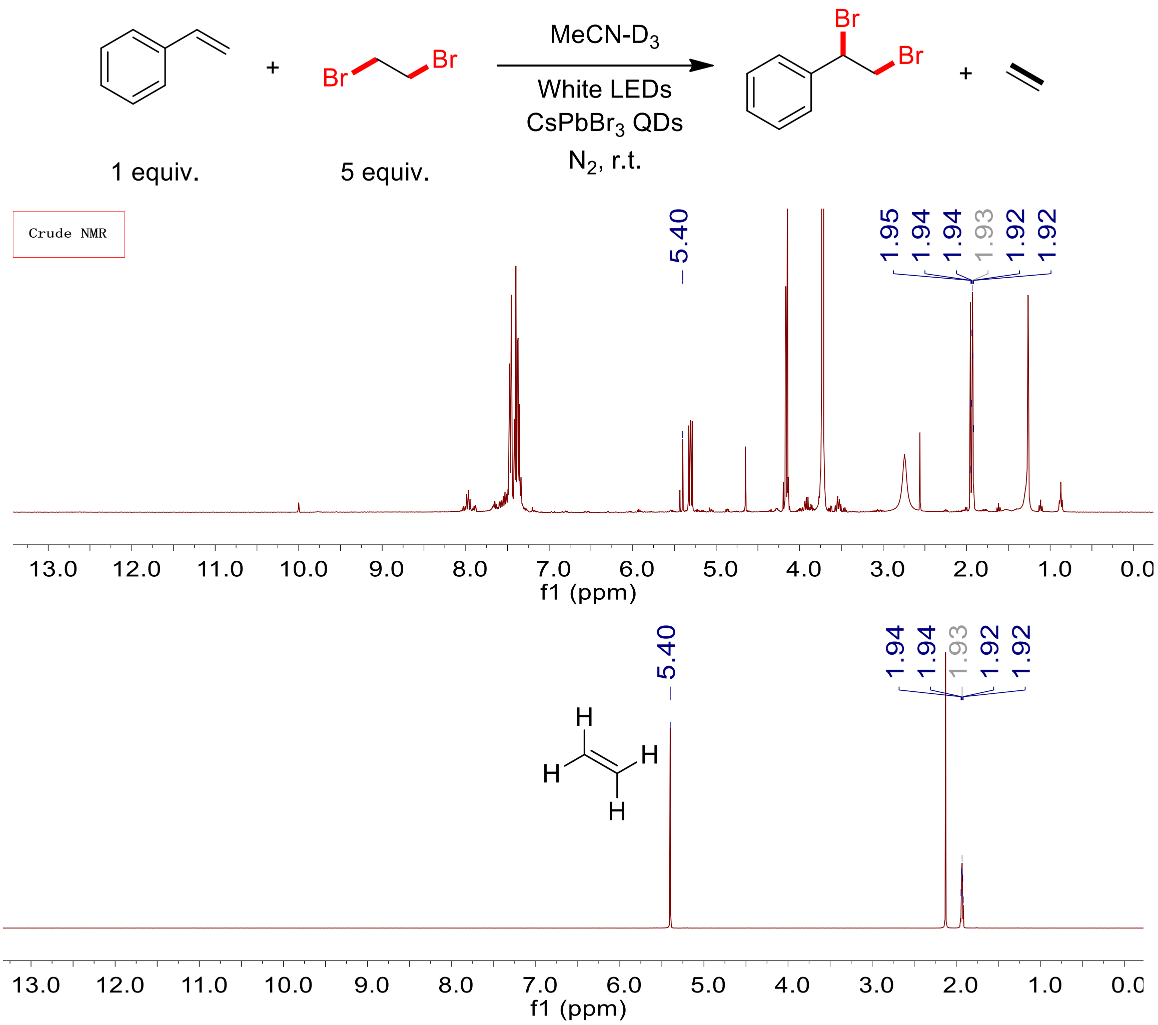

Supplementary Fig. 19. Detection of ethylene using NMR. Crude ^{1}H NMR of the dibromination reaction with 1,2-dibromoethane as the donor in MeCN.

19. Determining turnover number (TON) and apparent quantum yield (AQY)

TON was calculated according to the Supplementary Equation (1)[4]:

$$TON = \frac{Moles\ of\ desired\ product}{Moles\ of\ catalyst\ QD} \quad (1)$$

Dibromination reaction: The number of moles of the catalyst QDs was determined by considering factors such as the elemental composition mass, size, mass density, and molecular formula ($CsPbBr_3$) of the catalyst QDs. A single $CsPbBr_3$ QD with a size of 9.8±0.6 nm was considered as the effective catalyst unit for photoredox reactions. Through inductively coupled plasma optical emission spectroscopy (ICP-OES) analysis, it was found that the catalyst QDs contained 0.47 mg of elemental Pb. The mass density of the QDs was approximately equal to that of bulk material[5], approximately 4.86 g/cm^3. Therefore, the number of moles of $CsPbBr_3$ catalyst QDs was calculated to be 4.5×10^{-4} μmol, taking into account the molecular weight of 579.81.

The $CsPbBr_3$ QDs was reused via centrifugation from the reaction mixture (0.1 mmol scale) and was reapplied at the same scale with the sbstrate. The QDs were active for at least three cycles with the yield noted as 63%, 51% and 41%. The catalytic turnover number based on QD moles was calculated according to the recentrifuge method.

$$TON_{Br} = \frac{0.1\ mmol \times (63\% + 51\% + 41\%)}{4.5 \times\ 10^{-4}\ \mu mol} = 342{,}222$$

Dichlorination reaction: ICP-OES results showed a Cu to Pb ratio of 5.3:100, where 0.51 mg of elemental Pb in the Cu doped QDs. Therefore, the moles of Cu-$CsPbBr_3$ QDs were equal to 4.9×10^{-4} μmol, considering the above calculations.

The Cu-$CsPbBr_3$ QDs was used for the dichlorination reaction (0.1 mmol scale) and the yield was 60 %. The catalytic turnover number based on Cu-QD moles was calculated according to the formula.

$$TON_{Cl} = \frac{0.1\ mmol \times 60\ \%}{4.9 \times\ 10^{-4}\ \mu mol} = 122{,}449$$

AQY was calculated according to the Supplementary Equation (2)[6]:

$$AQY = \frac{Moles\ of\ desired\ product\ per\ second}{Moles\ of\ photons\ per\ second} = \frac{n}{q} \times 100\% \quad (2)$$

The photon flux (moles of photons per second) was calculated by the Supplementary Equation (3):

$$q = \frac{I \times A \times \lambda}{hc\ \times (6.02 \times 10^{23})} \quad (3)$$

Here, the power of light illumination by the reaction mixture is represented by I. In this study, the intensity of blue LED light was measured using a photodiode at the location where the reaction vial was positioned, with a value of I = 0.015 W/cm^2. A denotes the illumination area, which was approximately 1.8 cm^2. The wavelength of the LED source, represented by λ, was 455 nm, equivalent to 4.55×10^{-7} m. Planck's constant (h) is measured in J-s, and the speed of light (c) is given in meters per second.

$$q = \frac{I \times A \times \lambda}{hc\ \times (6.02 \times 10^{23})} = \frac{0.015 \times 1.8 \times 4.55 \times 10^{-7}}{6.62 \times 10^{-34} \times 3 \times 10^{8} \times 6.02 \times 10^{23}}$$
$$= 1.02 \times 10^{-7}\ mol/s$$

The moles of product per second (n) was determined using yield of product formed divided by reaction time (t) in seconds, here using the 0.1 mmol scale reaction, yield 55 %, t=8 hours.

$$n = \frac{0.1 \times 10^{-3}\ mol \times 0.55}{8 \times 3600\ s} = 1.91 \times 10^{-9}\ mol/s$$

Therefore the overall AQY

$$AQY_{Br}\ = \frac{n}{q} \times 100\ \% = \frac{1.91 \times 10^{-9}\ mol/s}{1.02 \times 10^{-7}\ mol/s} \times 100\ \% = 1.8\ \%$$

Note that ever one mole of dibromination product requires two mole of electron as demonstrated by our proposed mechanism.

$$QY_{Br} = 2 \times AQY = 2 \times 1.8\ \% = 3.6\ \%$$

20. Optimization experiments

Supplementary Table 2. Control experiments for the synthesis of 1a.[a]

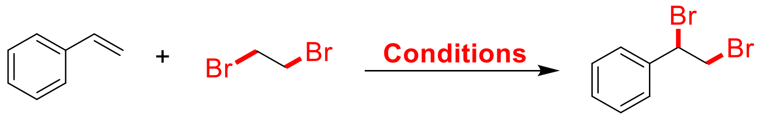

Entry	Photocatalysts (amount)	Light	GC Yield [%]
1	$CsPbBr_3$ QDs (4.0 mg)	√	57
2[b]	$CsPbBr_3$ QDs (4.0 mg)	×	Trace
3	–	√	0
4	$PbBr_2$ (5.0 mg)	√	0
5	Cs_2CO_3 (5.0 mg)	√	0
6	$PbBr_2$ and Cs_2CO_3 (10.0 mg)	√	0

[a]Reaction conditions: styrene (0.1 mmol, 1.0 equiv.), 1,2-dibromoethane (1 mmol, 10 equiv.) and DCM (1 mL) under N_2 atmosphere using white LED illumination at ~25 ℃ for 48 h. [b]Reaction performed in dark conditions at 25 ℃.

Supplementary Table 3. Survey of photocatalysts[a].

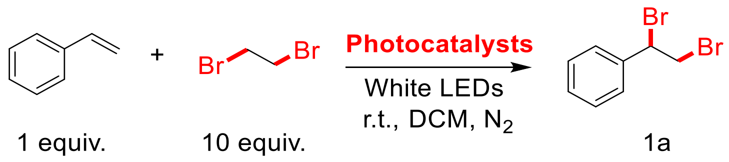

Entry	Photocatalysts (amounts)	GC Yield [%]
1	$Ir(ppy)_3$ (5 mol%)	0
2	$Ru(bpy)_3(PF_6)_2$ (5 mol%)	0
3	4CzIPN (10 mol%)	0
4	TiO_2 (10 mg)	0
5	SiC (10 mg)	Trace
6	ZnS (10 mg)	0
7	g-C_3N_4 (10 mg)	0
8	CdSe/ZnS QDs (10 mg)[b]	2
9	CdS/ZnS QDs (10 mg)[b]	3
10	$CsPbBr_3$ QDs (4 mg)	58[c]

[a]Reaction conditions: styrene (0.1 mmol, 1.0 equiv.), 1,2-dibromoethane (1 mmol, 10 equiv.), dichloromethane (1 mL) under white LED illumination at about 25 ℃ for 48 h., [c]illumination time for 36 h. [b]Cd-based QDs were bought from Beijing Beida Jubang Science & Technology Co., Ltd.

Supplementary Table 4. Survey of bromide donors.

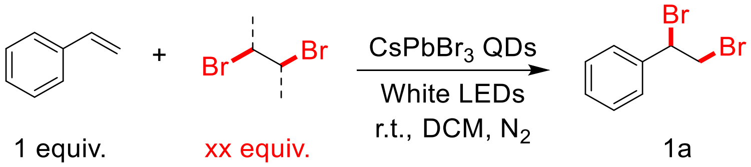

Entry	Bromide	Equivalents	GC Yield [%]
1	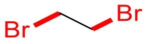	10	58
2	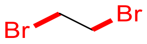	7.5	61
3	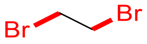	5	65
4	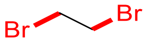	0	0
5	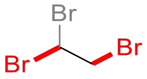	10	80
6	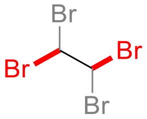	10	75

Supplementary Table 5. Survey of excitation wavelengths.

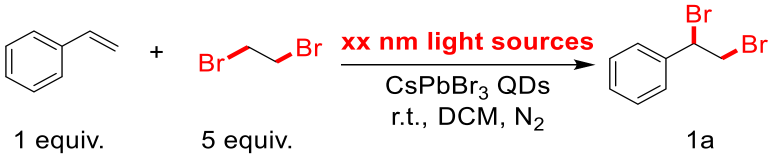

Entry	Wavelength [nm]	GC Yield [%]
1	380	23
2	420	24
3	455	55
4	500	30
5	590	0

Note: Various wavelengths of light source exhibit different power intensity.

Supplementary Table 6. Survey of reaction times.

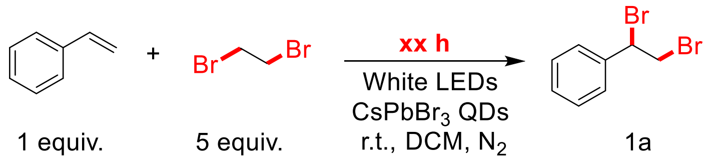

Entry	Reaction time [h]	GC Yield [%]
1	6	27
2	12	42
3	24	63
4	36	60
5	48	62

Supplementary Table 7. Survey of catalyst loadings.

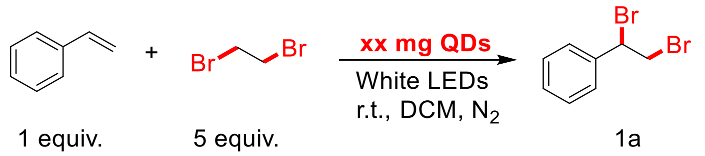

Entry	Catalyst loading [mg]	GC Yield [%]
1	0	0
2	1	65
3	2	59
4	4	52

Supplementary Table 8. Survey of solvent volumes.

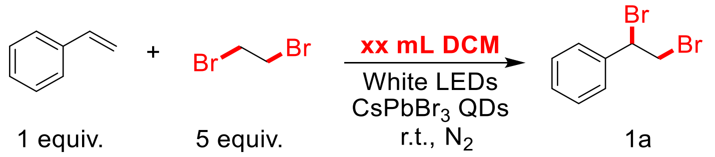

Entry	Solvent volume [mL]	GC Yield [%]
1	0.5	37
2	1	58
3	2	57
4	4	64
5	8	60

Supplementary Table 9. Initial results for the synthesis of 1b.[a]

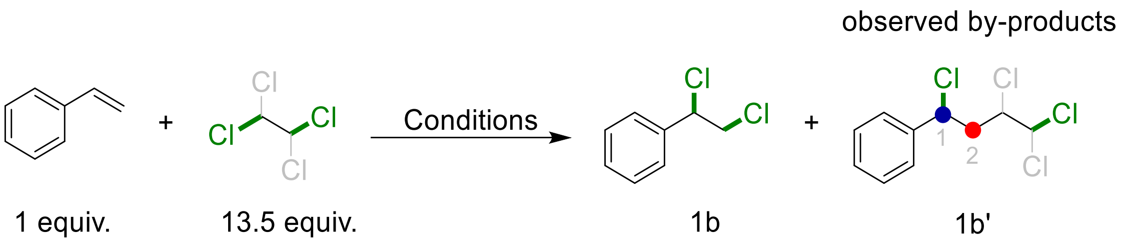

Entry	Catalysts	Yield [%][b]
1	$CsPbBr_3$ QDs	5
2	$CuCl_2$+$CsPbBr_3$ QDs	32

[a]Reaction conditions: styrene (0.1 mmol, 1.0 equiv.), 1,1,2,2-tetrachloroethane (1.35 m mol, 13.5 equiv.), $CuCl_2$ (1 mg), $CsPbBr_3$ QDs (4 mg), and MeCN (1 mL) under white LED illumination at ~25 ℃. [b]Yield of 1b determined by GC. Note two products 1b' (the sites 1 and 2 are interchangeable) were observed by GC-MS.

Supplementary Table 10. Survey of transition metal catalyst.

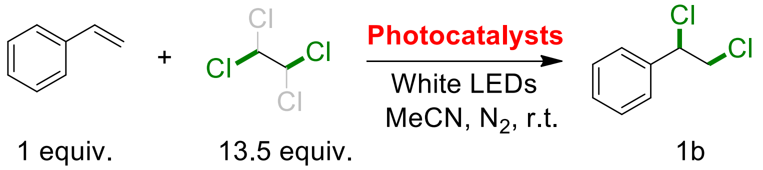

Entry	Photocatalysts	Metal precursors	Yield [%]
1	Cu(II)-$CsPbBr_3$	$CuBr_2$	51
2	Cu(I)-$CsPbBr_3$	CuBr	40
3	Fe(III)-$CsPbBr_3$	$FeCl_3\cdot 6H_2O$	7
4	Ni(II)-$CsPbBr_3$	$NiCl_2\cdot 6H_2O$	3
5	Ce(III)-$CsPbBr_3$	$CeBr_3$	8
6	Co(II)-$CsPbBr_3$	$Co(OAc)_2$	4
7	Mn(II)-$CsPbBr_3$	$MnBr_2$	5

Various metal precursors (0.008 mmol) were added into 20 mg of $CsPbBr_3$ QDs resuspended in hexane (1 mL) respectively, and the mixture was stirred vigorously in the dark for 1 h. The resulting solution mixture was centrifuged at 9000 rpm for 1 min. The supernatant was discarded, and the metal-$CsPbBr_3$ QDs were dried at room temperature[6].

Supplementary Table 11. Survey of solvents.

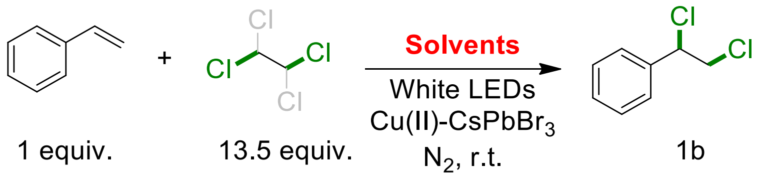

Entry	Solvents	Yield [%]
1	DCM	<1
2	THF	2
3	Toluene	2
4	EtOAc	4
5	1,4-Dioxane	3
6	MeCN	54

Supplementary Table 12. Control experiments.

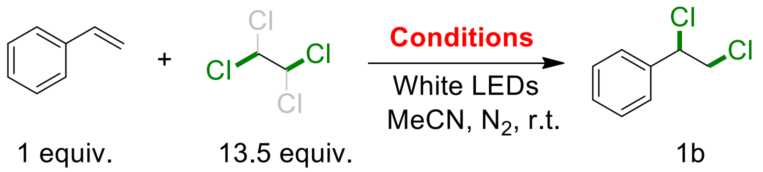

Entry	Catalysts	Amount	Light	Chlorine source	Yield [%]
1	Cu-$CsPbBr_3$	4 mg	√	√	56
2	Cu-$CsPbBr_3$	4 mg	×	√	Trace
3	Cu-$CsPbBr_3$	4 mg	√	×	0
4	Cu-$CsPbBr_3$	0 mg	√	√	Trace
5	$CuCl_2$	1.1 mg	√	√	2
6	CuBr	1.1 mg	√	√	1
7	$CuBr_2$	1.7 mg	√	√	2

Note: 0.008 mmol of CuX_{1-2} were used.

Supplementary Table 13. Survey of transition metal catalysts.

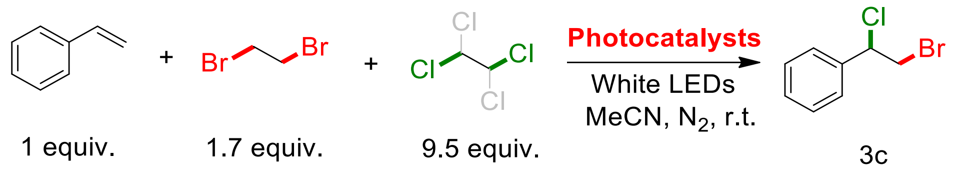

Entry	Photocatalysts	Metal precursors	Yield [%]
1	Cu(II)-$CsPbBr_3$	$CuBr_2$	45
2	Cu(I)-$CsPbBr_3$	CuBr	37
3	Fe(III)-$CsPbBr_3$	$FeCl_3\cdot6H_2O$	11
4	Ni(II)-$CsPbBr_3$	$NiCl_2\cdot6H_2O$	15
5	Ce(III)-$CsPbBr_3$	$CeBr_3$	28
6	Co(II)-$CsPbBr_3$	$Co(OAc)_2$	12
7	Mn(II)-$CsPbBr_3$	$MnBr_2$	10
8	Sn(II)-$CsPbBr_3$	$SnBr_2$	5
9	Ag(I)-$CsPbBr_3$	$AgNO_3$	6
10	$CsPbBr_3$	No	7

Note: Sn-$CsPbBr_3$ QDs were prepared via a reported method in literature[7].

21. Spectral data for products

(1,2-dibromoethyl)benzene (1a)

General procedure 1: Using 0.5 mmol DBE as the donor, after **36** h, the mixture was purified by column chromatography (PE/EtOAc=100:1) yielding the title compound (16.6 mg, yield: 63 %). HRMS for $C_8H_5Br_2$ (ESI+) $[M+H]^+$ calc.: 264.9051, found: 264.9045.

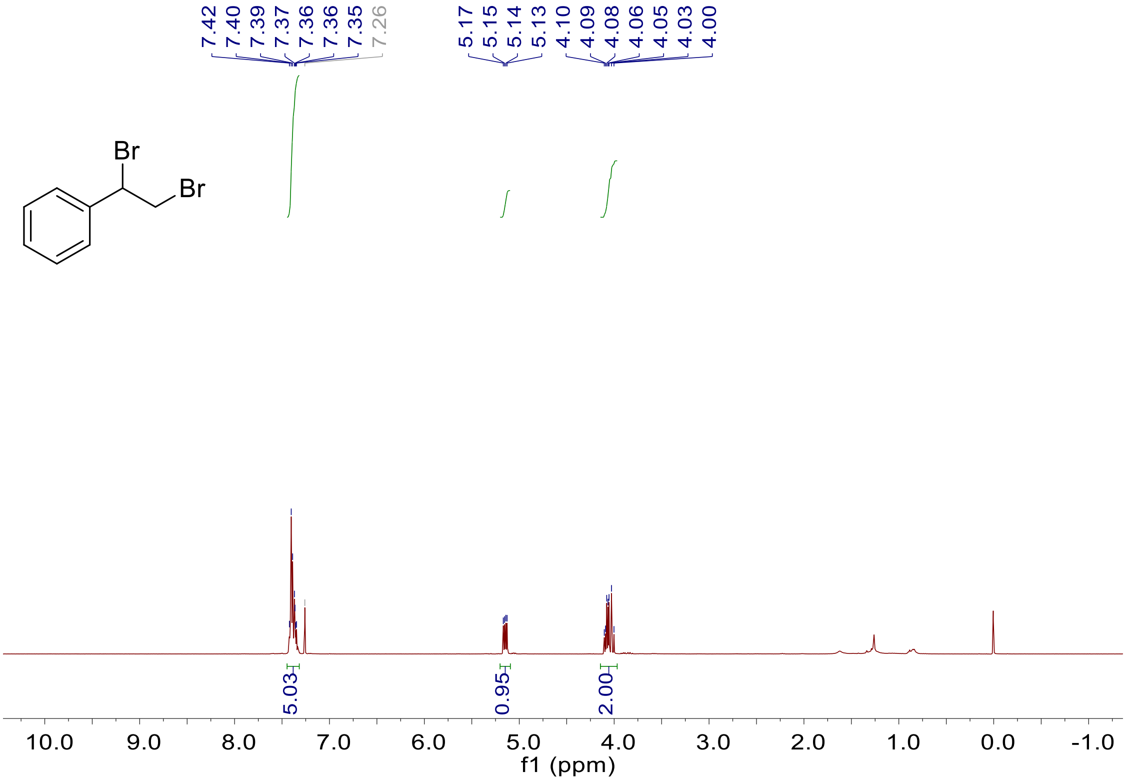

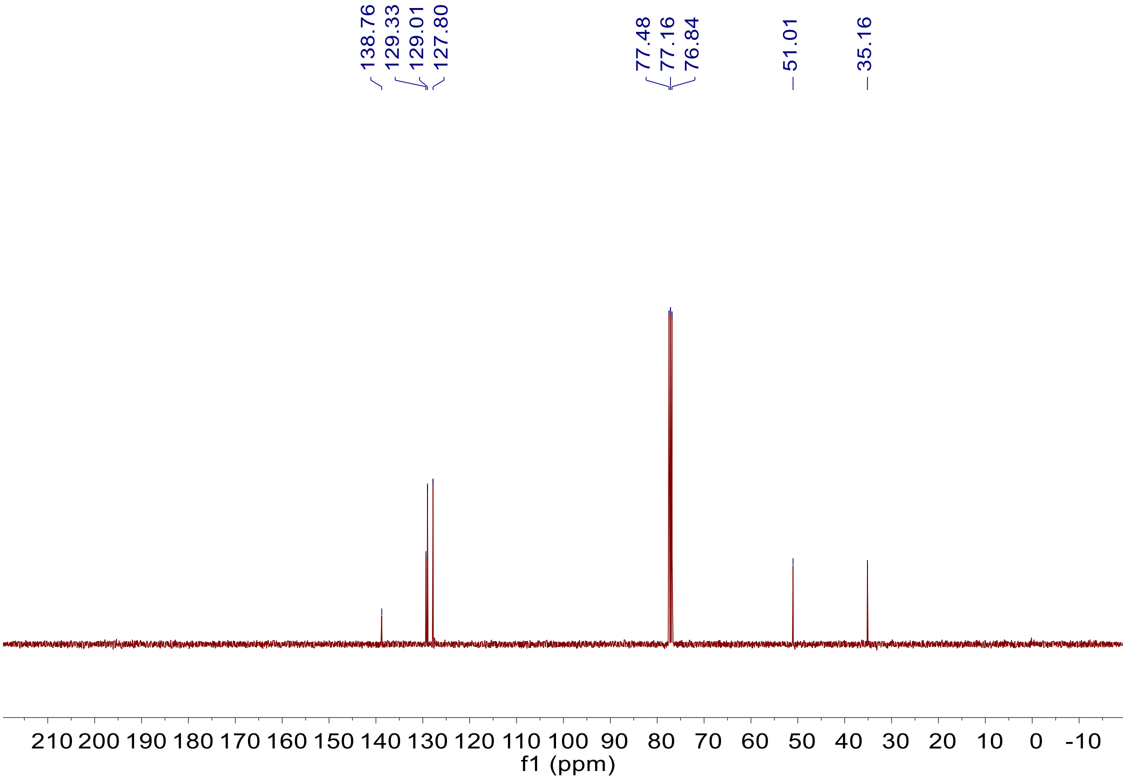

1-(1,2-dibromoethyl)-4-fluorobenzene (2a)

General procedure 1: Using 0.5 mmol DBE as the donor, after **24** h, the mixture was purified by column chromatography (PE/EtOAc=100:1) yielding the title compound (19.7 mg, yield: 70 %). HRMS for $C_8H_7Br_2F$ (ESI+) $[M-H]^-$ calc.: 280.8800, found: 280.8822.

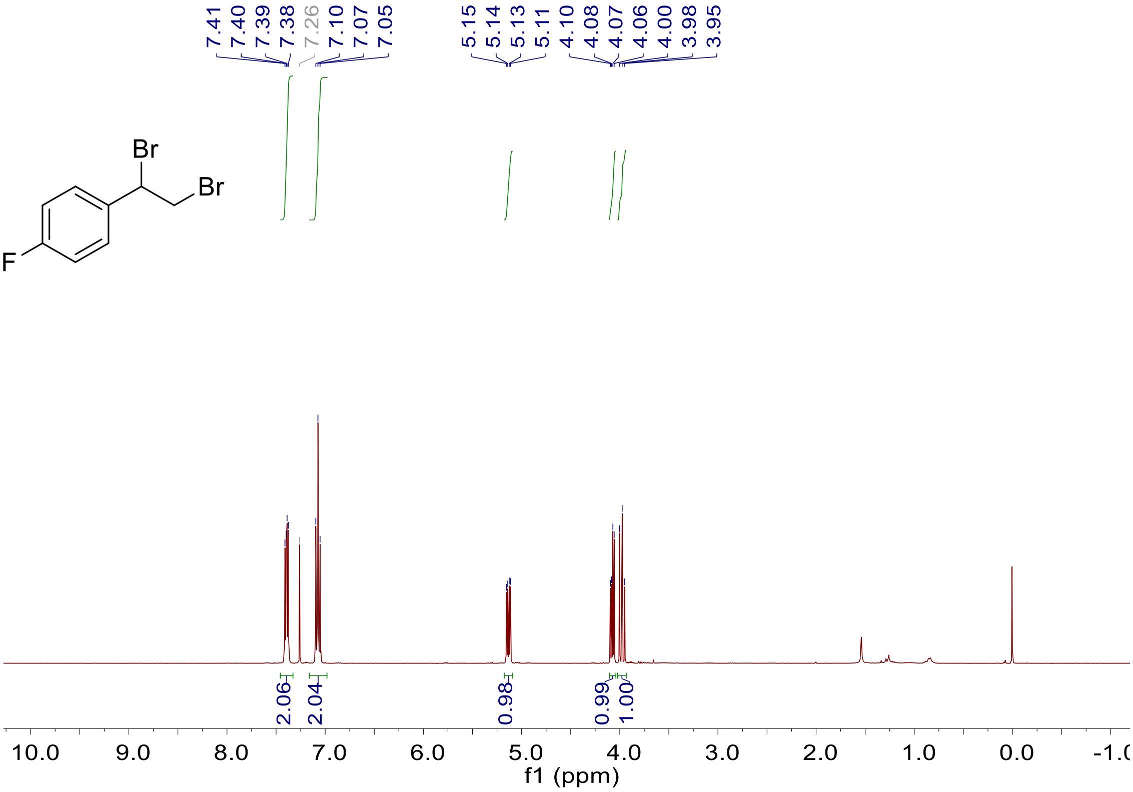

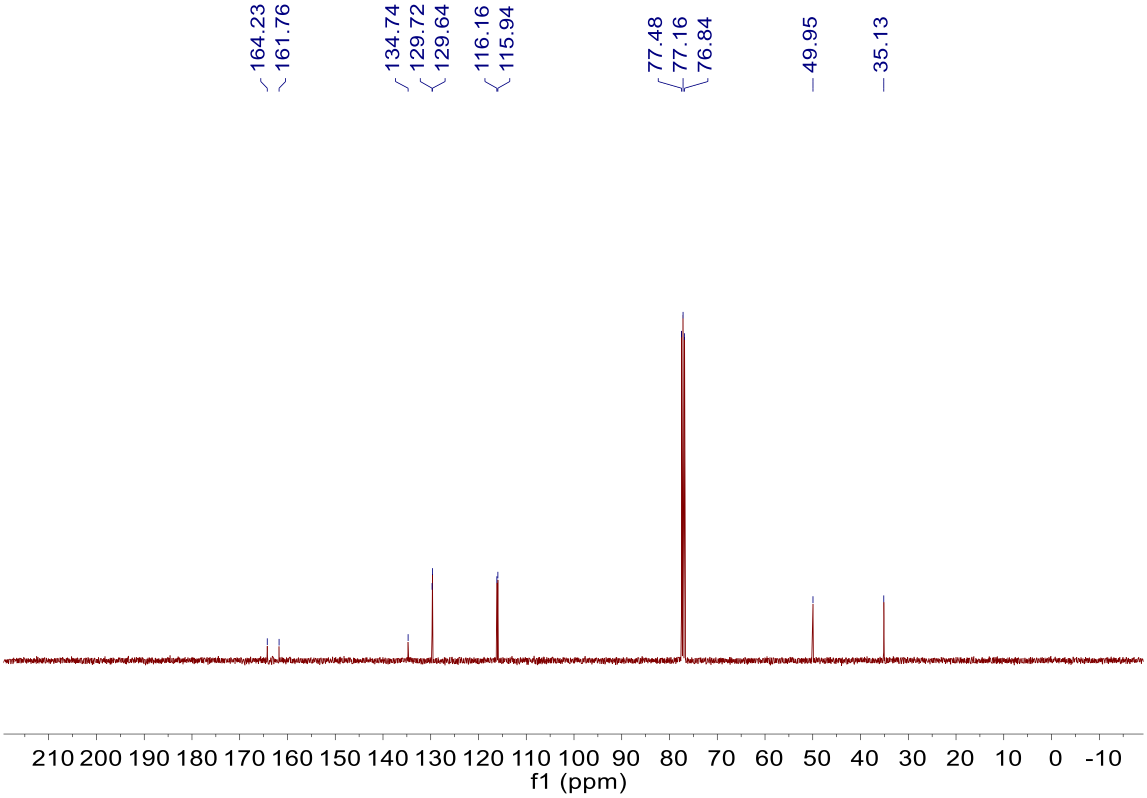

1-(1,2-dibromoethyl)-3-fluorobenzene (3a)

General procedure 1: Using 0.5 mmol DBE as the donor, after **36** h, the mixture was purified by column chromatography (PE/EtOAc=100:1) yielding the title compound (13.8 mg, yield: 49 %). HRMS for $C_8H_7Br_2F$ (ESI+) $[M-Br]^+$ calc.: 200.9715, found: 200.9706.

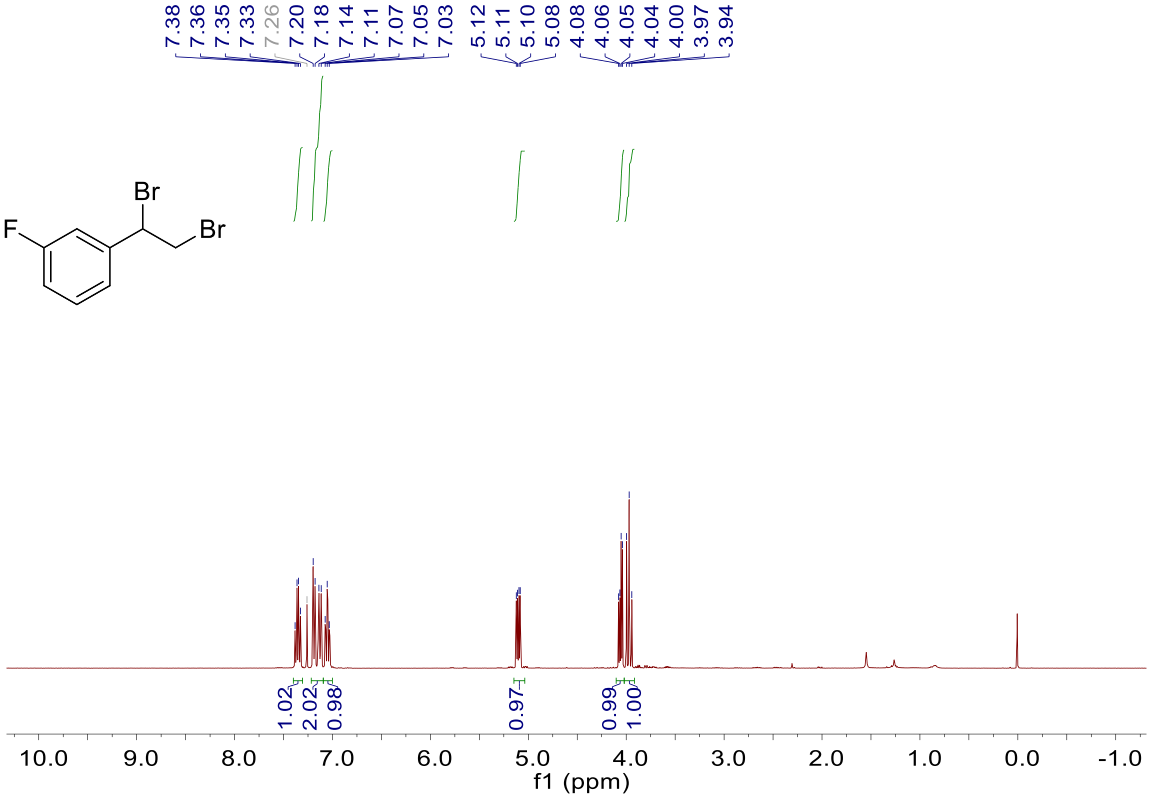

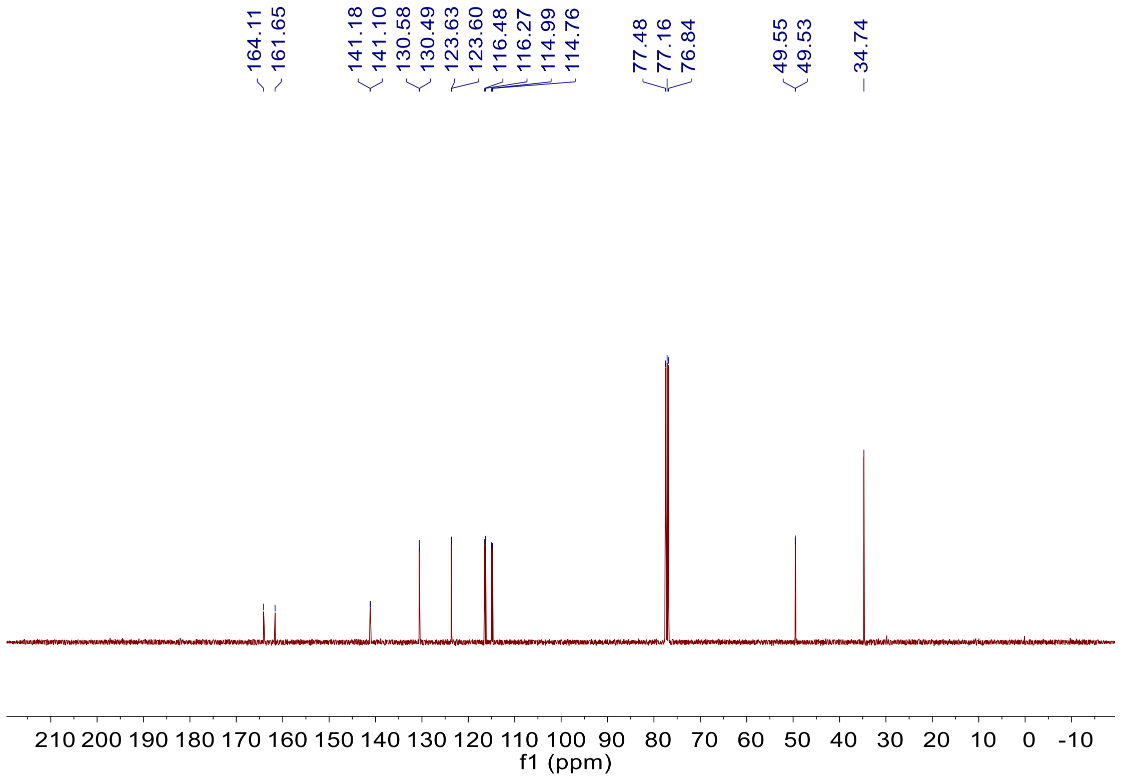

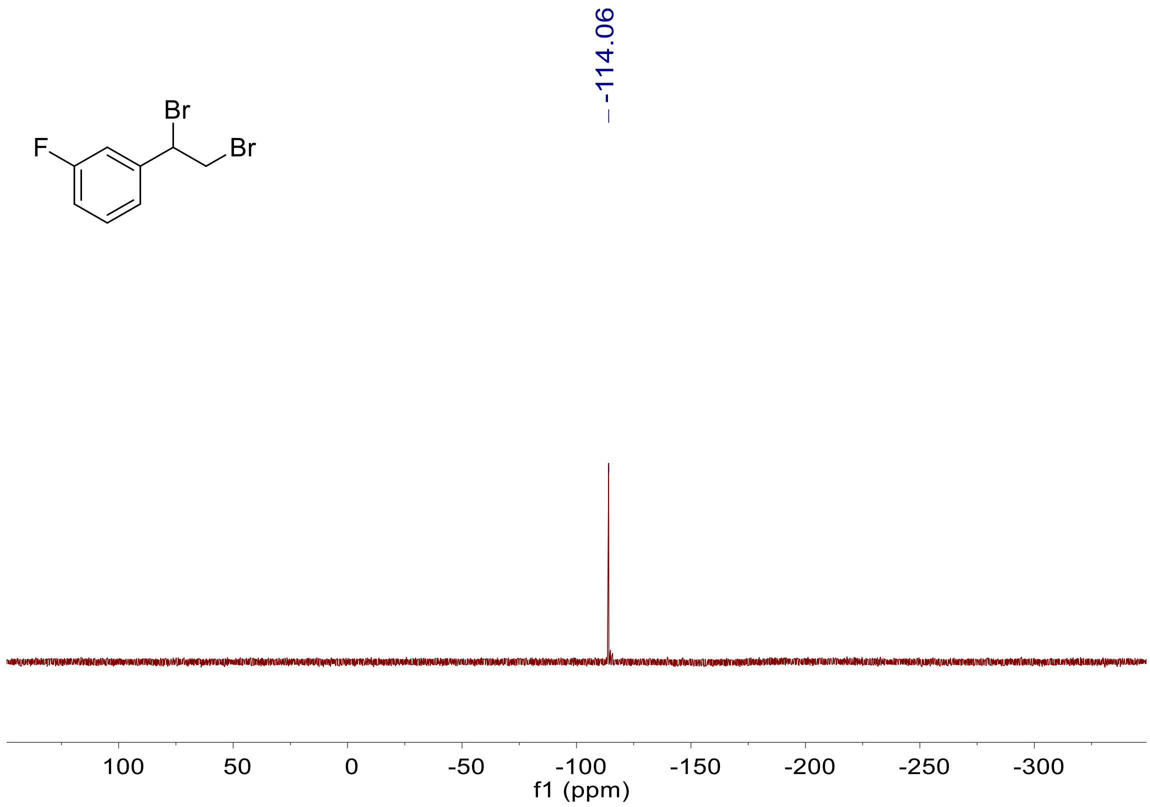

F
Br
Br
-114.06
100
50
0
-50
-100
-150
-200
-250
-300
f1 (ppm)

1-(1,2-dibromoethyl)-2-fluorobenzene (4a)

General procedure 1: Using 0.5 mmol DBE as the donor, after **30** h, the mixture was purified by column chromatography (PE/EtOAc=100:1) yielding the title compound (14.9 mg, yield: 53 %). HRMS for $C_8H_7Br_2F$ (ESI+) $[M-H]^-$ calc.: 280.8800, found: 280.8808.

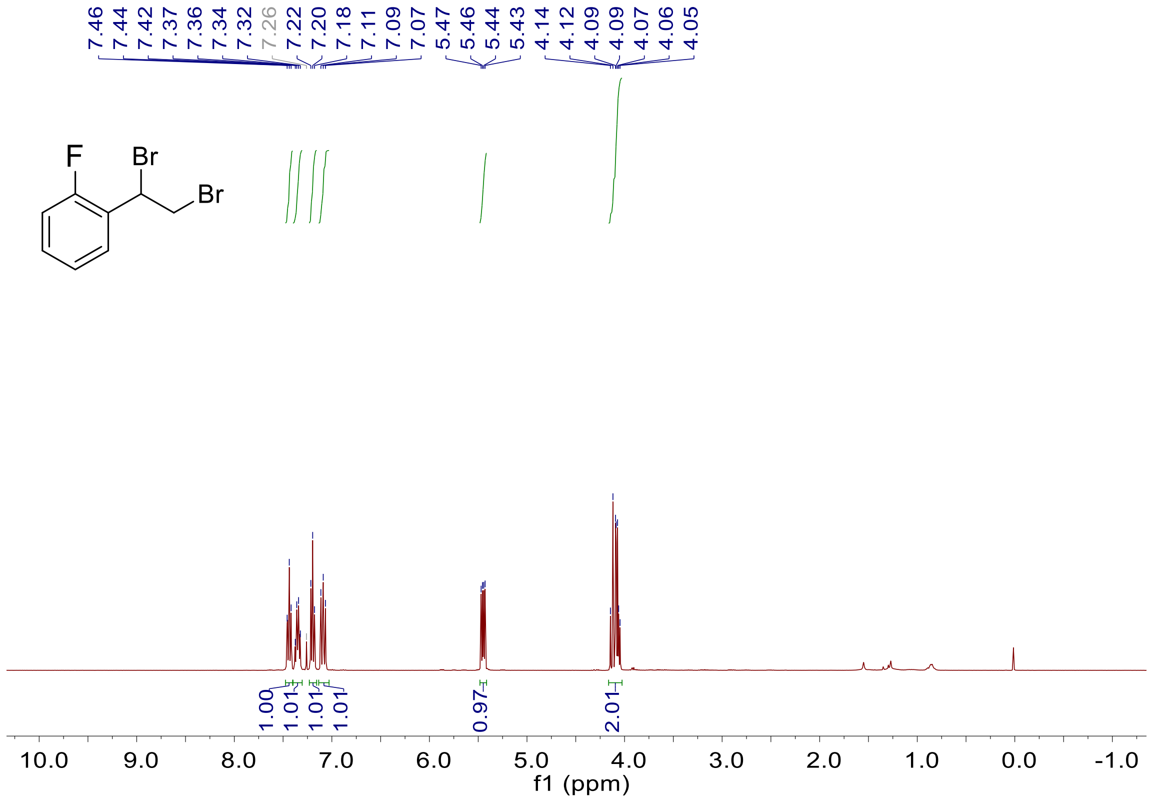

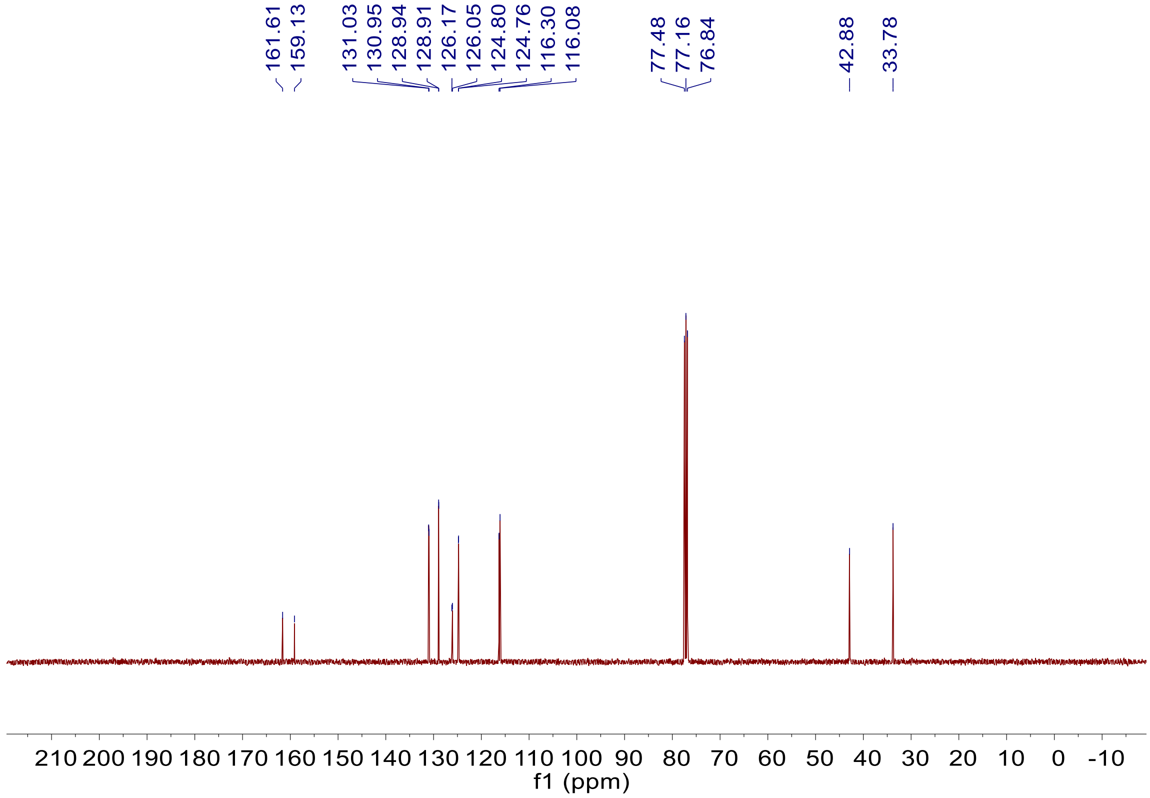

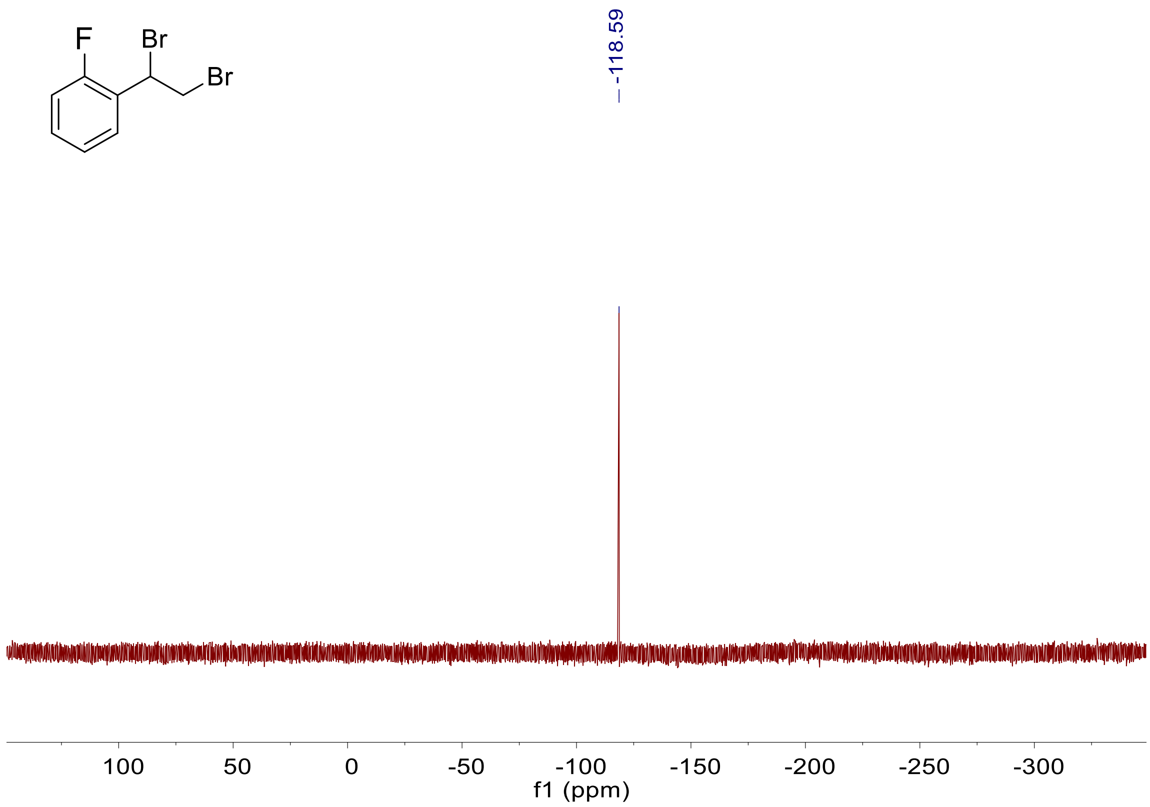
F
Br
Br
-118.59
100
50
0
-50
-100
-150
-200
-250
-300
f1 (ppm)

4-(1,2-dibromoethyl)phenyl acetate (5a)

General procedure 1: Using 0.75 mmol DBE as the donor, after **42** h, the mixture was purified by column chromatography (PE/EtOAc=10:1) yielding the title compound (14.8 mg, yield: 46 %). HRMS for $C_{10}H_{10}Br_2O_2$ (ESI+) $[M-Br]^+$ calc.: 240.9864, found: 240.9853.

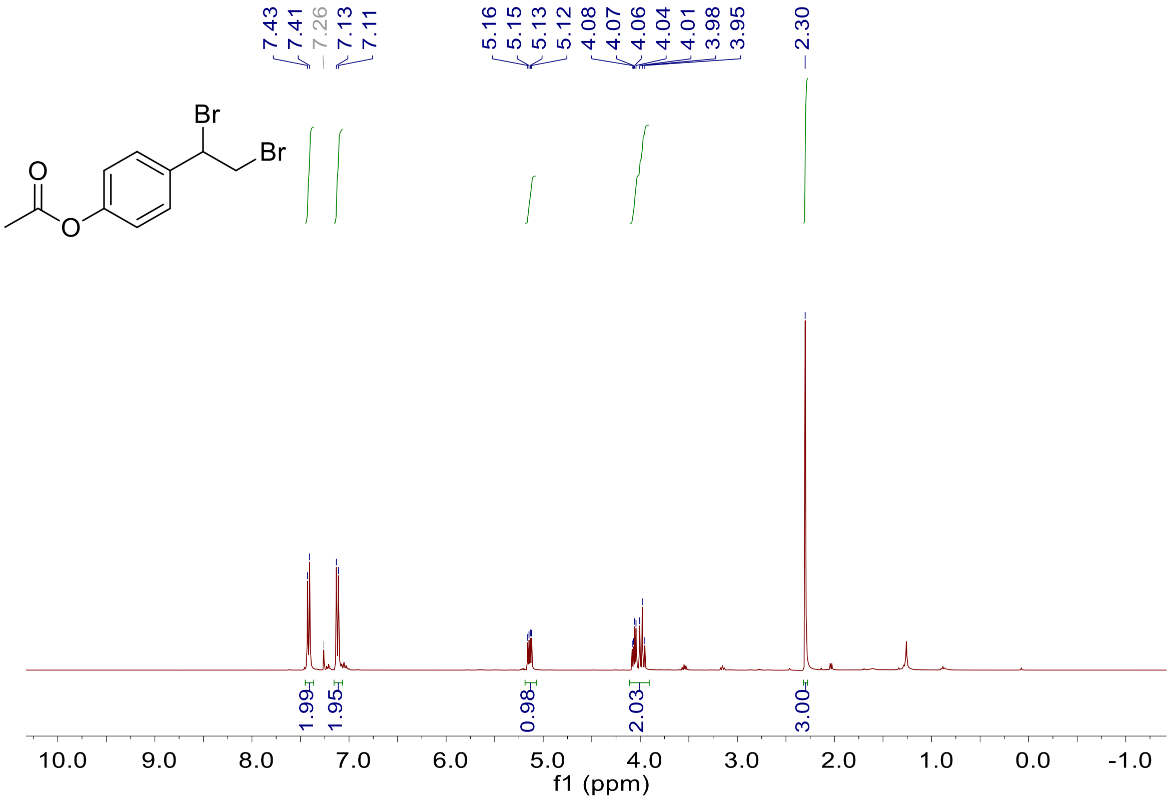

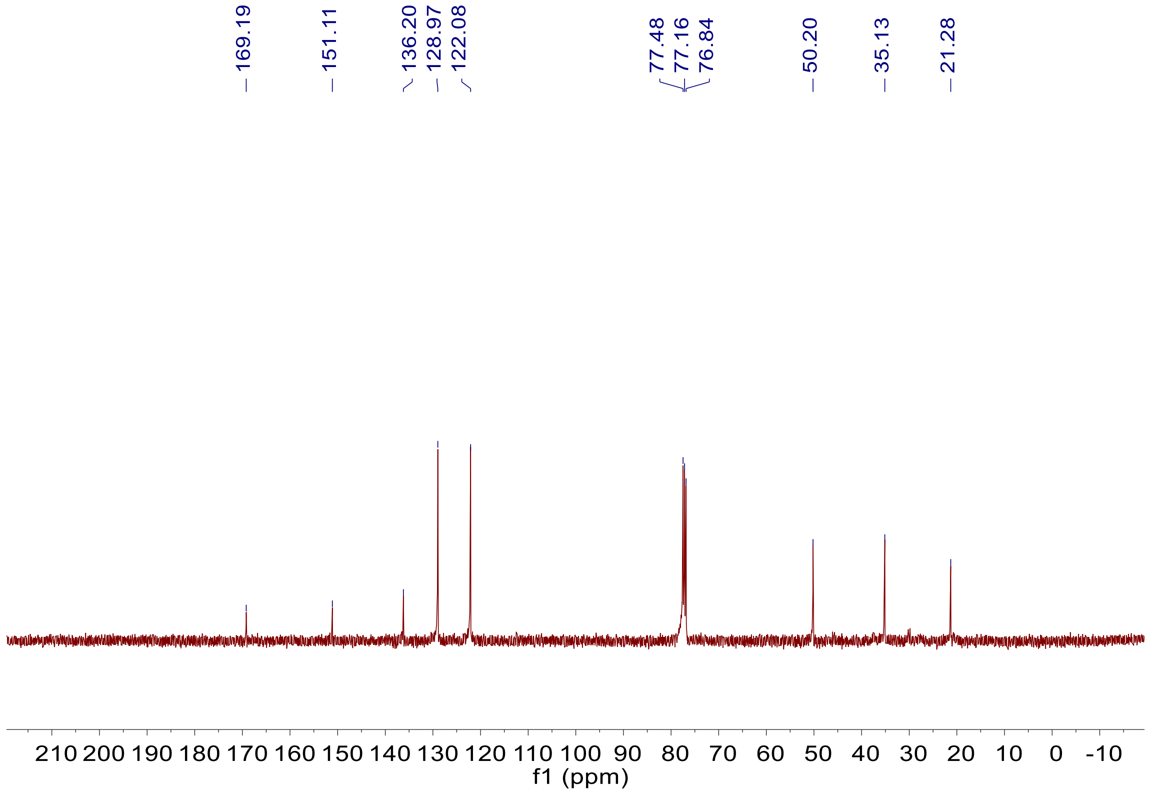

1-(tert-butyl)-4-(1,2-dibromoethyl)benzene (6a)

General procedure 1: Using 0.5 mmol DBE as the donor, after **42** h, the mixture was purified by column chromatography (PE/EtOAc=100:1) yielding the title compound (16.3 mg, yield: 51 %). HRMS for $C_{12}H_{16}Br_2$ (ESI+) $[M-Br]^+$ calc.: 241.0415, found: 241.0408.

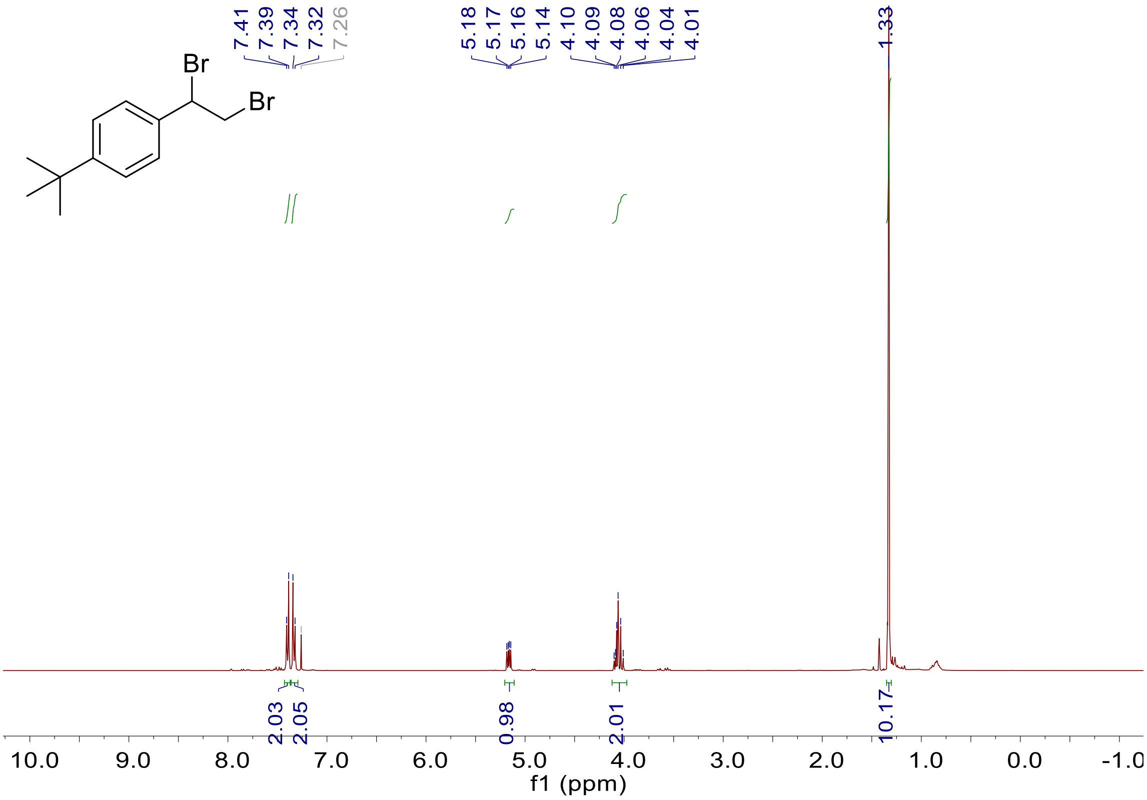

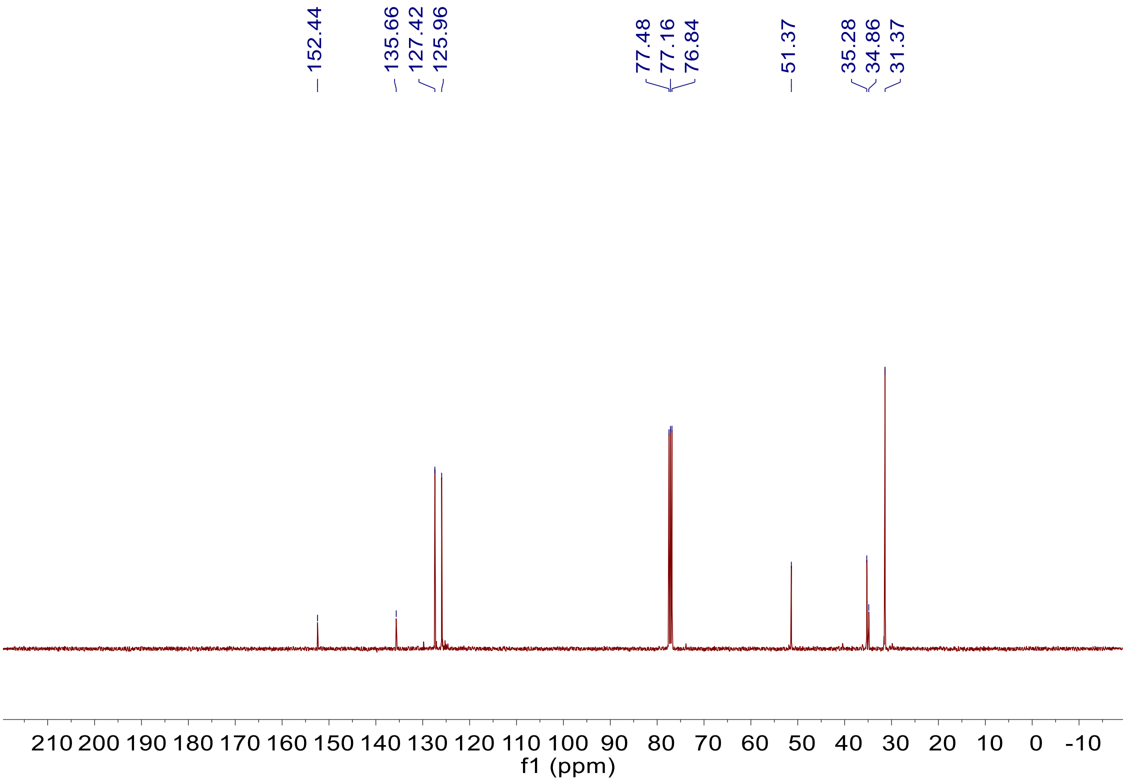

1-chloro-4-(1,2-dibromoethyl)benzene (7a)

General procedure 1: Using 0.5 mmol DBE as the donor, after **50** h, the mixture was purified by column chromatography (PE/EtOAc=50:1) yielding the title compound (19.3 mg, yield: 65 %). HRMS for $C_8H_7Br_2Cl$ (ESI+) $[M-Br]^+$ calc.: 218.9399, found: 218.9390.

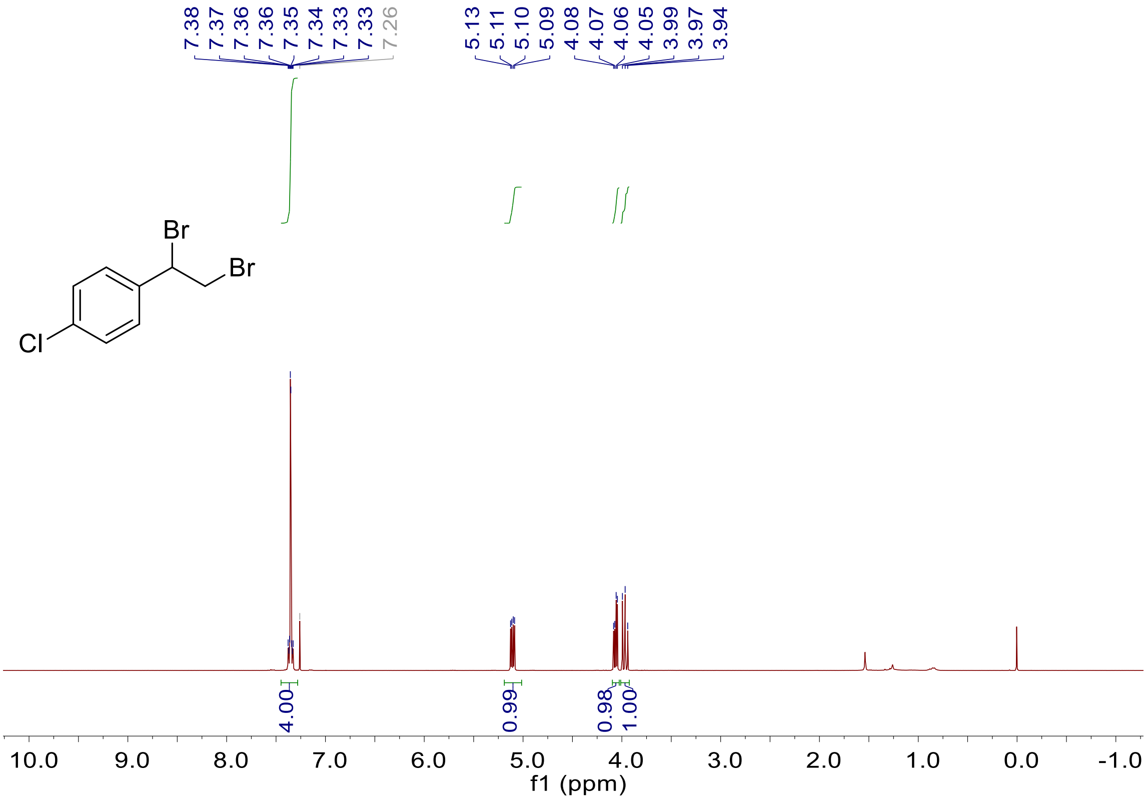

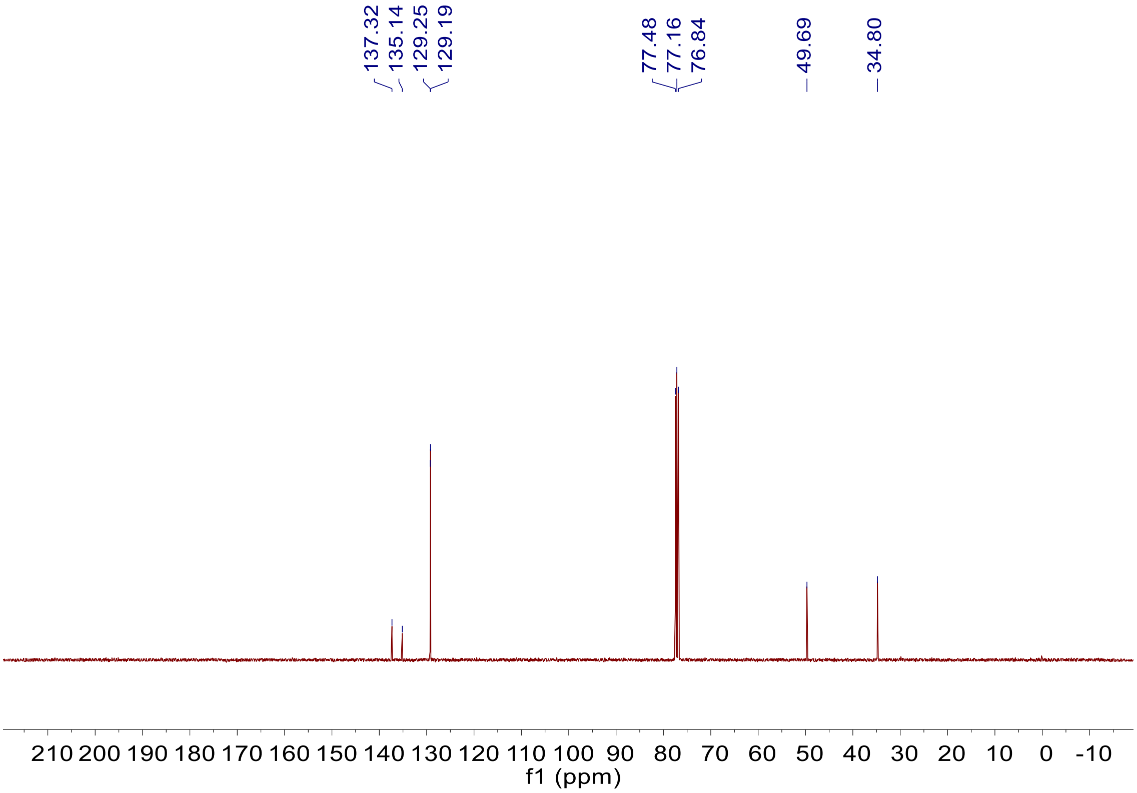

1-(1,2-dibromoethyl)-2,3,4,5,6-pentafluorobenzene (8a)

General procedure 1: Using 0.75 mmol DBE as the donor, after **48 h**, the mixture was purified by column chromatography (PE/EtOAc=100:1) yielding the title compound (15.2 mg, yield: 43 %). HRMS for $C_8H_3Br_2F_5$ (ESI+) $[M{-}Br{+}NH_4]^+$ calc.: 290.9682, found: 290.9649.

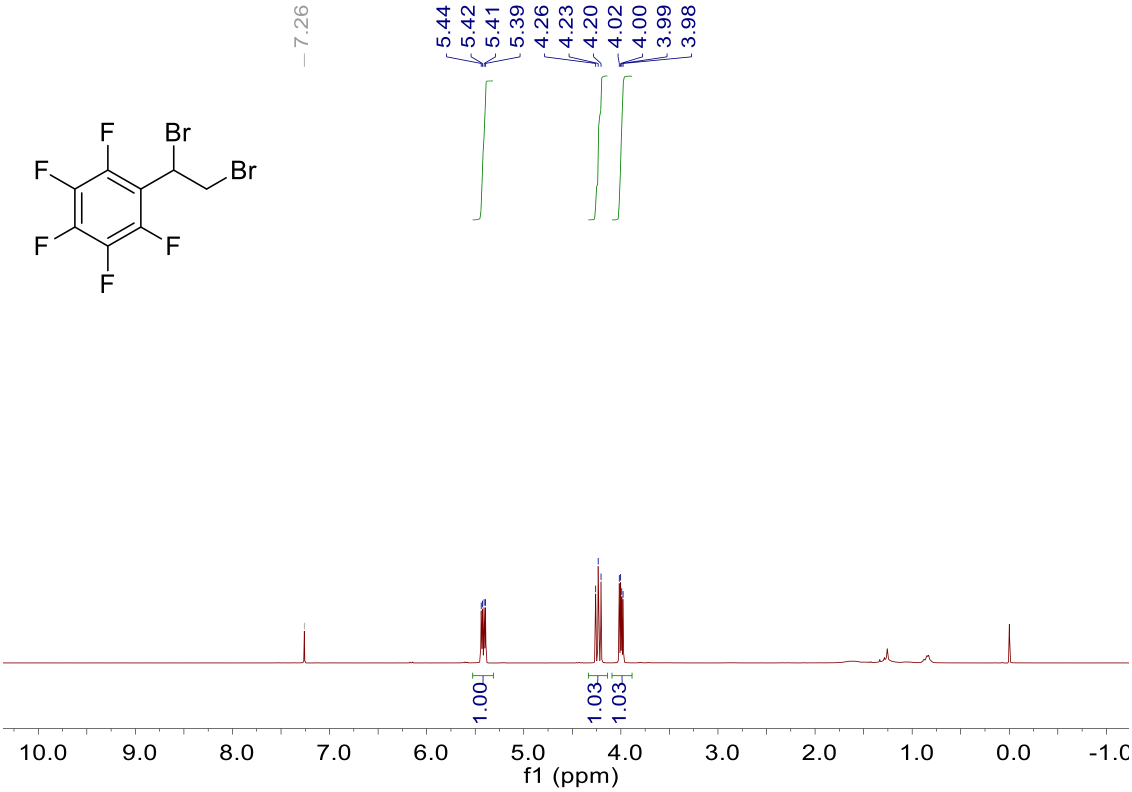

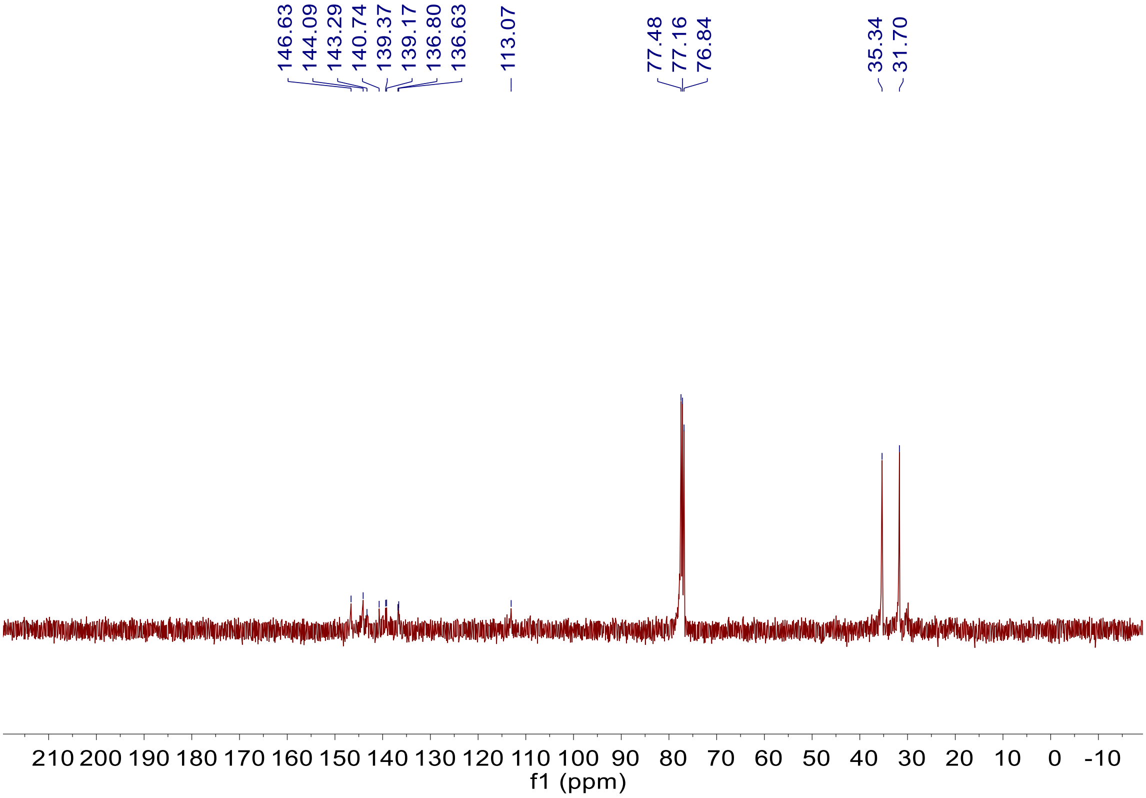

(1,2-dibromoethyl)dimethyl(phenyl)silane (9a)

General procedure 1: Using 0.75 mmol DBE as the donor, after **43** h, the mixture was purified by column chromatography (PE/EtOAc=100:1) yielding the title compound (20.0 mg, yield: 62 %). HRMS for $C_{10}H_{14}Br_2Si$ (ESI+) $[M-Br]^+$ calc.: 243.0028, found: 243.0169.

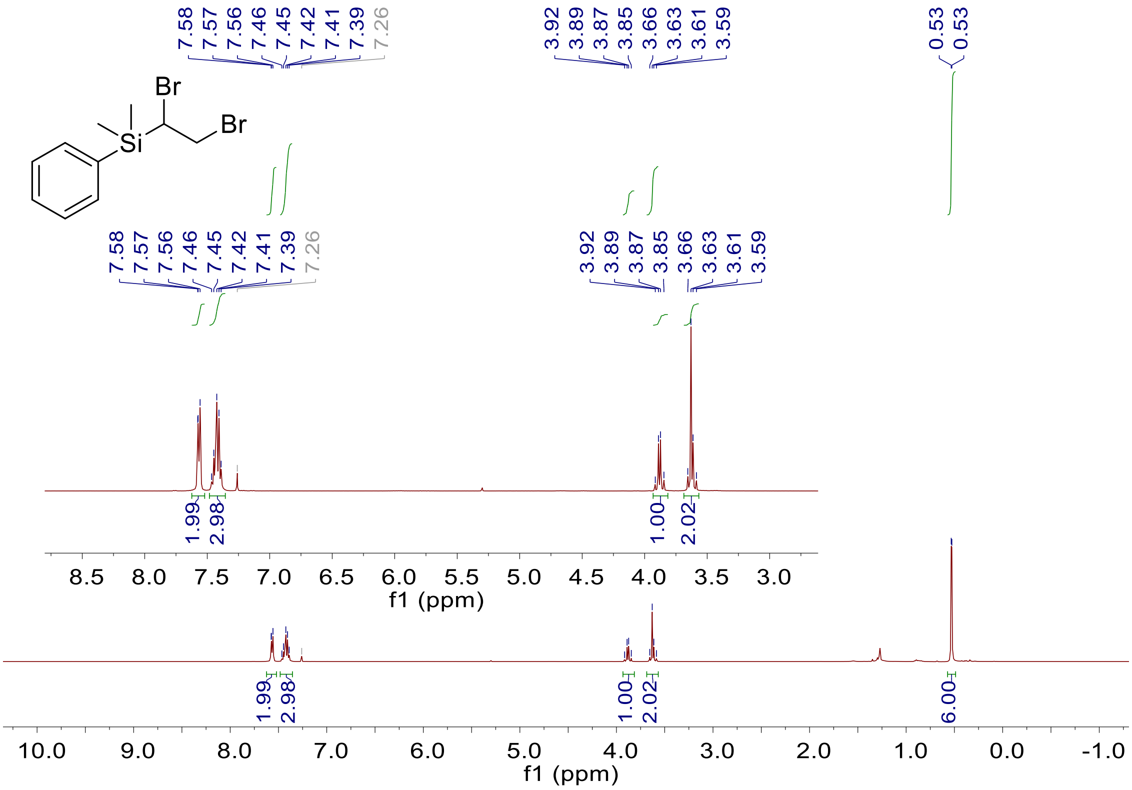

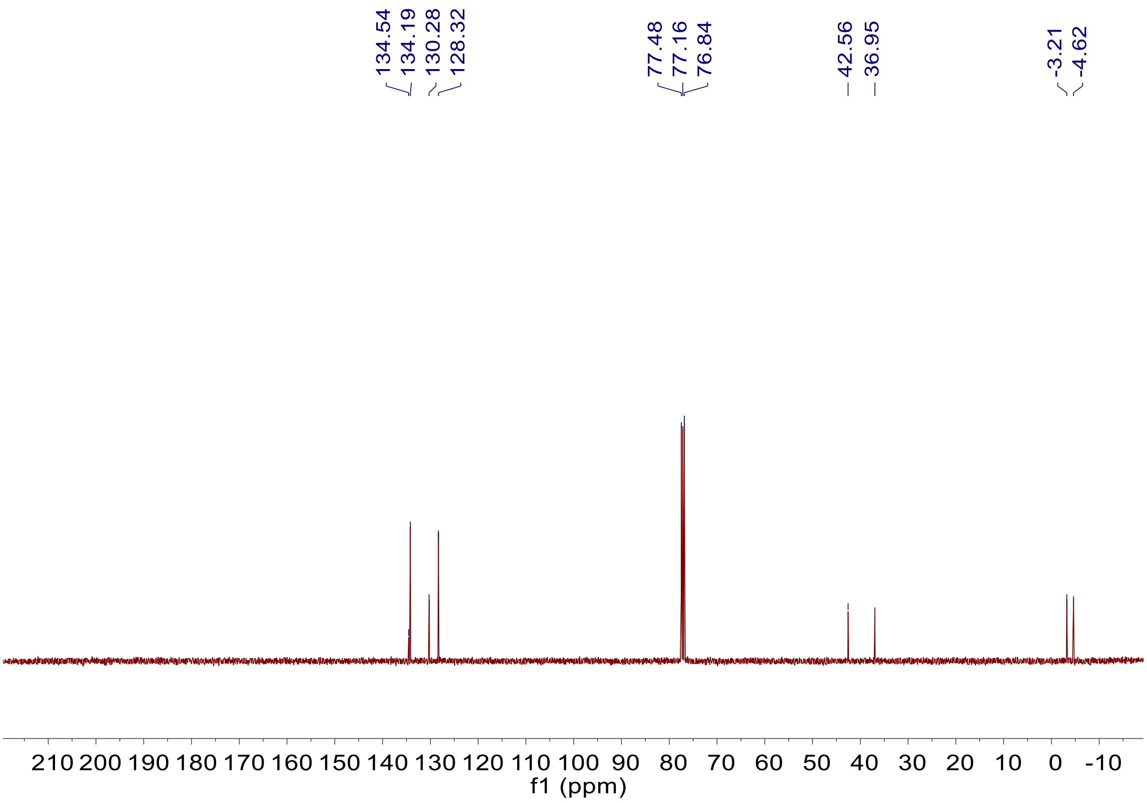

(1,2-dibromopropyl)benzene (12a)

General procedure 1: Using 0.75 mmol DBE as the donor, after **40** h, the mixture was purified by column chromatography (PE/EtOAc=100:1) yielding the title compound (14.6 mg, yield: 53 %). HRMS for $C_9H_{10}Br_2$ (ESI+) $[M-Br]^+$ calc.: 198.9945, found: 198.9928.

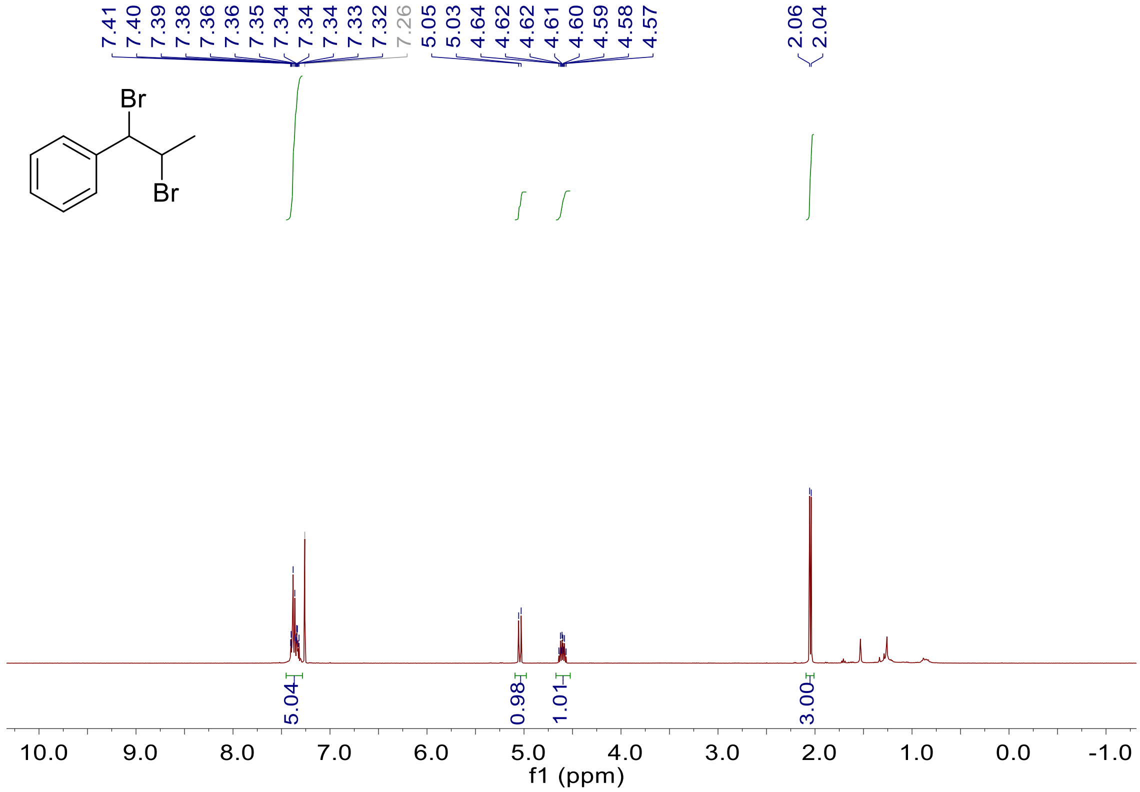

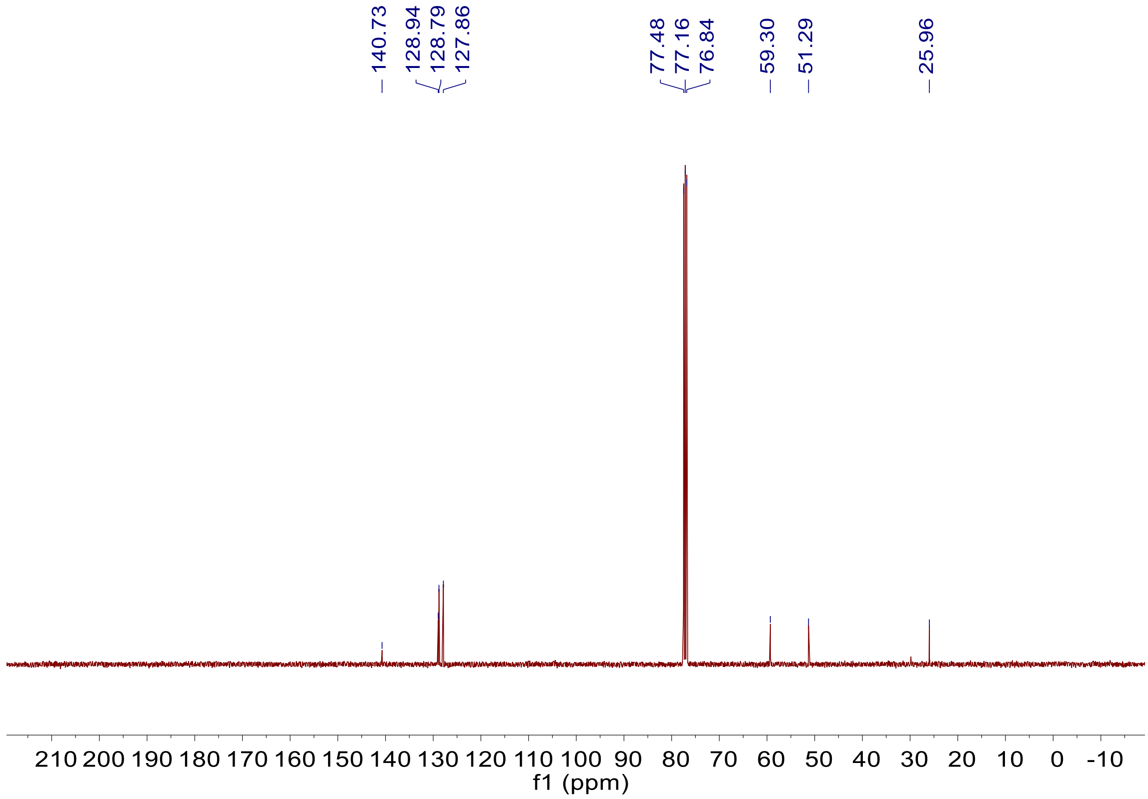

methyl 2,3-dibromo-3-phenylpropanoate (13a)

General procedure 1: Using 0.75 mmol DBE as the donor, after **72** h, the mixture was purified by column chromatography (PE/EtOAc=10:1) yielding the title compound (12.5 mg, yield: 39 %). HRMS for $C_{10}H_{10}Br_2O_2$ (ESI+) $[M-Br]^+$ calc.: 240.9864, found: 240.9850.

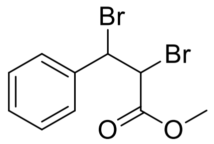

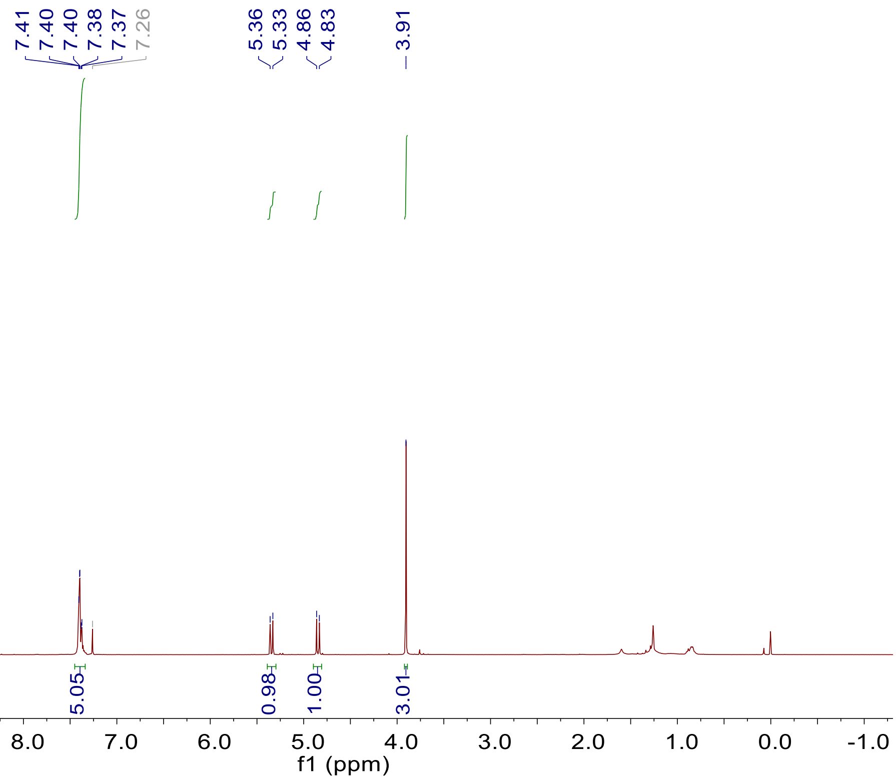

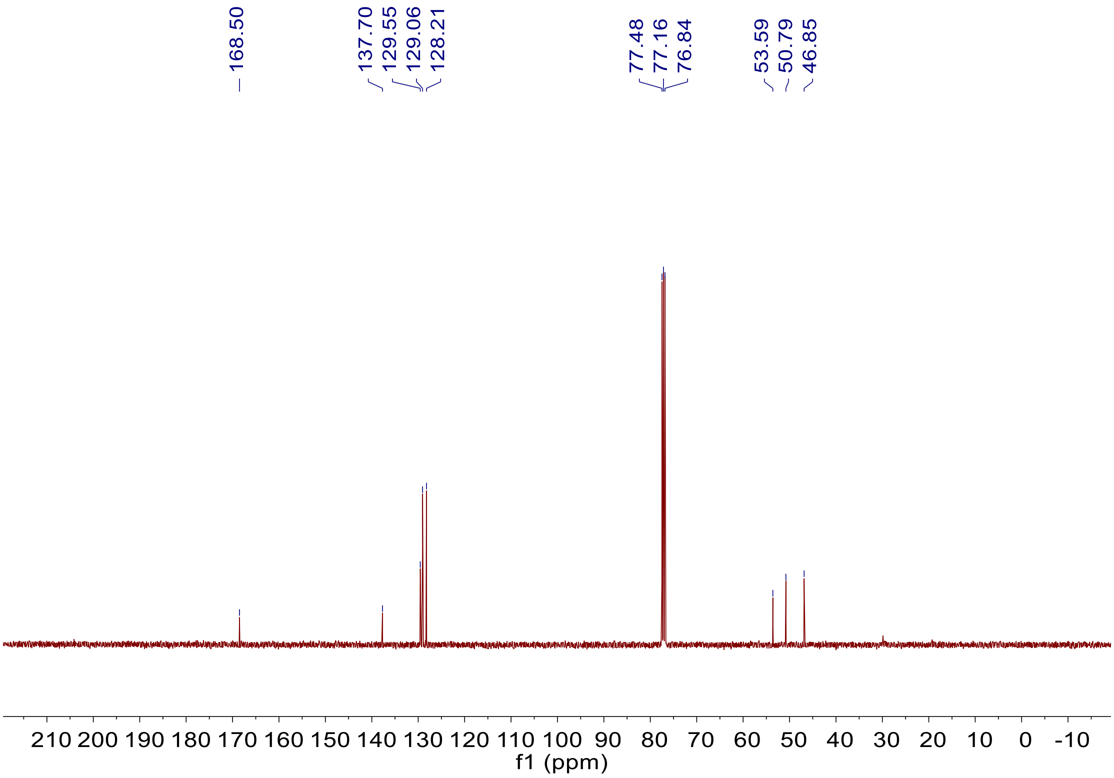

(1,2-dibromo-3-chloropropyl)benzene (14a)

General procedure 1: Using 0.75 mmol DBE as the donor, after **65** h, the mixture was purified by column chromatography (PE/EtOAc=10:1) yielding the title compound (17.1 mg, yield: 55 %). HRMS for $C_9H_9Br_2Cl$ (ESI+) $[M-Br]^+$ calc.: 232.9556, found: 232.9542.

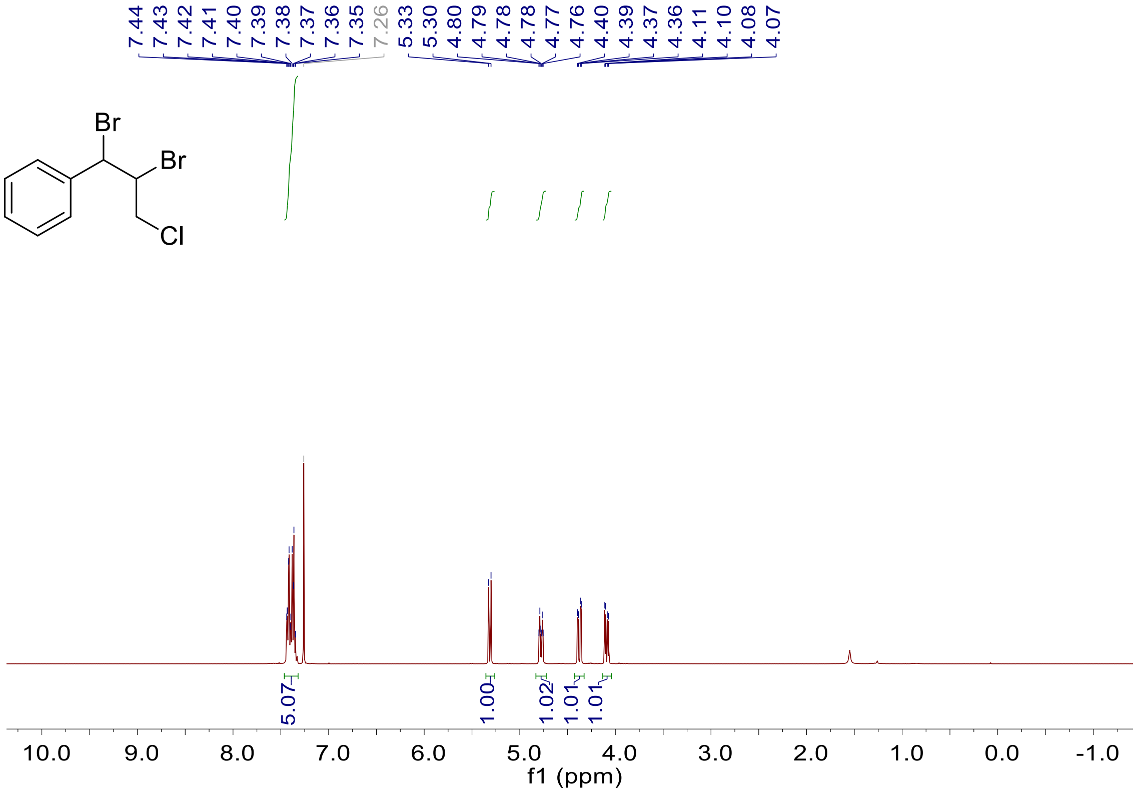

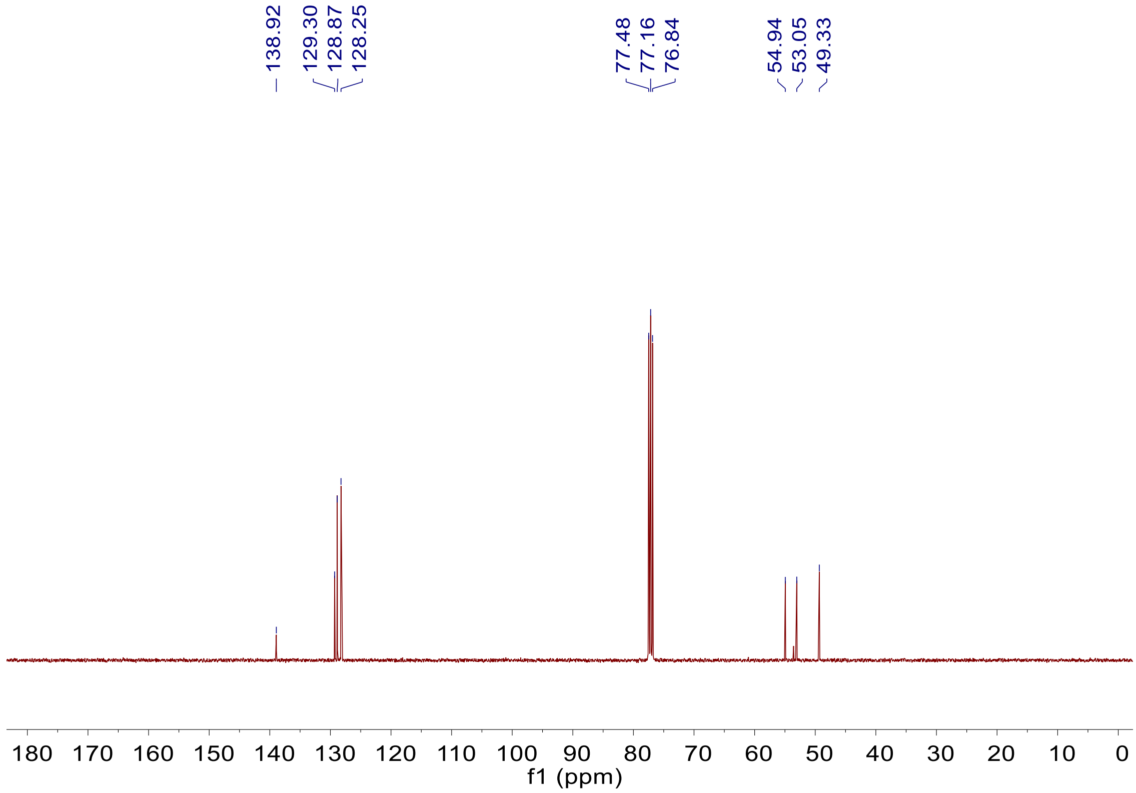

methyl 4-(1,2-dibromoethyl)benzoate (15a)

General procedure 1: Using 0.75 mmol DBE as the donor, after **55** h, the mixture was purified by column chromatography (PE/EtOAc=20:1) yielding the title compound (15.7 mg, yield: 49 %). HRMS for $C_{10}H_{10}Br_2O_2$ (ESI+) $[M-Br]^+$ calc.: 240.9864, found: 240.9854.

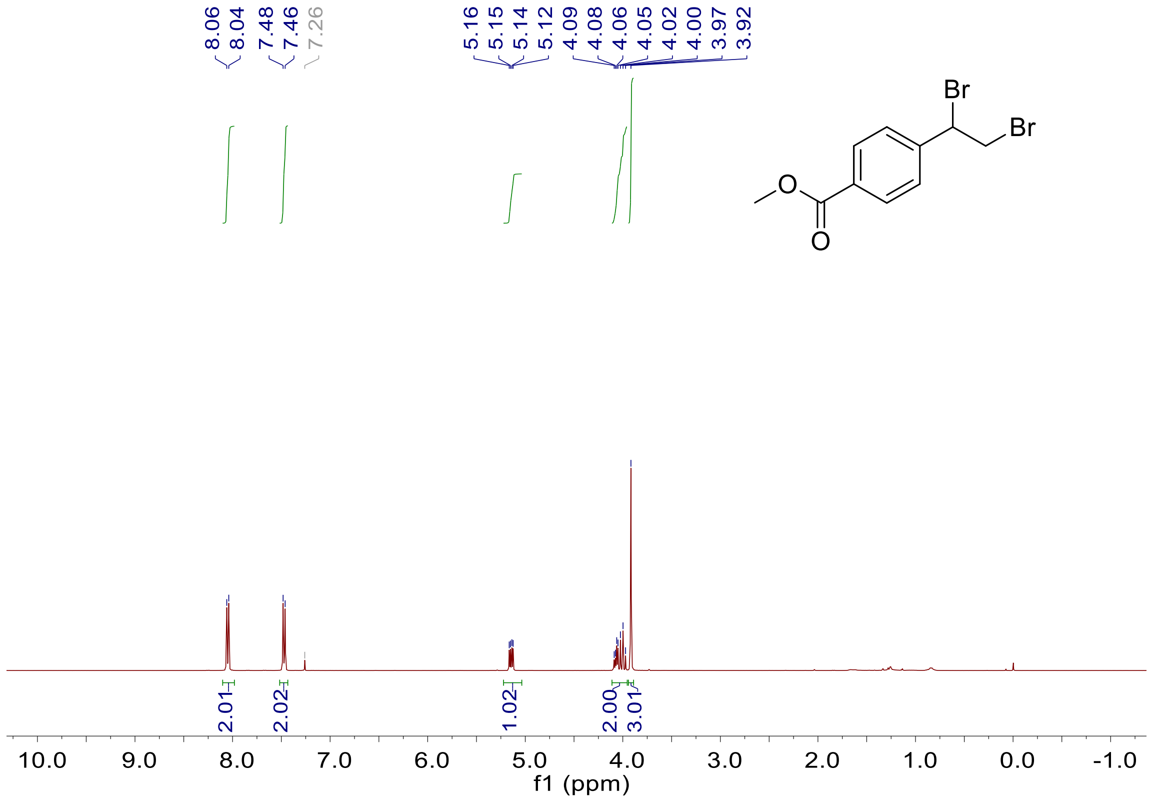

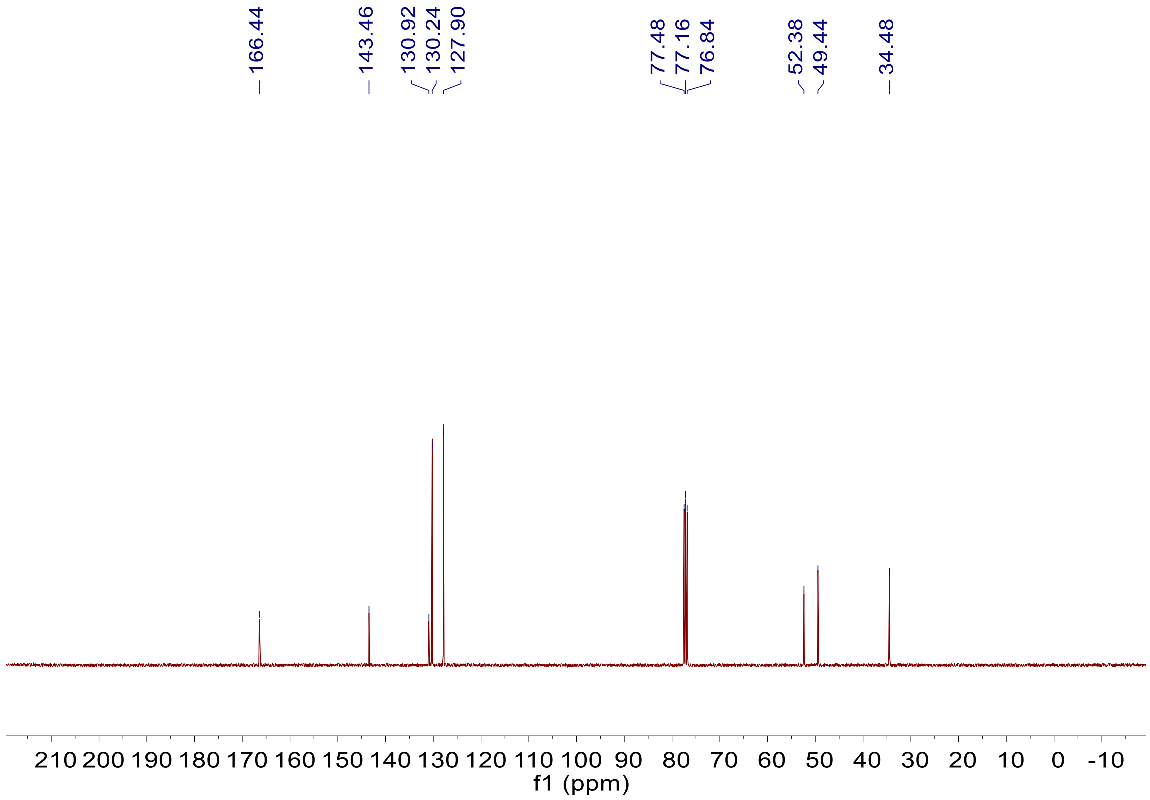

4-(1,2-dibromoethyl)benzonitrile (16a)

General procedure 1: Using 0.75 mmol 1,1,2-tribromoethane as the donor, after **48 h**, the mixture was purified by column chromatography (PE/EtOAc=10:1) yielding the title compound (10.1 mg, yield: 35 %). HRMS for $C_9H_7Br_2N$ (ESI+) $[M{-}H]^-$ calc.: 287.8847, found: 287.8853.

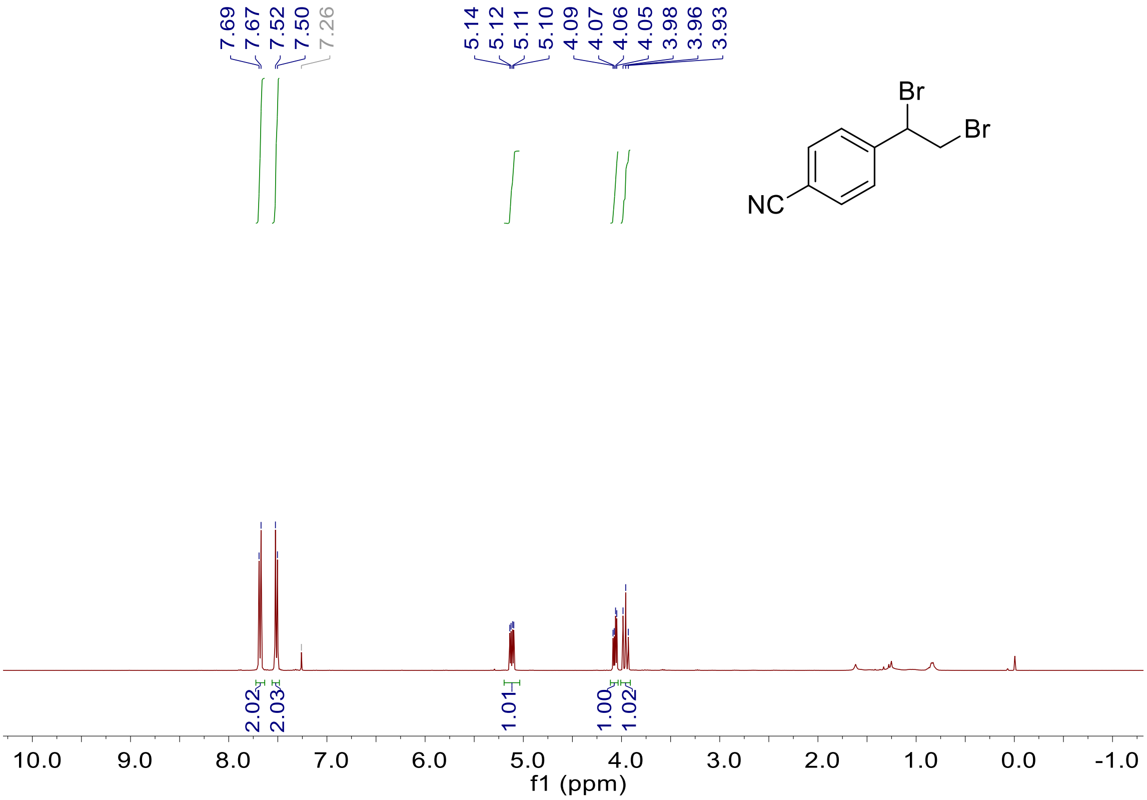

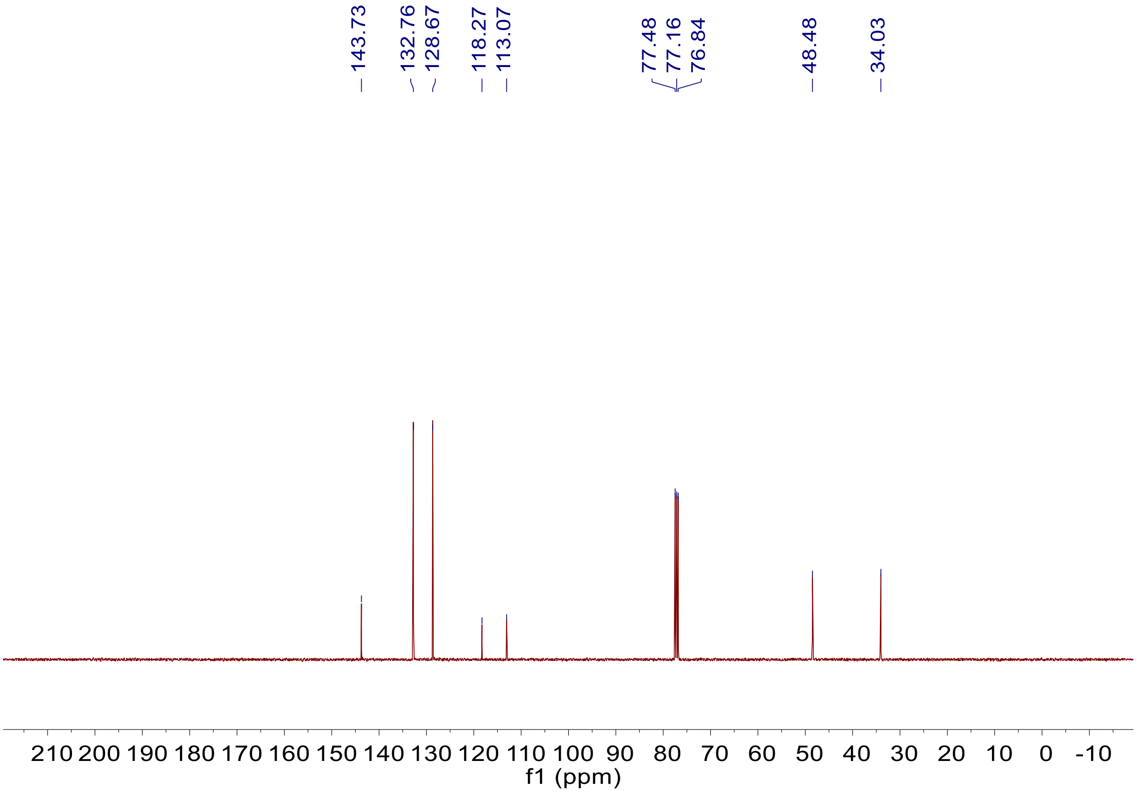

1-(1,2-dibromoethyl)-4-(trifluoromethyl)benzene (17a)

General procedure 1: Using 0.75 mmol DBE as the donor, after **48 h**, the mixture was purified by column chromatography (PE/EtOAc=100:1) yielding the title compound (15.9 mg, yield: 48 %). HRMS for $C_9H_7Br_2F_3$ (ESI+) $[M{-}Br]^+$ calc.: 250.9683, found: 250.9686.

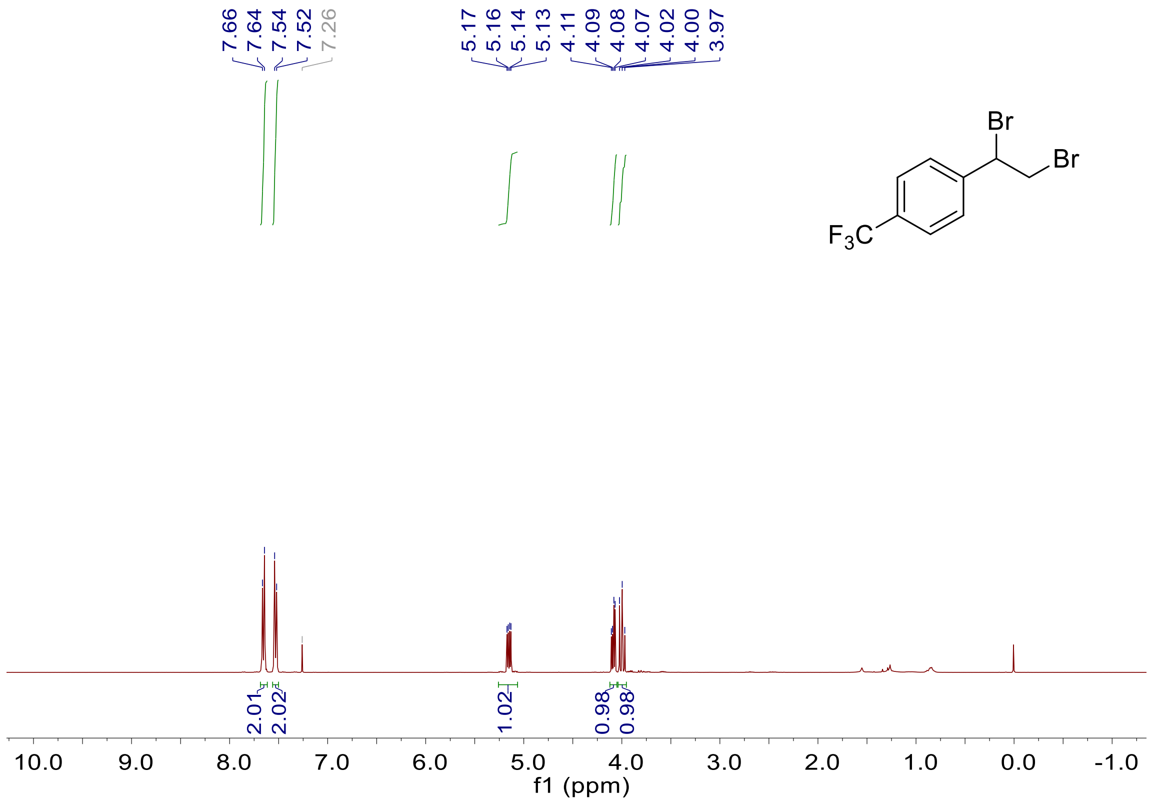

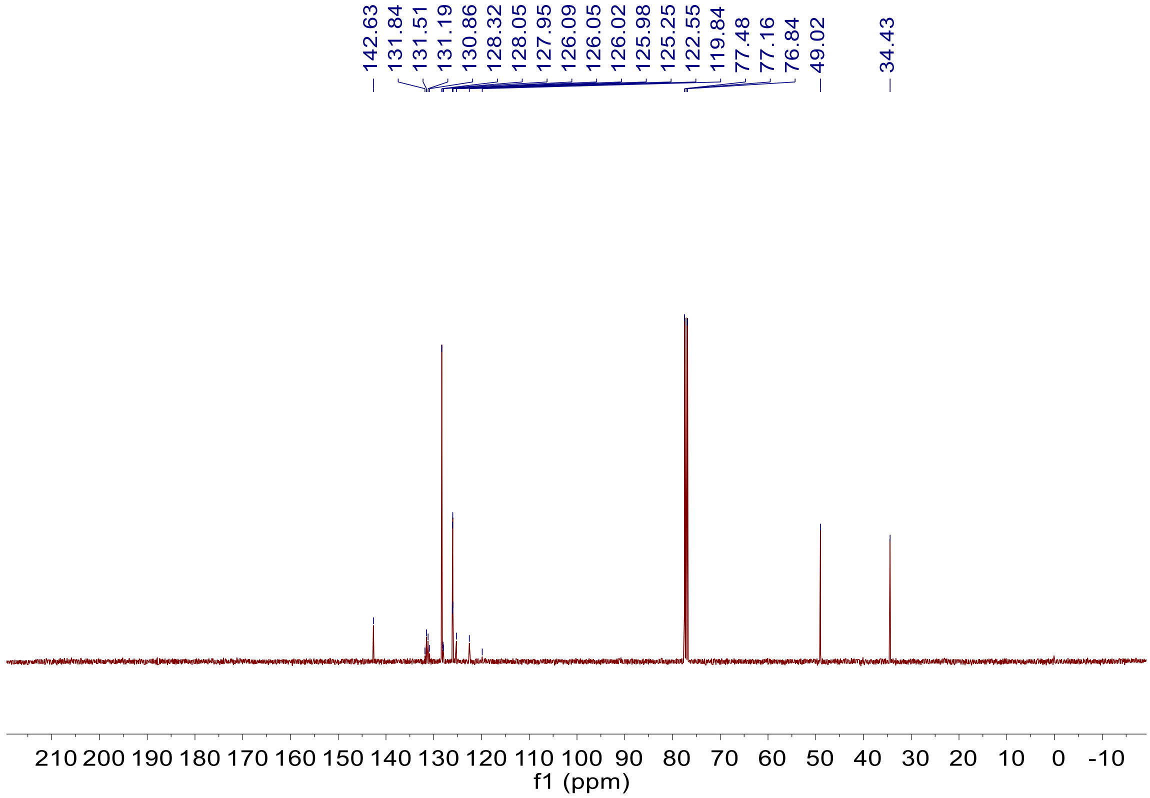

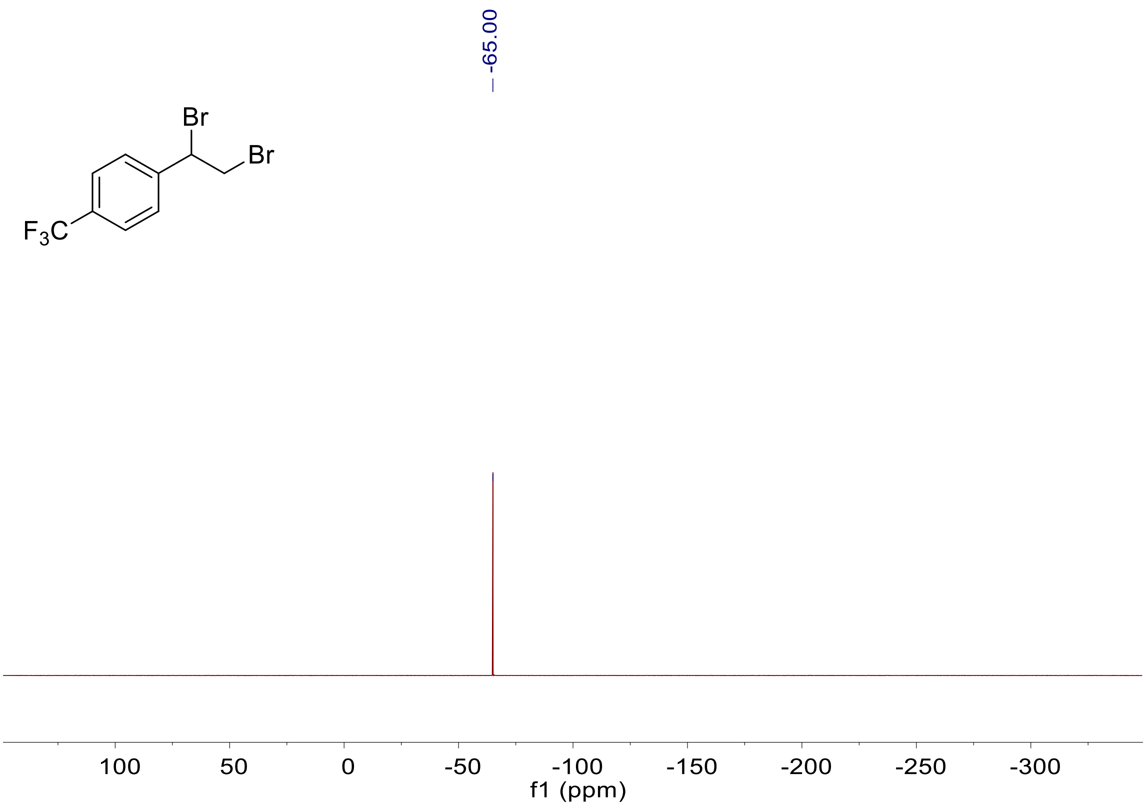
-65.00
Br
Br
F3C
100
50
0
-50
-100
-150
-200
-250
-300
f1 (ppm)

(4-(1,2-dibromoethyl)phenyl)trimethylsilane (18a)

General procedure 1: Using 0.75 mmol DBE as the donor, after **43 h**, the mixture was purified by column chromatography (PE/EtOAc=100:1) yielding the title compound (15.4 mg, yield: 46 %). HRMS for $C_{11}H_{16}Br_2Si$ (ESI+) $[M-Br]^+$ calc.: 257.0184, found: 257.0188.

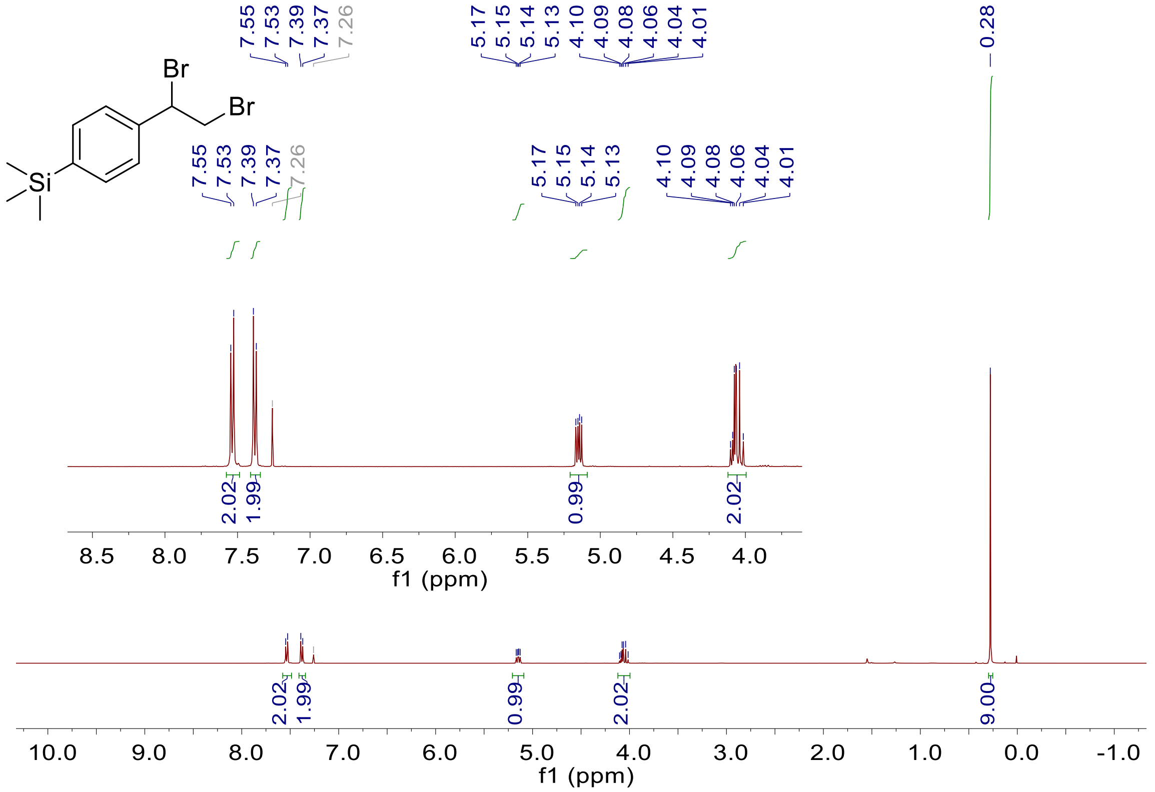

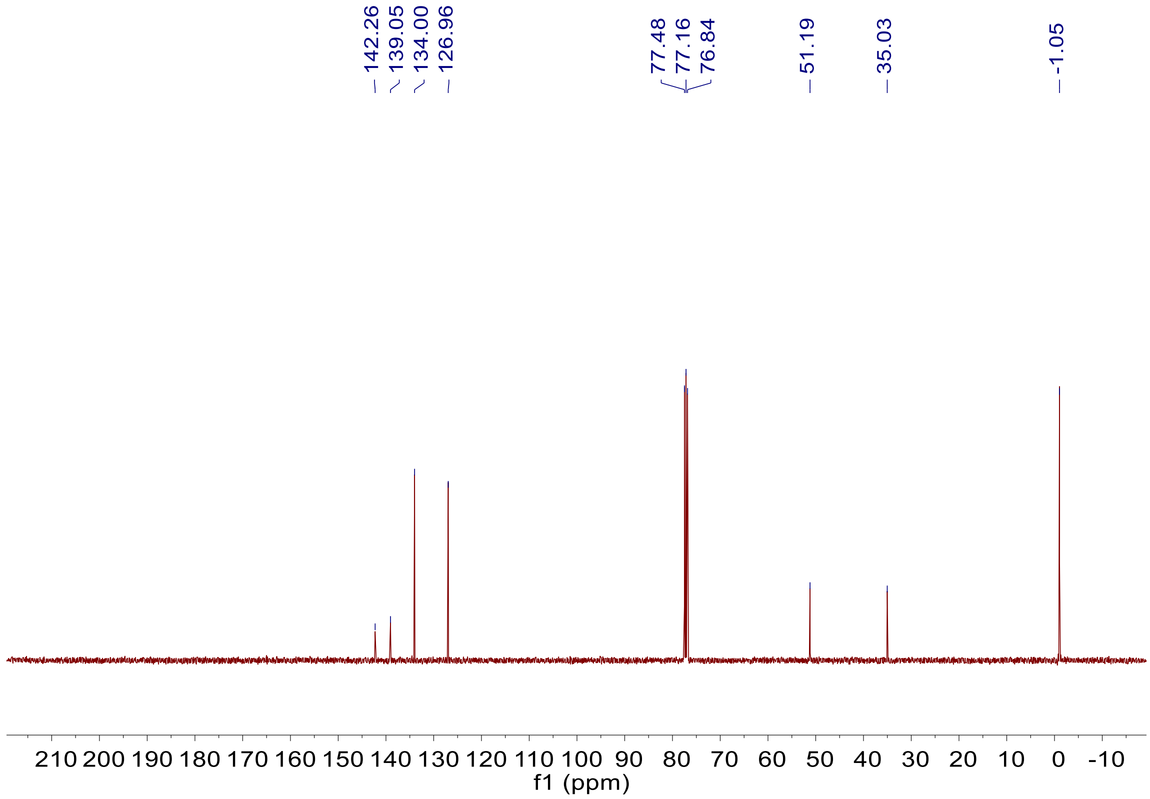

2-(4-(1,2-dibromoethyl)phenyl)-4,4,5,5-tetramethyl-1,3,2-dioxaborolane (19a)

General procedure 1: Using 0.75 mmol DBE as the donor, after **43 h**, the mixture was purified by column chromatography (PE/EtOAc=50:1) yielding the title compound (19.4 mg, yield: 50 %). HRMS for $C_{14}H_{19}BBr_2O_2$ (ESI+) $[M{-}Br]^+$ calc.: 311.0641, found: 311.0649.

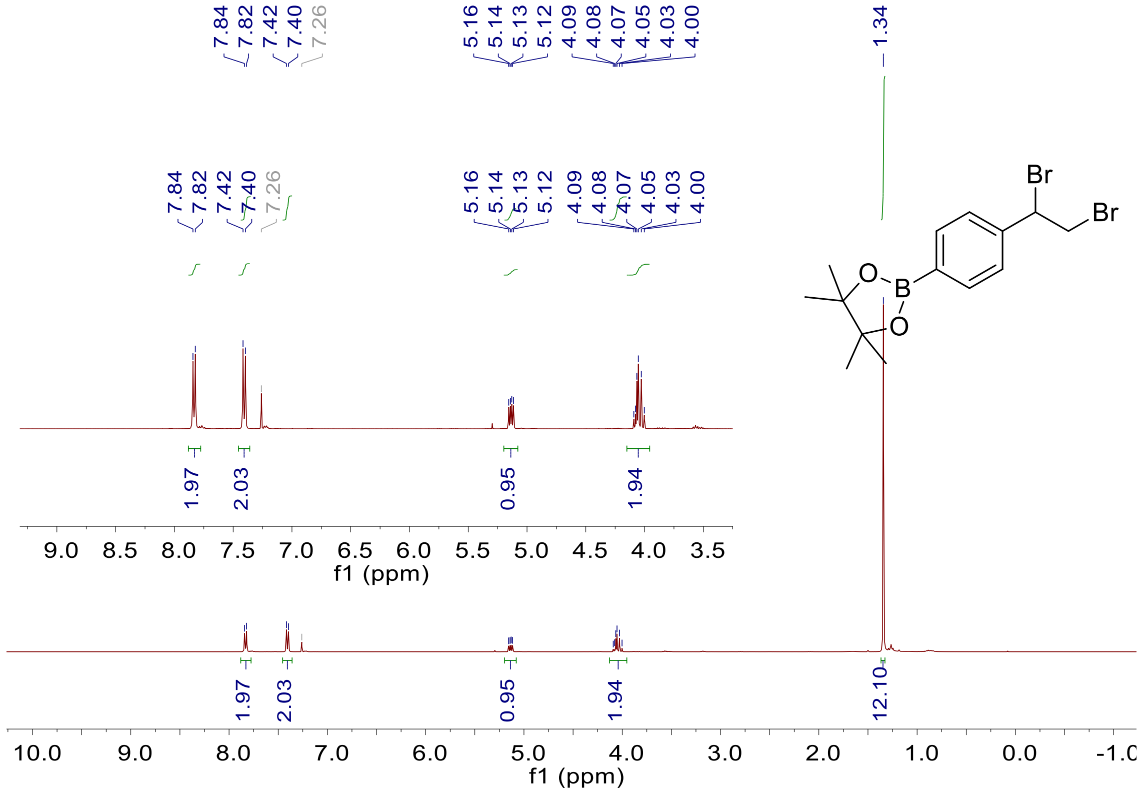

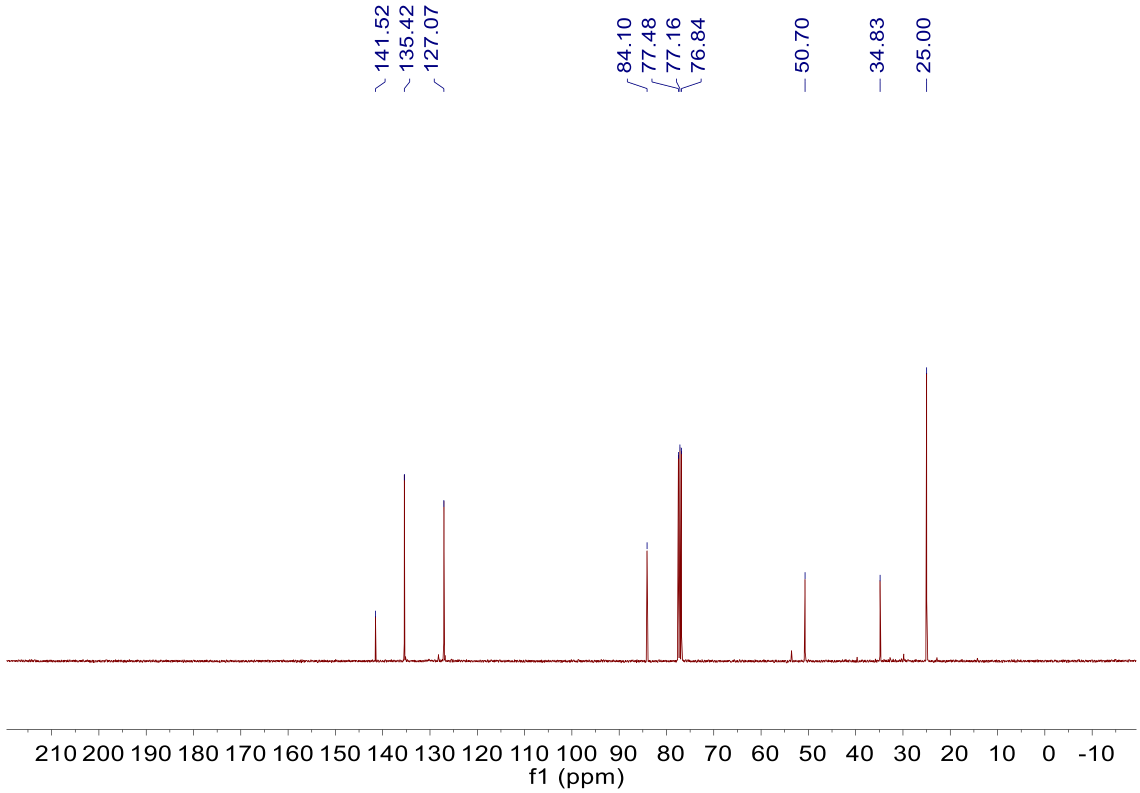

4-(1,2-dibromovinyl)-1,1'-biphenyl (20a)

General procedure 2: Using 0.5 mmol DBE as the donor, after **48 h**, the mixture was purified by column chromatography (PE/EtOAc=100:1) yielding the title compound (10.2 mg, yield: 29 %). HRMS for $C_{14}H_{10}Br_2$ (ESI+) $[M+H]^+$ calc.: 338.9207, found: 338.9109.

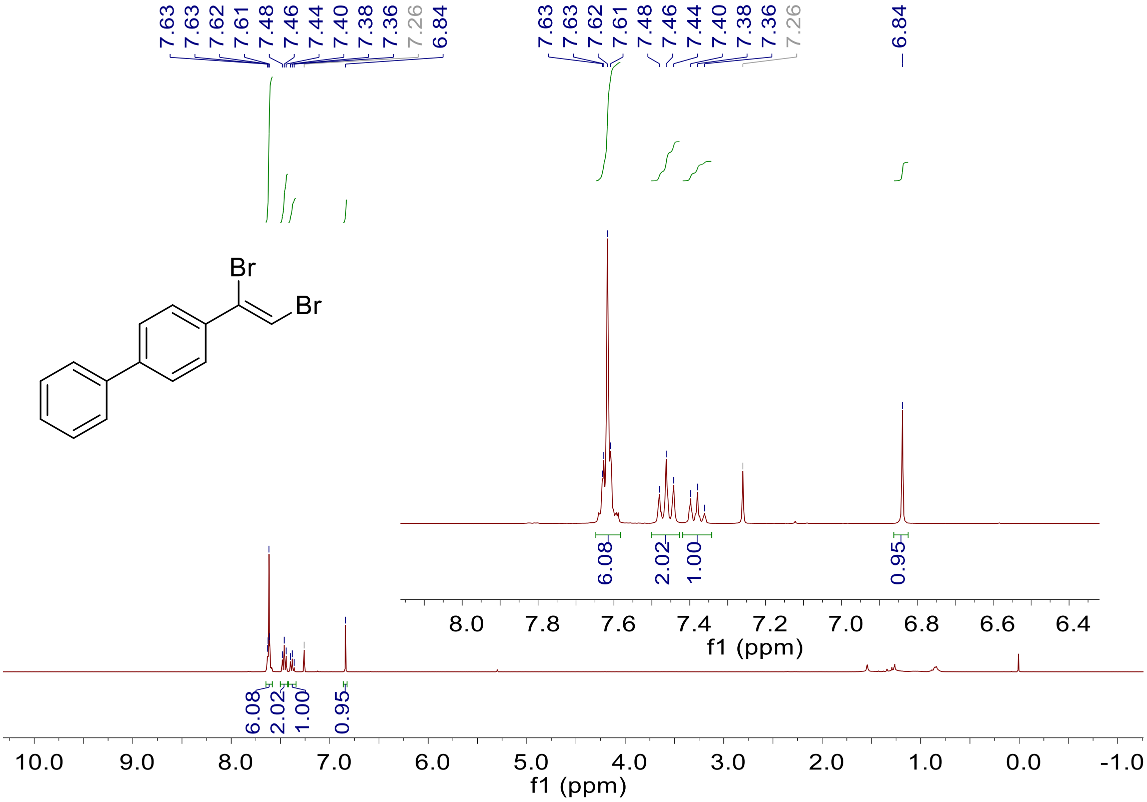

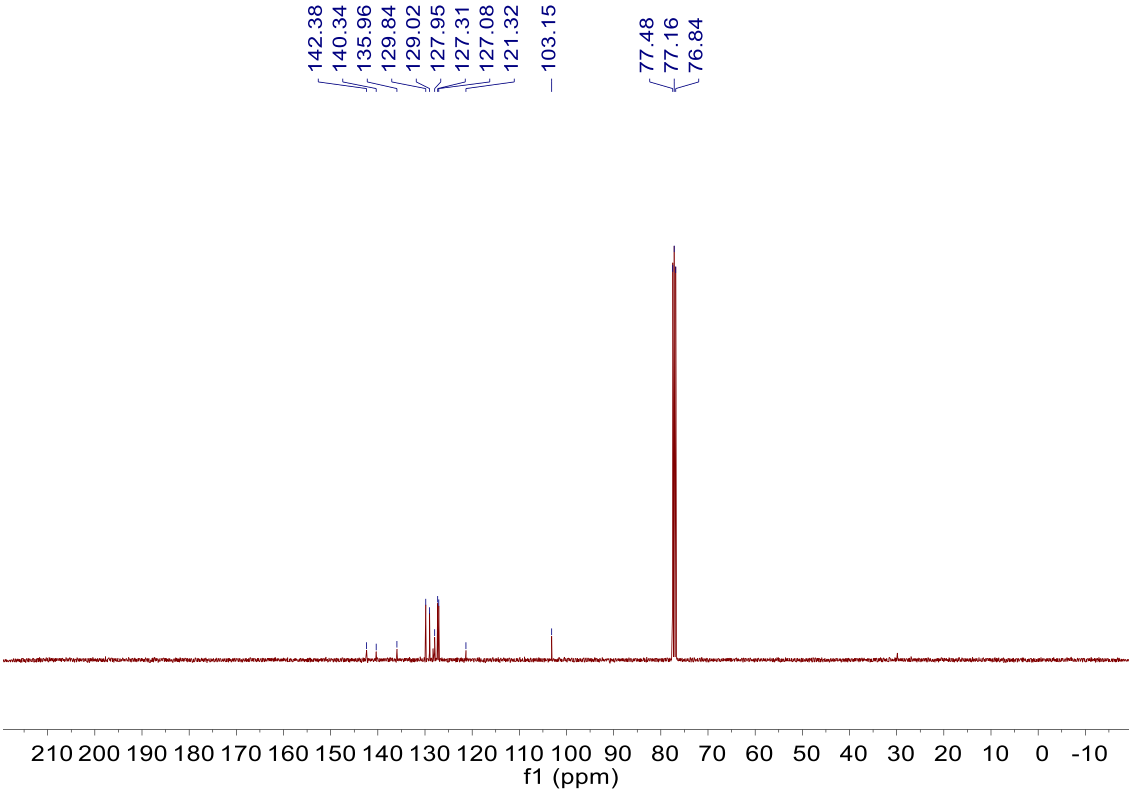

1-(1,2-dibromovinyl)-4-fluorobenzene (21a)

General procedure 2: Using 0.5 mmol DBE as the donor, after **36 h**, the mixture was purified by column chromatography (PE/EtOAc=100:1) yielding the title compound (13.4 mg, yield: 48 %). HRMS for $C_8H_5Br_2F$ (ESI+) $[M+H]^+$ calc.: 280.8800, found: 280.8794.

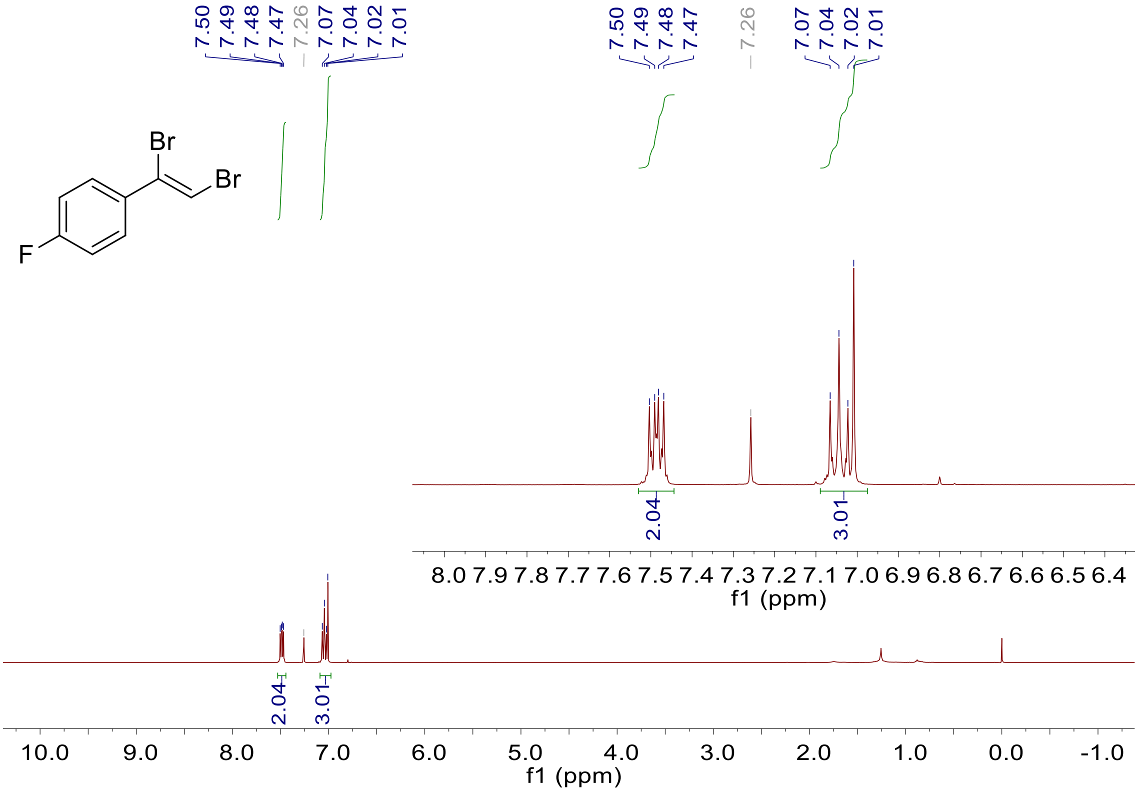

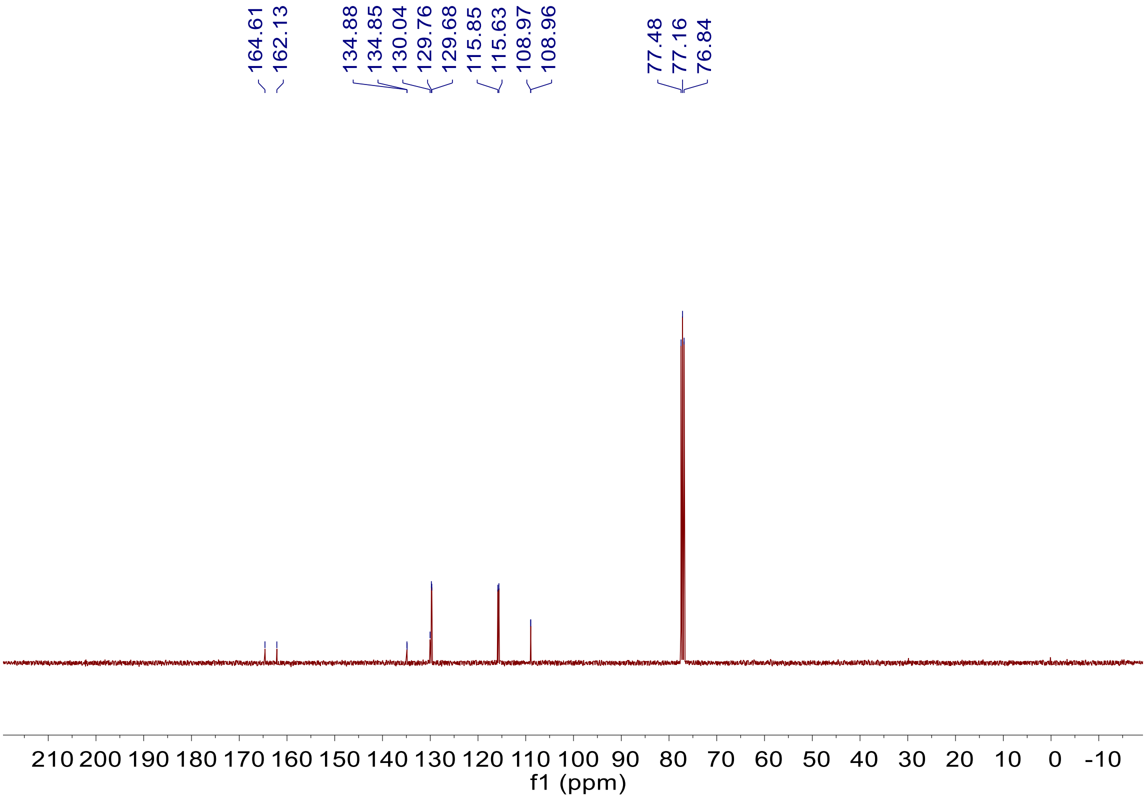

4-vinylphenyl 3-nitrobenzoate (22a)

General procedure 2: Using 1 mmol 1,1,2-tribromoethane as the donor, after **48** h, the mixture was purified by column chromatography (PE/EtOAc=5:1) yielding the title compound (36.2 mg, yield: 85 %). HRMS for $C_{15}H_{11}Br_2NO_4$ (ESI+) $[M-Br]^+$ calc.: 347.9871, found: 347.9860.

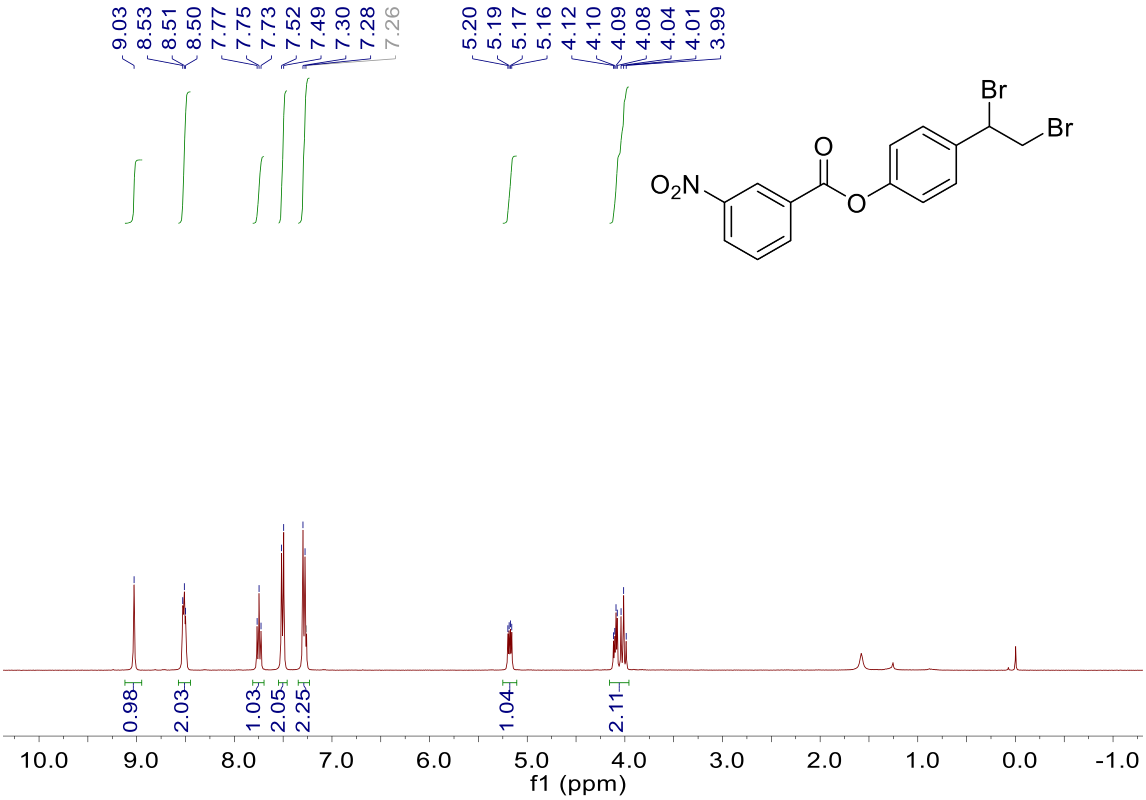

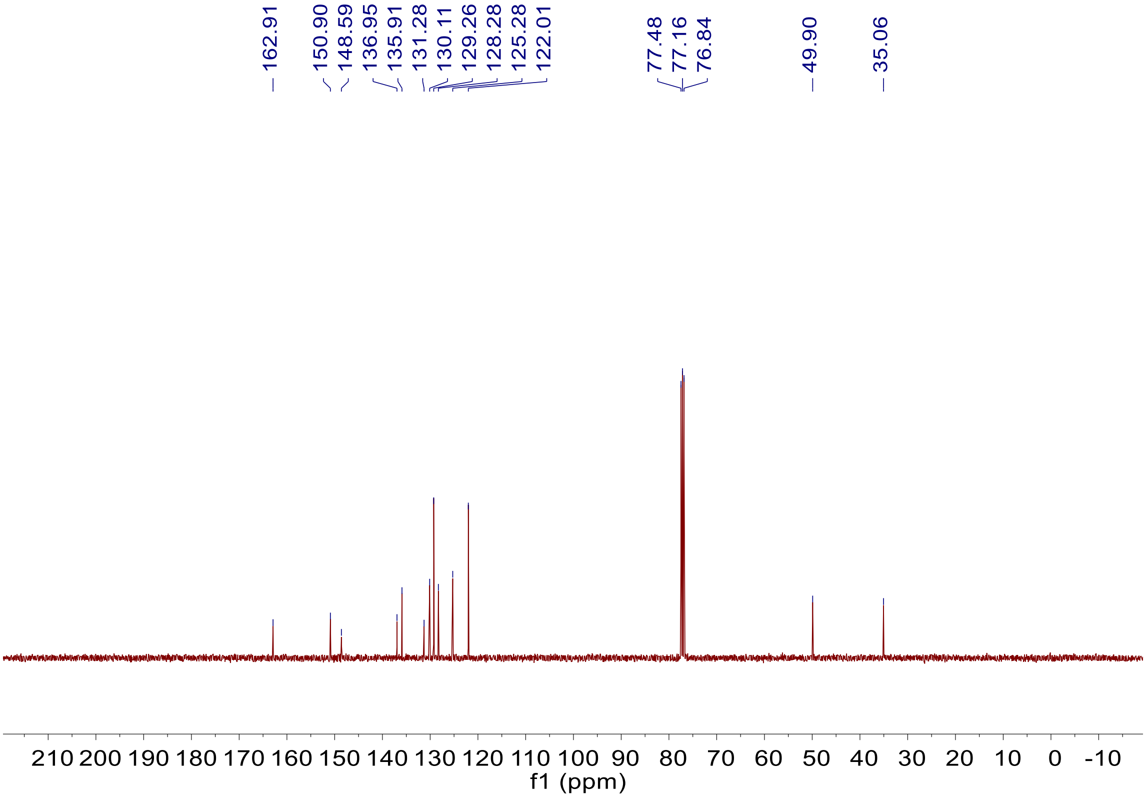

4-(1,2-dibromoethyl)benzaldehyde (23a)

General procedure 2: Using 0.50 mmol DBE as the donor, after **36 h**, the mixture was purified by column chromatography (PE/EtOAc=8:1) yielding the title compound (17.5 mg, yield: 60 %). HRMS for $C_9H_8Br_2O$ (ESI+) $[M-Br]^+$ calc.: 210.9759, found: 210.9751.

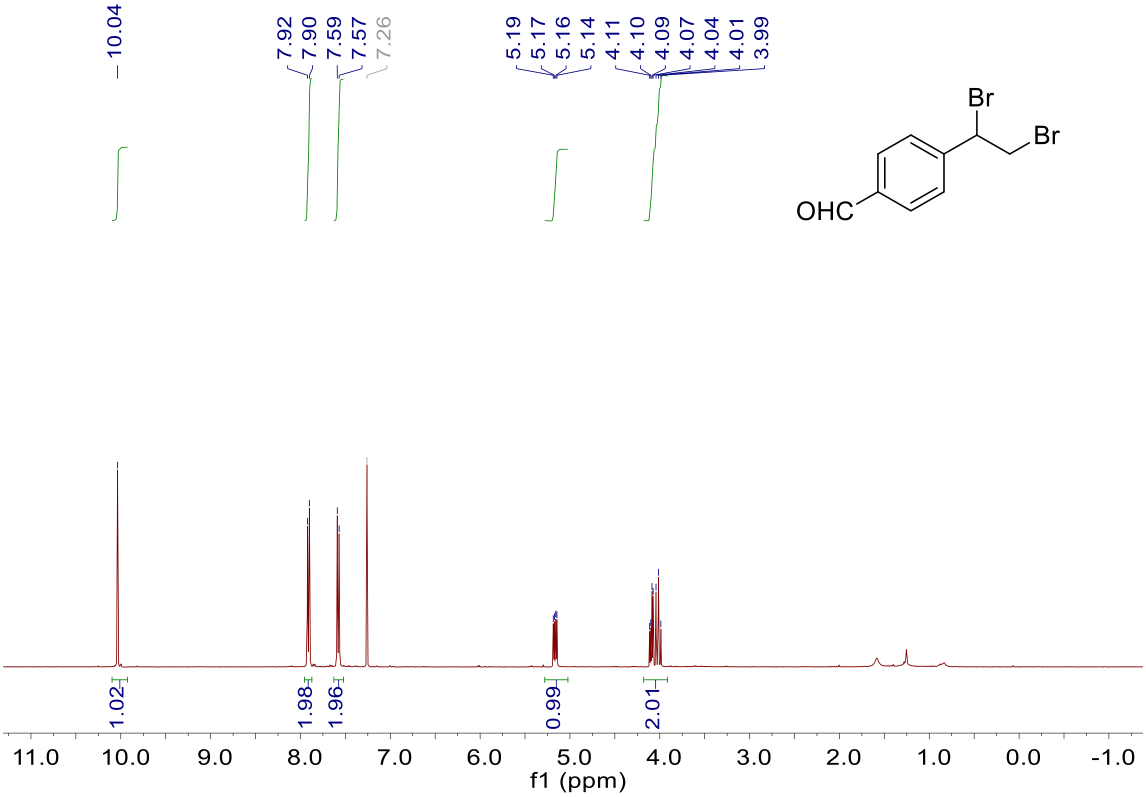

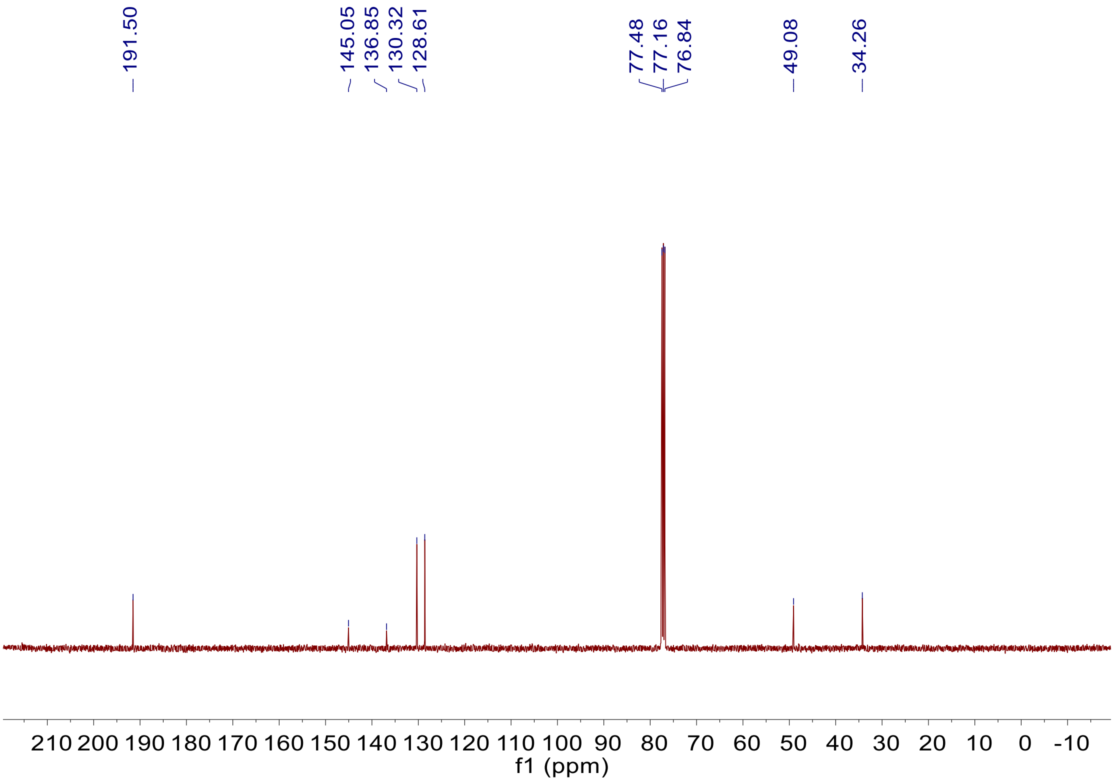

4-(1,2-dibromoethyl)phenyl 4-(4-nitrophenyl)butanoate (24a)

General procedure 2: Using 0.5 mmol DBE as the donor, after **48** h, the mixture was purified by column chromatography (PE/EtOAc=5:1) yielding the title compound (29.9 mg, yield: 64 %). HRMS for $C_{18}H_{17}Br_2NO_4$ (ESI+) $[M{-}Br]^+$ calc.: 392.0320, found: 392.0313.

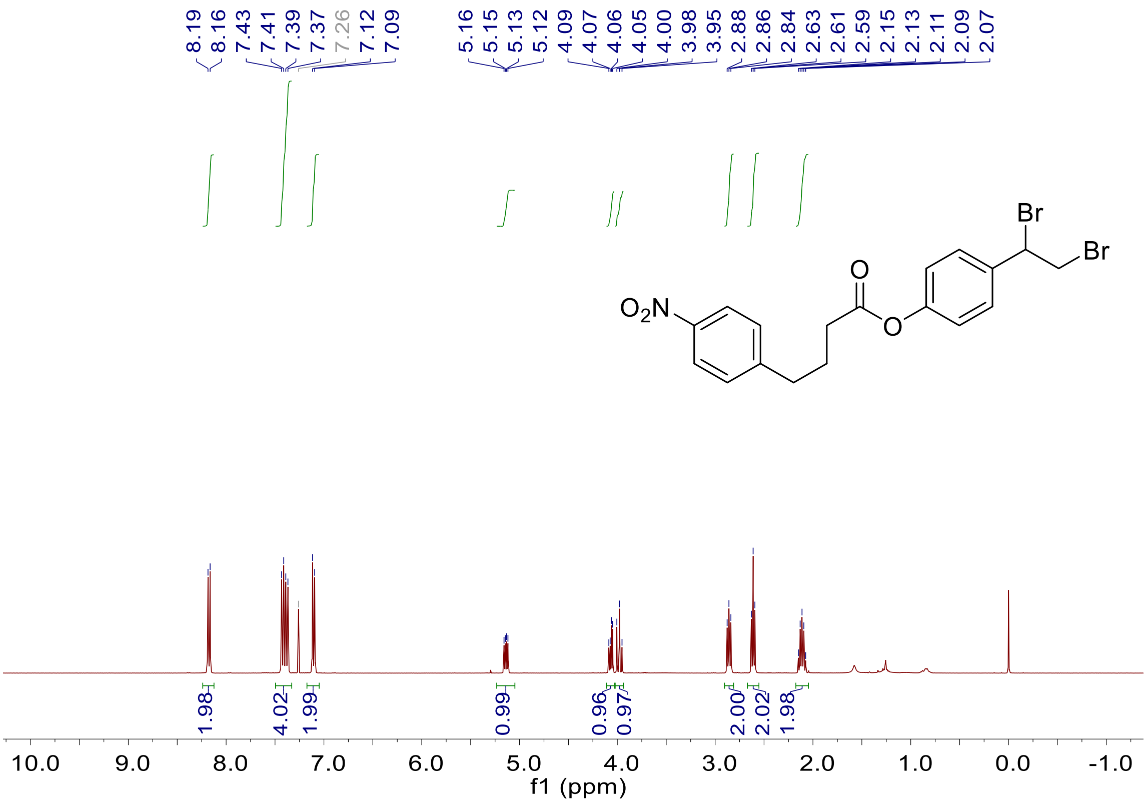

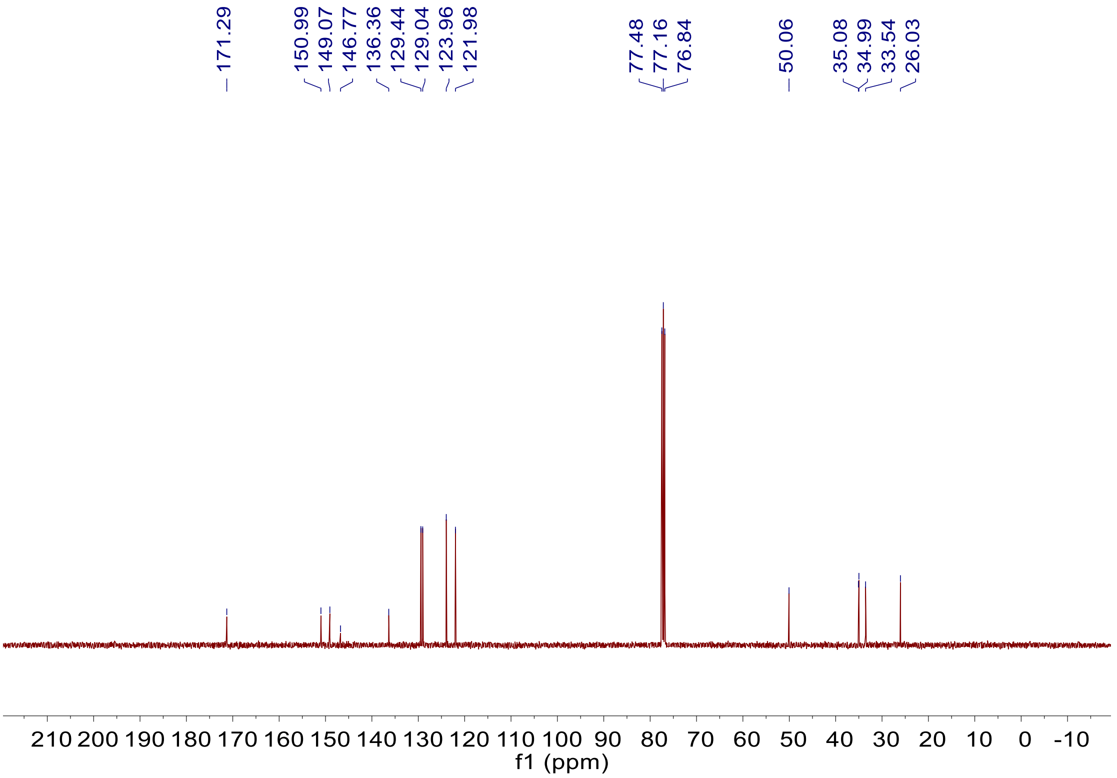

4-(1,2-dibromoethyl)phenyl 1,5-dimethyl-1H-pyrazole-4-carboxylate (25a)

General procedure 2: Using 0.5 mmol DBE as the donor, after **48** h, the mixture was purified by column chromatography (PE/EtOAc=2:1) yielding the title compound (28.0 mg, yield: 70 %). HRMS for $C_{14}H_{14}Br_2N_2O_2$ (ESI+) $[M+Na]^+$ calc.: 424.9299, found: 424.9299.

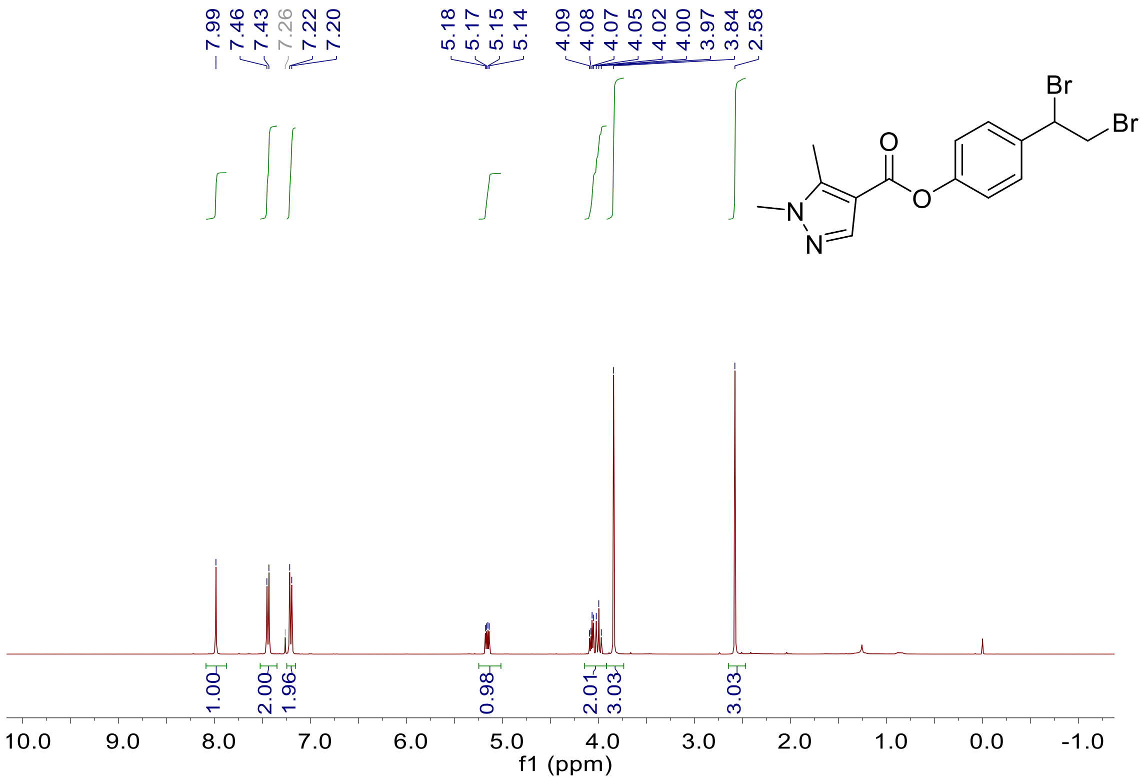

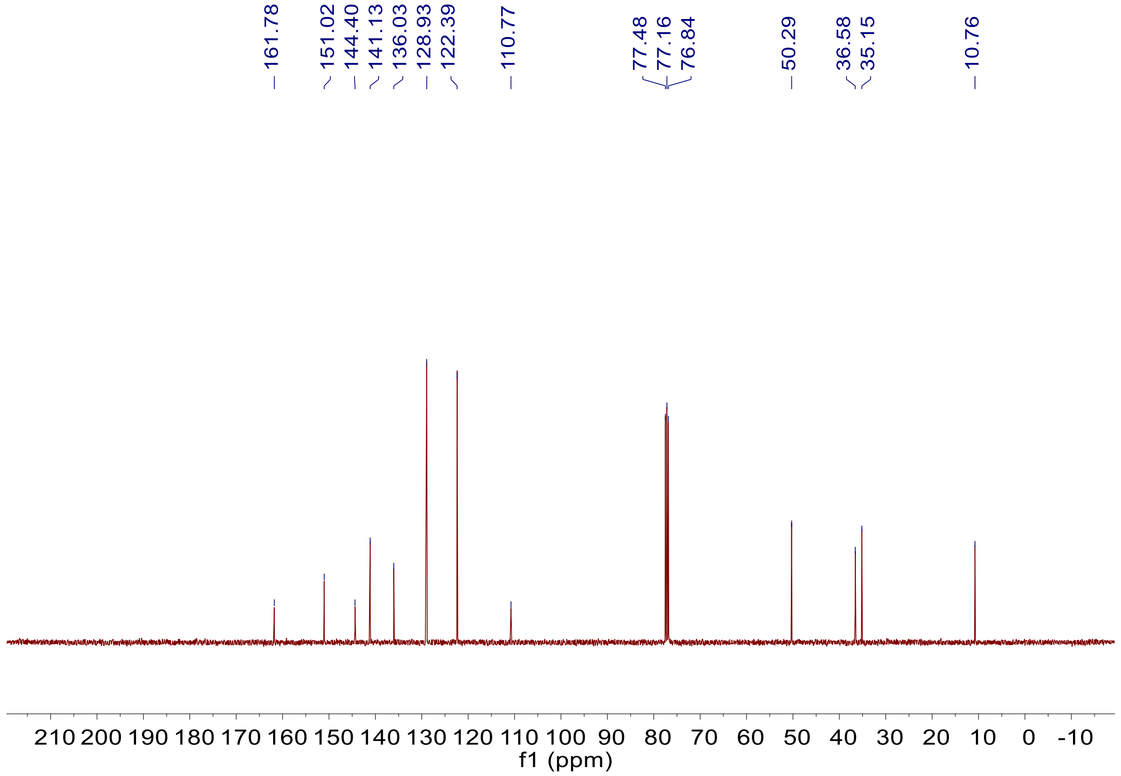

4-(1,2-dibromoethyl)phenyl 6-methoxynicotinate (26a)

General procedure 2: Using 0.5 mmol DBE as the donor, after **48** h, the mixture was purified by column chromatography (PE/EtOAc=5:1) yielding the title compound (21.9 mg, yield: 53 %). HRMS for $C_{15}H_{13}Br_2NO_3$ (ESI+) $[M+H]^+$ calc.: 415.9320, found: 415.9311.

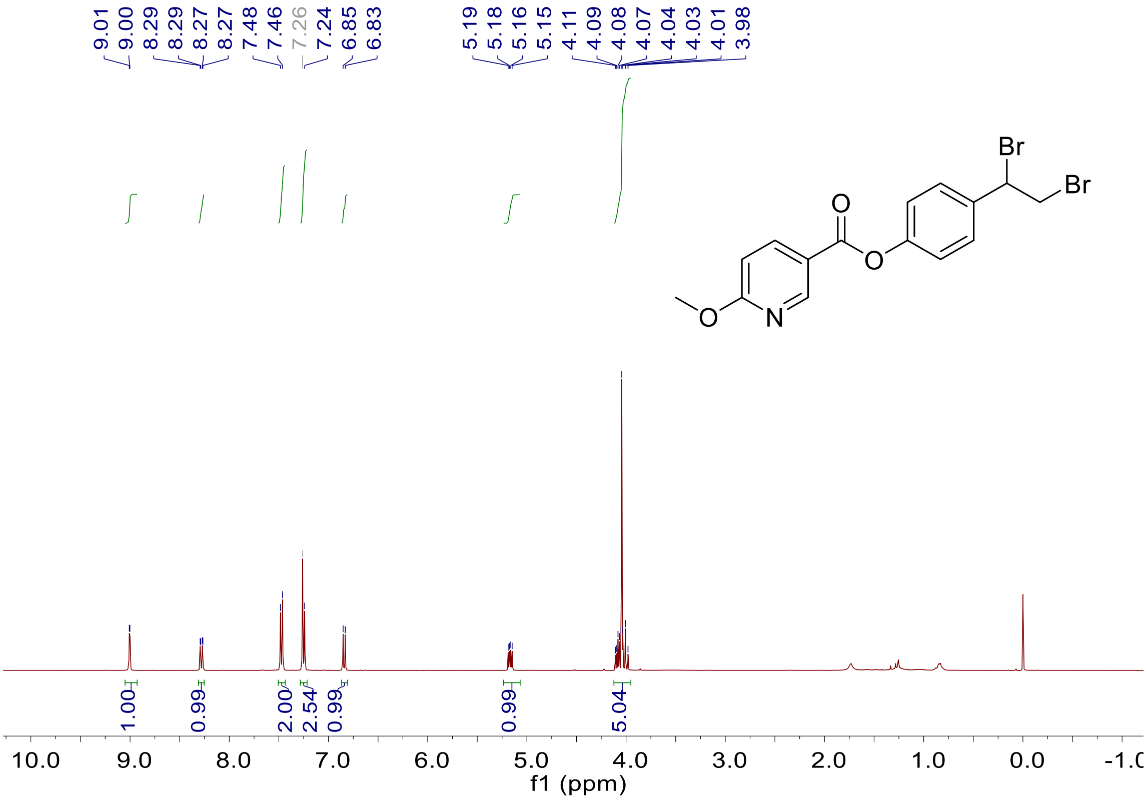

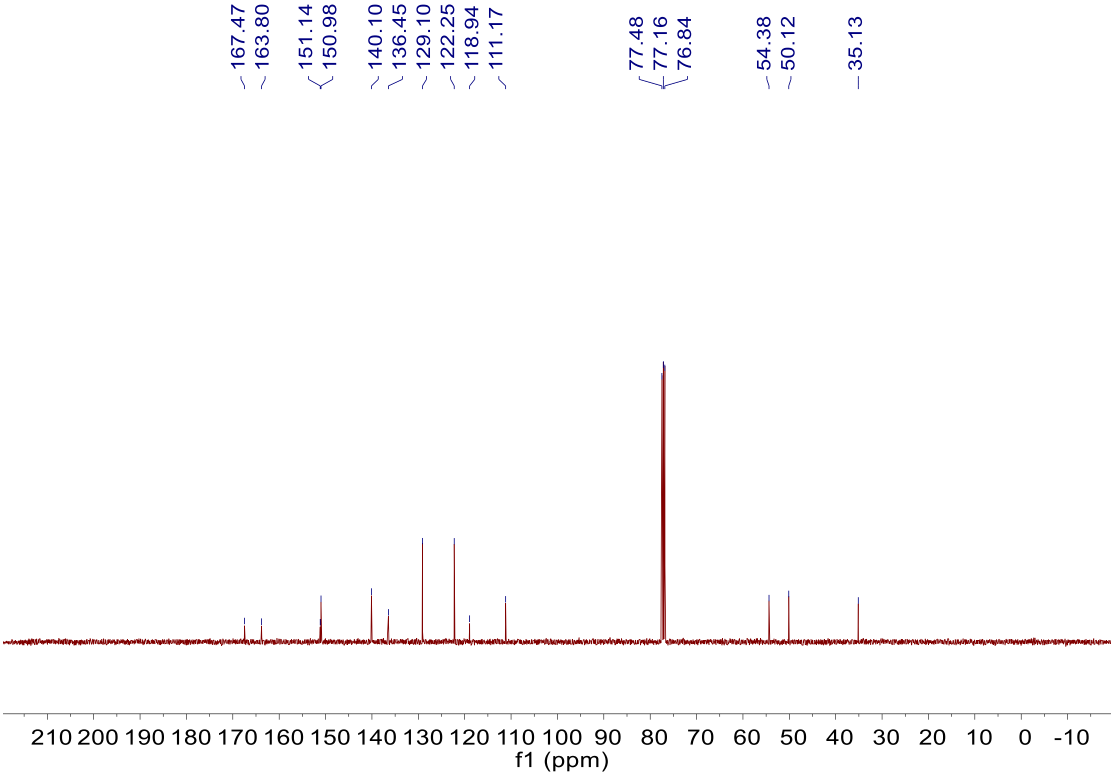

4-(1,2-dibromoethyl)phenyl pyrimidine-5-carboxylate (27a)

General procedure 2: Using 0.5 mmol DBE as the donor, after **48** h, the mixture was purified by column chromatography (DCM/MeOH=50:1) yielding the title compound (27.3 mg, yield: 71 %). HRMS for $C_{13}H_{10}Br_2N_2O_2$ (ESI+) $[M+H]^+$ calc.: 386.9167, found: 386.9165.

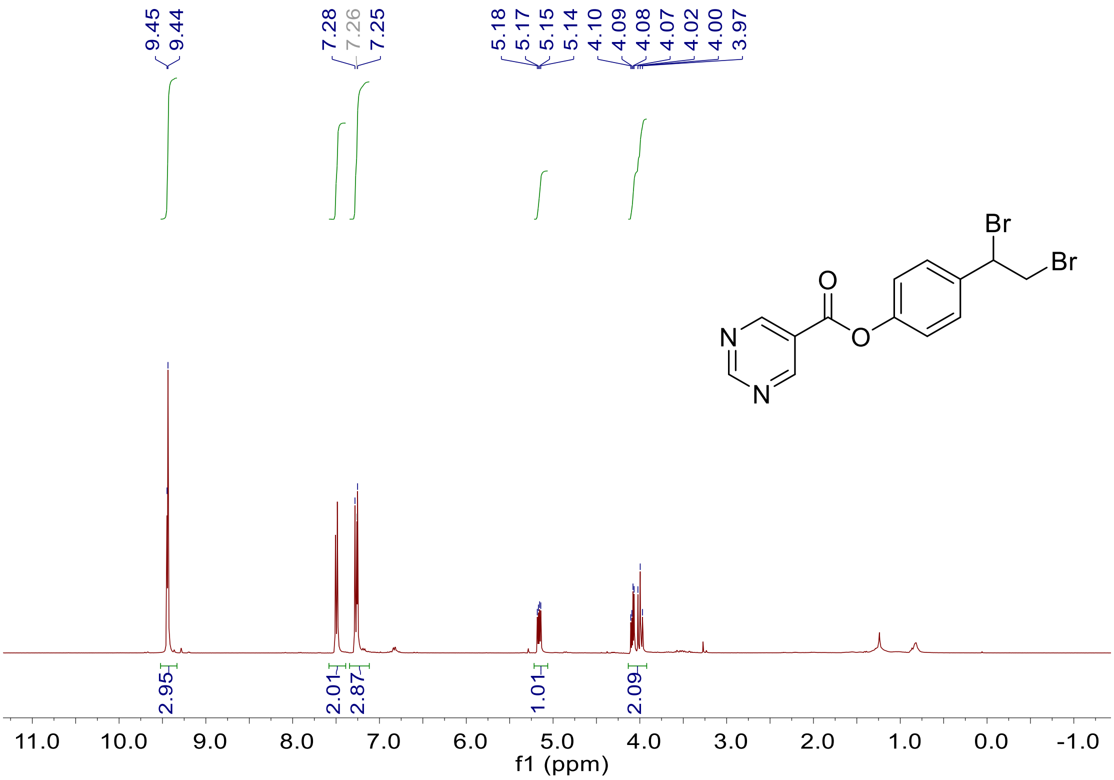

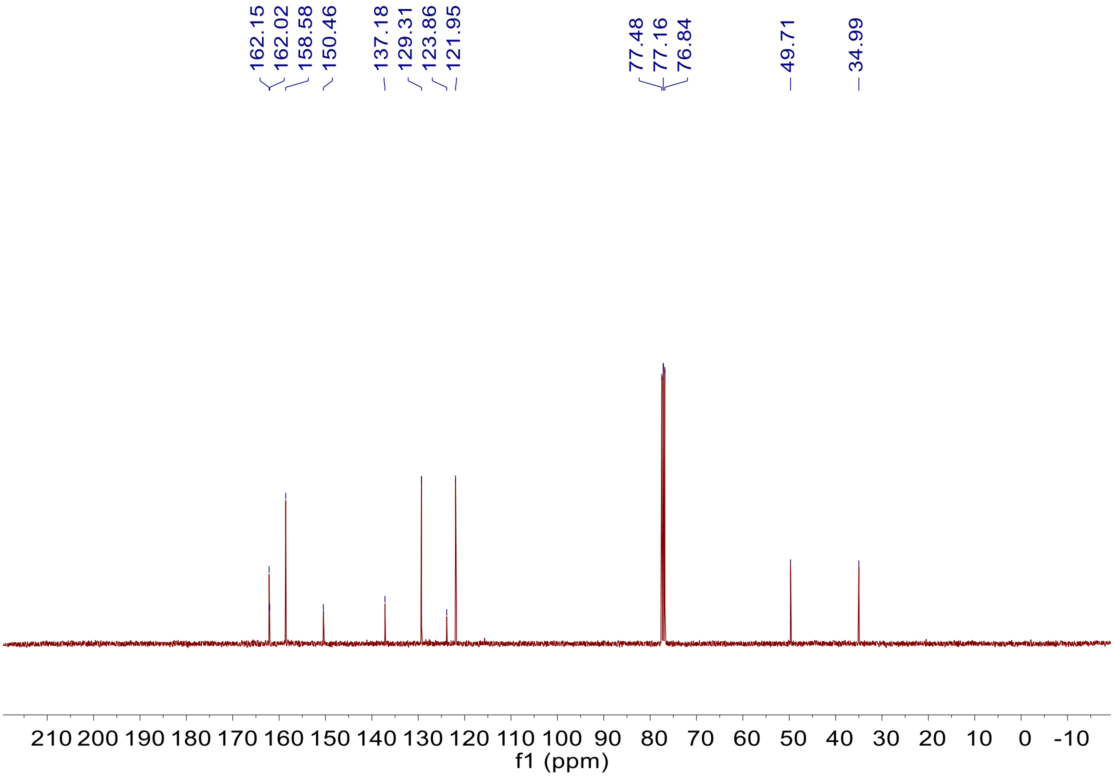

4-(1,2-dibromoethyl)phenyl thiophene-2-carboxylate (28a)

General procedure 2: Using 0.5 mmol DBE as the donor, after **36** h, the mixture was purified by column chromatography (PE/EtOAc=20:1) yielding the title compound (24.7 mg, yield: 64 %). HRMS for $C_{13}H_{10}Br_2O_2S$ (ESI+) $[M-Br]^+$ calc.: 308.9585, found: 308.9574.

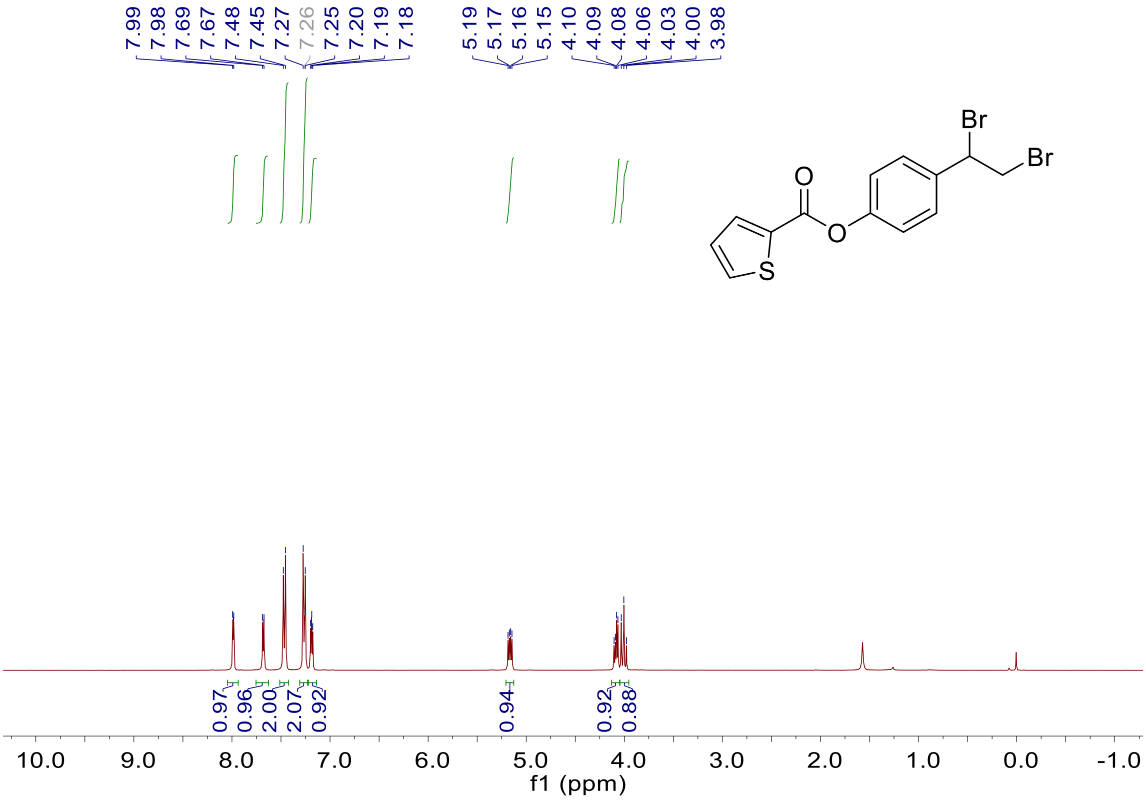

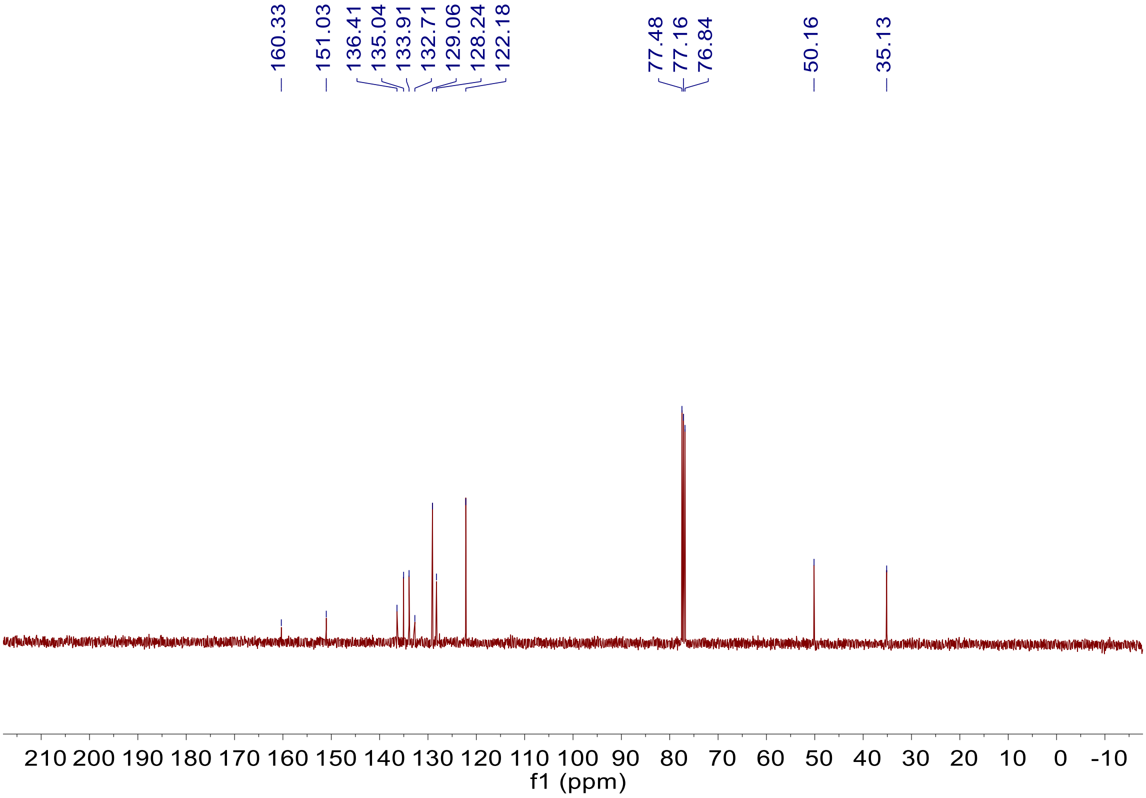

4-(1,2-dibromoethyl)phenyl furan-2-carboxylate (29a)

General procedure 2: Using 0.5 mmol DBE as the donor, after **36** h, the mixture was purified by column chromatography (PE/EtOAc=5:1) yielding the title compound (29.7 mg, yield: 80 %). HRMS for $C_{13}H_{10}Br_2O_3$ (ESI+) $[M+NH_4]^+$ calc.: 391.9320, found: 391.9315.

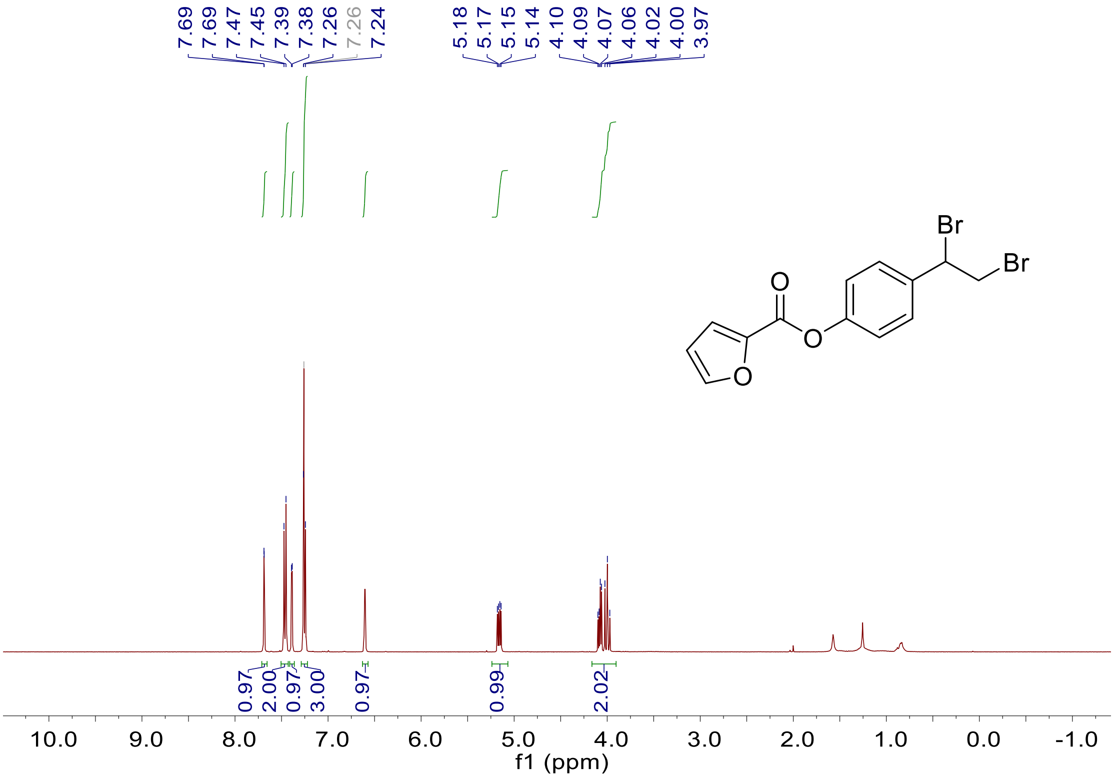

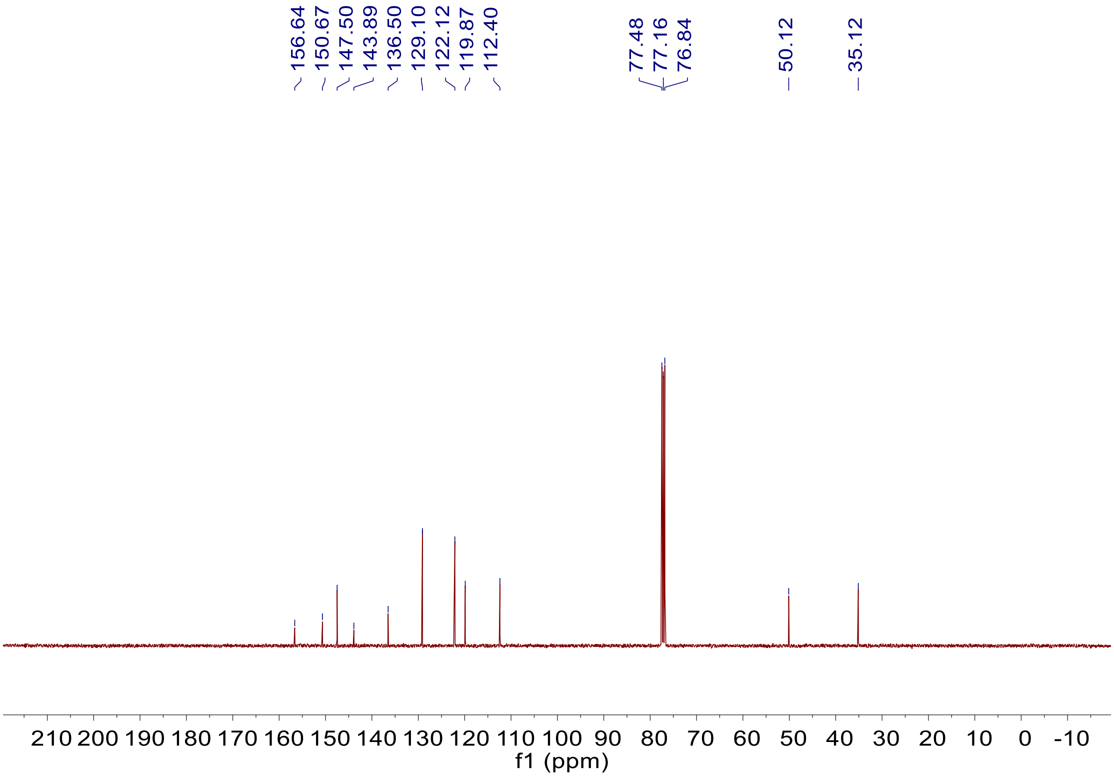

(2,3-dibromo-2-methylpropyl)benzene (10a)

General procedure 2: Using 0.5 mmol DBE as the donor, after **24 h**, a certain amount of mesitylene as internal standard compounds and DMSO-d_6 as solvents were added. Removing the QD, the reaction solution was filtered and subjected to GC, GC-MS and ^{1}H-NMR analysis. HRMS for $C_{10}H_{12}Br_2$ (ESI+) $[M+H]^+$ calc.: 292.9364, found: 292.9321.

1,2-dibromocyclohexane (11a)

General procedure 1: Using 0.5 mmol DBE as the donor, after **35 h**, a certain amount of n-dodecane as internal standard compounds was added. Removing the QD, the reaction solution was filtered and subjected to GC and GC-MS analysis. MS for $C_6H_{10}Br_2$ (EI+) $[M-Br]^+$ calc.: 161.0, found: 161.2.

22. Supplementary references

1. Li, Y. L. et al. Tunable photocatalytic two-electron shuttle between paired redox sites on halide perovskite nanocrystals. *ACS Catal.* **12,** 5903–5910 (2022).
2. Dong, X. C., Roeckl, J. L., Waldvogel, S. R. & Morandi, B. Merging shuttle reactions and paired electrolysis for reversible vicinal dihalogenations. *Science* **371**, 507–514 (2021).
3. Parobek, D., Dong, Y. T., Qiao, T., Rossi, D. & Son, D. H. Photoinduced anion exchange in cesium lead halide perovskite nanocrystals. *J. Am. Chem. Soc.* **139**, 4358−4361 (2017).
4. Caputo, J. A. et al. General and efficient C-C bond forming photoredox catalysis with semiconductor quantum dots. *J. Am. Chem. Soc.* **139**, 4250–4253 (2017).
5. Liu, Z. et al. Heavy metal ternary halides for room-temperature x-ray and gamma-ray detection, *Proc. SPIE* **8852**, 88520A (2013).
6. Martin, J. S. et al. A nanocrystal catalyst incorporating a surface bound transition metal to induce photocatalytic sequential electron transfer events. *J. Am. Chem. Soc.* **143**, 11361–11369 (2021).
7. Vitoreti, A. B. F. et al. Study of the partial substitution of Pb by sn in Cs–Pb–Sn–Br nanocrystals owing to obtaining stable nanoparticles with excellent optical properties. *J. Phys. Chem. C* **122**, 14222– 14231(2018).
